# BORIS/CTCFL epigenetically reprograms clustered CTCF binding sites into alternative transcriptional start sites

**DOI:** 10.1186/s13059-024-03175-0

**Published:** 2024-01-31

**Authors:** Elena M. Pugacheva, Dharmendra Nath Bhatt, Samuel Rivero-Hinojosa, Md Tajmul, Liron Fedida, Emma Price, Yon Ji, Dmitri Loukinov, Alexander V. Strunnikov, Bing Ren, Victor V. Lobanenkov

**Affiliations:** 1 Molecular Pathology Section, Laboratory of Immunogenetics, National Institute of Allergy and Infectious Diseases, National Institutes of Health, Bethesda, MD 20892 USA; 2 Center for Cancer and Immunology Research, Children’s National Research Institute, Washington, DC 20010 USA; 3 Guangzhou Institutes of Biomedicine and Health, Molecular Epigenetics Laboratory, 190 Kai Yuan Avenue, Science Park, Guangzhou, 510530 China; 4 Ludwig Institute for Cancer Research, 9500 Gilman Drive, La Jolla, CA 92093 USA; 5 Department of Cellular and Molecular Medicine, Center for Epigenomics, Moores Cancer Center and Institute of Genomic Medicine, University of California, San Diego School of Medicine, La Jolla, CA 92093-0653 USA

**Keywords:** CTCF, CTCFL/BORIS, Alternative transcription, Cancer, Germ cells

## Abstract

**Background:**

Pervasive usage of alternative promoters leads to the deregulation of gene expression in carcinogenesis and may drive the emergence of new genes in spermatogenesis. However, little is known regarding the mechanisms underpinning the activation of alternative promoters.

**Results:**

Here we describe how alternative cancer-testis-specific transcription is activated. We show that intergenic and intronic CTCF binding sites, which are transcriptionally inert in normal somatic cells, could be epigenetically reprogrammed into active de novo promoters in germ and cancer cells. BORIS/CTCFL, the testis-specific paralog of the ubiquitously expressed CTCF, triggers the epigenetic reprogramming of CTCF sites into units of active transcription. BORIS binding initiates the recruitment of the chromatin remodeling factor, SRCAP, followed by the replacement of H2A histone with H2A.Z, resulting in a more relaxed chromatin state in the nucleosomes flanking the CTCF binding sites. The relaxation of chromatin around CTCF binding sites facilitates the recruitment of multiple additional transcription factors, thereby activating transcription from a given binding site. We demonstrate that the epigenetically reprogrammed CTCF binding sites can drive the expression of cancer-testis genes, long noncoding RNAs, retro-pseudogenes, and dormant transposable elements.

**Conclusions:**

Thus, BORIS functions as a transcription factor that epigenetically reprograms clustered CTCF binding sites into transcriptional start sites, promoting transcription from alternative promoters in both germ cells and cancer cells.

**Supplementary Information:**

The online version contains supplementary material available at 10.1186/s13059-024-03175-0.

## Background

Cell fate specialization is driven, at least in part, by pioneer transcription factors that bind to nucleosomal DNA to initiate cell type-specific gene transcription programs. The differentiation of male germ cells during spermatogenesis has a more complex transcriptional program than any other cell types [1]; it features the widespread expression of over 90% of all protein-coding genes, as well as expression of an extensive set of small and long noncoding RNAs, pseudogenes, and transposable elements (TE) [2–7]. Another characteristic of spermatogenesis contributing to its complexity is the prevalent use of alternative promoters [8]. Alternative promoter usage results in increased transcriptomic and proteomic diversity [9], leading to a highly specialized transcriptional program of male germ cell differentiation. The pervasive transcription in male germ cells is facilitated by epigenetic changes, such as DNA demethylation, gain of active histone post-translational modifications, and extensive open chromatin states [5]. It has been suggested that the promiscuous transcription during spermatogenesis may be beneficial for the emergence of new genes during evolution [5]. Another possible explanation of the widespread transcription during spermatogenesis is to reduce mutation rates by the transcriptional scanning mechanism [7]. The same testis-specific transcriptional program is often aberrantly activated in many types of cancer [10].

Cancer cells often possess some of the same characteristics as male germ cells, including the expression of testis-specific genes, unchecked proliferation, derepression of retrotransposons, and the pervasive usage of alternative promoters [10–12]. The so called cancer-testis (CT) genes, which are normally expressed during spermatogenesis but aberrantly activated in cancer cells, are responsible for production of cancer-testis antigens (CTA). Expression of CT genes correlates with tumorigenesis, resistance to chemotherapies, and is considered to be one of the hallmarks of carcinogenesis [10]. Normally restricted to testis, expression of CT genes in cancer cells makes them reliable prognostic markers and attractive targets for immunotherapies [13].

The search for the underlying molecular mechanisms of testis-specific transcriptional activity in cancers is a subject of ongoing research. Particularly, there is a growing number of publications that identified BORIS (Brother of Regulator of Imprinted Sites), also known as CTCFL (CTCF-Like), as a diagnostic and prognostic marker for multiple cancer types [14–17]. BORIS is a CTA that is frequently aberrantly upregulated in cancer cells of non-germline origins [15]. BORIS expression in cancer cells is associated with stemness [14], invasiveness [18, 19], increased epithelial to mesenchymal transition (EMT) [20], resistance to anticancer treatment [21], activation of oncogenes or other CTAs [22, 23], and regulation of humanoid-specific SVA transposable elements [24]. Conditional BORIS expression during early mouse embryogenesis leads to multiple organ pathologies, growth retardation, and neonatal death [25]. Conversely, the downregulation of BORIS expression in BORIS-positive cancer cells results in either cell death and differentiation [22] or in a less tumorigenic phenotype [21, 26].

Among CTA genes, BORIS occupies a truly exceptional position, as it is the sole paralogue of CTCF (CCCTC-Binding Factor) [16, 27, 28], a global regulator of genome organization and gene expression [29–33]. CTCF divides genomes into topologically associated domains (TADs) by restricting cohesin-mediated extrusion of chromatin loops owing to the activity of its N-terminal domain [30, 34–36]. CTCF is also involved in the direct regulation of gene transcription, alternative splicing, imprinting, chromatin insulation, X chromosome inactivation, and DNA repair [37–41]. Both CTCF and BORIS share a nearly identical 11 Zinc Finger (ZF) DNA-binding domain but differ in their N- and C-termini [16, 42]. As a result of high homology in the DNA-binding domains, CTCF and BORIS can recognize and bind to similar DNA sequences; however, the functional outcome of their binding differs dramatically [22, 34, 36, 43]. In contrast to the ubiquitously expressed CTCF, BORIS expression is strictly restricted to male germ cells in virtually all vertebrates [15, 27, 44]. When CTCF and BORIS are co-expressed, essentially only in germline and cancer cells, they tend to form a heterodimer at clustered (double) CTCF binding sites, which encompass two or more CTCF binding consensus sequences (2xCTSes) [22, 43].

We recently demonstrated that CTCF and BORIS heterodimerization at clustered CTCF binding sites drives the testis-specific transcriptional program at different stages of spermatogenesis [43]. A similar testis-specific program is aberrantly activated in many types of cancers, coinciding with BORIS activation in the same cells [14, 22, 23, 45]. Thus, the primary goal of this study was to determine the functional role of BORIS in carcinogenesis and to elucidate the mechanism of the subsequent initiation of testis-specific transcription in somatic cells, following aberrant BORIS activation. We combine the genome-wide mapping of epigenetic marks with the analysis of global gene expression profiles with respect to CTCF and BORIS chromatin occupancies. Using various cell models with endogenous and ectopic BORIS expression, we show that hundreds of testis-specific transcripts are aberrantly expressed in cancer cells from CTCF and BORIS co-bound transcriptional start sites. We demonstrate that BORIS activates transcription of testis-specific promoters in cancers by epigenetically reprogramming transcriptionally inert CTCF binding sites into active transcriptional units.

## Results

### BORIS binding to intronic CTCF binding sites activates cancer-testis-specific transcription from alternative promoters of GAL3ST1 and FER genes

The aberrant activation of BORIS in cancers is associated with the derepression of testis-specific transcription [14, 23]. To understand the mechanism by which BORIS activates transcription, we first examined known targets of such regulation. We focused on the propensity of BORIS to activate alternative, testis-specific transcription from intronic loci of somatically expressed genes [9, 46]. Analysis of spermatogenesis defects in BORIS knockout mice revealed Gal3st1 as a target gene, where BORIS binding to the intronic CTCF site drives testis-specific Gal3st1 expression from alternative promoter [46, 47]. The FER gene is another example of testis-specific transcription initiated by BORIS. It has been demonstrated that BORIS binding to the FER intronic region between exons 9 and 10 results in the expression of a truncated (FERT) testis-specific form in colon carcinoma cells [9]. This is in contrast to the somatic form of FER, known as FERS [48]. Therefore, both GAL3ST1 and FERT are male germ cell-specific products of genes expressed in somatic cells and are thus good candidates through which we can study BORIS-specific regulation either in cancers or our cell model systems.

**Fig. 1:**
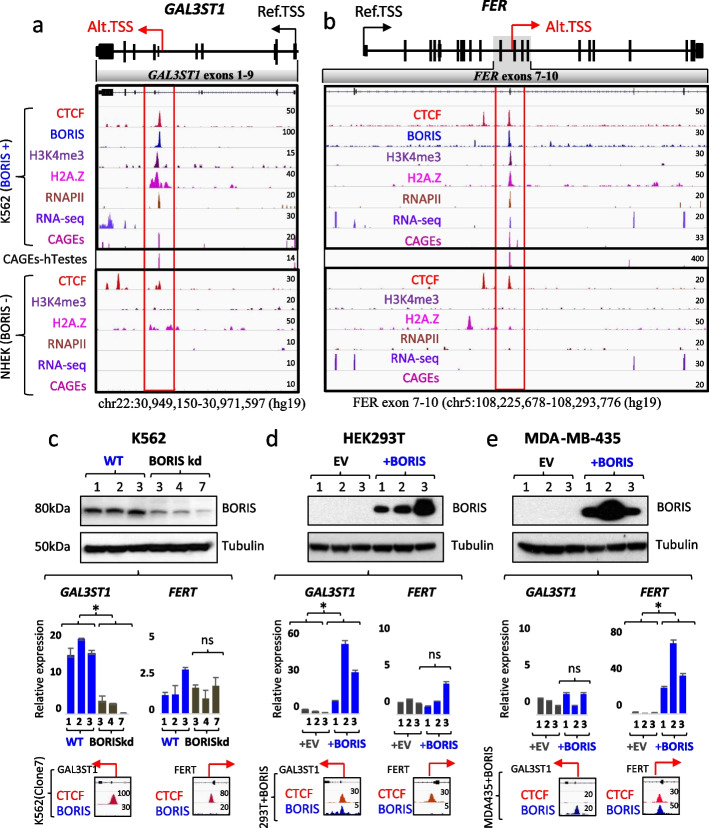
CTCF and BORIS co-binding to intronic regions of *GAL3ST1* and *FER* genes associated with cancer-testis-specific transcription. **a,b** Above: Schematic representation of the gene structure of GAL3ST1 (**a**) and FER (**b**). Arrows denote somatic (black) and testis-specific (red) TSSs. Below: In the upper part, ChIP-seq peaks illustrate CTCF (red) and BORIS (blue) co-binding in BORIS-positive (BORIS +) K562 cells across GAL3ST1 and FER (exon 7-10). The co-binding coincides with the enrichment of active histones/marks H3K4me3 (purple), H2A.Z (magenta), and RNAPII (brown). RNA-seq peaks (indigo) and CAGE-seq peaks (pink) highlight alternative transcription in K562 cells. In the lower part, CTCF binding alone in BORIS-negative (BORIS −) NHEK cells does not activate testis-specific promoters. CAGE-seq for human testes (pink) is shown between the two panels, with red boxes highlighting testis-specific TSSs. **c–e** In the upper part, Western blots from whole cell lysates show BORIS protein detection in **c** K562 wild-type (WT1-total culture, WT2 and WT3 – single-cell wild-type clones transfected with control RNA) versus BORIS knockdown (kd) K562 single-cell clones (#3,4,7), obtained by zinc finger nuclease (ZFN) treatment. **d** HEK293T and **e** MDA-MB-435 cells transfected with empty (EV) or BORIS-expressing vector. Tubulin is used as a loading control. Numbers (1,2,3) indicate different single-cell clones. The middle part displays RT-qPCR results indicating the relative expression of GAL3ST1 and FERT in K562, HEK293T, and MDA-MB-435 cell lines. Statistical analysis was performed using two-tailed Student’s *t* test (*, *p* < 0.0005). Error bars indicate mean ± SD (*n* = 3), ns – non-significant. In the bottom part, ChIP-seq peaks show CTCF and BORIS occupancy in K562 (clone#7) BORIS kd cells, HEK293T, and MDA-MB-435 cells. Abbreviations; Ref. TSS (reference transcriptional start site), Alt. TSS (alternative transcriptional start site)

We first compared epigenetic changes accompanying BORIS’s binding to the intronic sites of GAL3ST1 and FER genes in cancer cells to that in BORIS-negative cells. As a model, we used human chronic myelogenous leukemia (K562) cells, a cell line where BORIS is endogenously expressed and CTCF and BORIS binding sites have been previously mapped [22]. Combining multiple genome browser ChIP-seq tracks in K562 cells illustrated that GAL3ST1 and FERT intronic promoters are bound by both CTCF and BORIS proteins, thus classifying them as CTCF/BORIS binding sites (Fig. 1a,b). The coupled CTCF and BORIS occupancy of both intronic promoters is associated with the enrichment of RNA polymerase II subunit RPB1 (RNAPII), H2A.Z and H3K4me3 histone modifications, all well-documented characteristics of active promoters. Conversely, in BORIS-negative cells, normal human epithelial keratinocytes (NHEK), the intronic GAL3ST1 and FER testis-specific promoters are occupied by only CTCF and not associated with any marks of active transcription. This may suggest that only the co-binding of CTCF with BORIS may result in the epigenetic transformation of intronic CTCF sites into active promoters. While the expression of three coding exons of the GAL3ST1 gene could be confirmed by RNA-Seq data (Fig. 1a), the resolution of RNA-seq was not sufficient to detect the utilization of a testis-specific promoter. Deep Cap Analysis of Gene Expression (deep-CAGE), a method that determines transcription start sites (TSSs) at a genome-wide level, confirmed the initiation of transcription from the intronic CTCF/BORIS sites in the GAL3ST1 gene (Fig. 1a). Indeed, the high enrichment of the minus strand CAGE reads in close proximity to CTCF/BORIS site was observed only in the K562 cells, but not in NHEK (Fig. 1a). Similar analysis of the FER gene demonstrated that co-binding of CTCF and BORIS to the region residing in the intron between exon 9 and 10 is associated with RNAPII recruitment and chromatin remodeling consistent with active FERT transcription in the K562 cell line, but not in BORIS-negative cells (Fig. 1b). The N-terminus of the FERT isoform, which distinguishes it from the FERS isoform, is long enough to be detected by RNA-Seq as an alternative exon (Fig. 1b). Accordingly, the enrichment of the plus strand CAGE tags adjacent to the CTCF/BORIS ChIP-seq peak confirmed the initiation of FERT transcription from the intronic promoter. Additionally, by mapping TSSs in human testes with deep-CAGE, we established that the same intronic promoters are active in male germ cells (Fig. 1a,b), the testis CAGE-seq track is in between K562 and NHEK cells data).

Based on FERT and GAL3ST1 gene expression analyses in BORIS-positive (K562) and BORIS-negative (NHEK) cells, CTCF occupancy alone, or low CTCF occupancy, is not sufficient to drive the transcription from the two corresponding intronic promoters (Fig. 1a,b). To interrogate a direct role of BORIS in the activation of alternative promoters, we modulated the level of BORIS protein in several cell models and assessed its impact on transcription. First, we detected a significant downregulation of GAL3ST1 expression upon loss of BORIS binding in BORIS-depleted K562 (clones #3, 4, 7) cells (Fig. 1c; Additional file 1: Fig. S1a), where a disruption of the first coding exon of the BORIS gene by zinc finger nuclease (ZFN) treatment resulted in its severe downregulation [22], compared to the total culture of wild-type (WT) K562 cells or two single-cell K562 clones with control vector only (Fig. 1c). Second, we observed a significant upregulation of GAL3ST1 and FERT expression associated with the ectopic BORIS expression in BORIS-negative cell lines, HEK293T (human embryonic kidney) and MDA-MB-435 (melanoma cell line), respectively (Fig. 1d,e; Additional file 1: Fig. S1b-f). The activation of intronic promoters was also dependent on cell-specific context: GAL3ST1 intronic promoter was significantly activated in HEK293T cells ectopically expressing BORIS, but not in BORIS-expressing MDA-MB-435 cells. At the same time, the FERT promoter was significantly activated in MDA-MB-435, but not in HEK293T cells. The integration of ChIP-seq with RNA-seq data showed that the differential activation of the two testis-specific promoters depends on their co-occupancy by CTCF and BORIS proteins. Specifically, the absence of BORIS binding at the FERT promoter in HEK293T cells, likely due to comparatively lower BORIS occupancy (Additional file 1: Fig. S1d), suggests its lack of activation. Conversely, the GAL3ST1 promoter, which does not show CTCF binding in MDA-MB-435 cells (Fig. 1c; Additional file 1: Fig. S1f), indicates a similar absence of promoter activation. Therefore, the direct binding of BORIS to intronic regions concurrently bound by CTCF in FER and GAL3ST1 genes appears to be associated with transforming these CTCF sites into active internal promoters, thereby driving alternative cancer-testis-specific transcriptional start sites.

### BORIS binding to intronic and intergenic CTCF sites is associated with active transcription in K562 cells

**Fig. 2:**
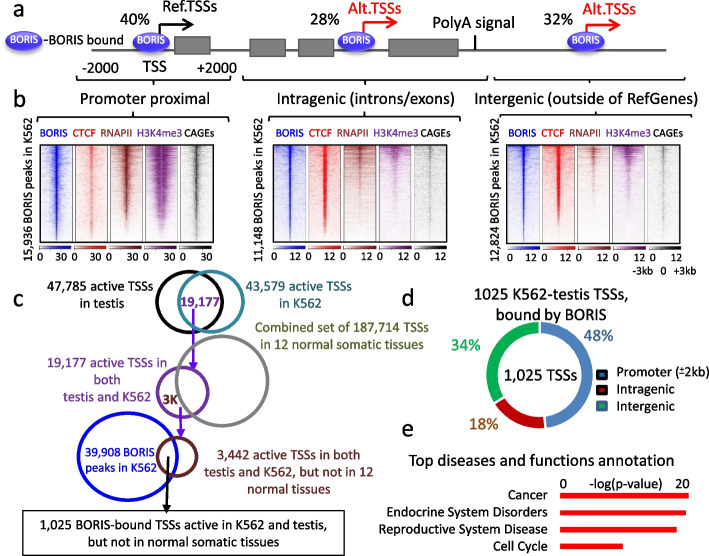
Intragenic and intergenic BORIS binding sites are associated with active transcription in K562 and male germ cells. **a** Schematic representation of ChIP-seq data illustrating the distribution of BORIS binding sites in K562 cells relative to a reference gene structure. Among the identified binding sites, 40, 28, and 32% are located around TSSs, inside introns or exons, and outside of RefSeqGenes, respectively. Canonical and alternative TSSs are denoted by black and red arrows, respectively, based on NGS data shown in panel **b**.** b** Heatmaps illustrating the enrichment profiles of BORIS, CTCF, RNAPII, H3K4me3 ChIP-seq signals, and CAGE-seq reads within a 6-kilobase (kb) window centered on BORIS binding sites in K562 cells. These heatmaps correspond to the genomic distribution pattern depicted in panel **a**. **c** Schematic representation of the strategy used to map TSSs associated with K562-testis-specific transcription driven from BORIS sites in K562 cells. **d** Genomic distribution of 1025 TSSs, active in both testis and K562 cells, relative to their genomic location with respect to RefSeqGenes. **e** Top diseases or function annotation for the 1025 TSSs identified using the strategy illustrated in panel **c**

To expand the analysis initially performed on GAL3ST1 and FERT genes to a genome-wide scope, we aimed to explore the potential role of BORIS binding sites in activating alternative transcriptional start sites in both male germline and cancer cells. Initially, we verified that the BORIS binding sites identified in K562 cells exhibited a significant overlap with markers indicative of active transcription (H3K4me3, CAGEs) in human male germ cells (Additional file 1: Fig. S2a). By examining the genomic distribution of BORIS occupancy identified through ChIP-seq in K562 cells, we observed that approximately 40% (15,936 out of 39,908 BORIS peaks mapped in K562) of these sites were located in promoter regions (within “ ± 2” kb from the TSSs) of RefSeq genes in hg19), while 28 and 32% (11,148 and 12,824, respectively) were located within intragenic and intergenic regions (Fig. 2a). Utilizing the enrichment signals of CAGEs, RNAPII, and H3K4me3 in K562 cells, we determined that epigenetic signatures associated with active transcription were present across all BORIS-occupied sites, irrespective of their genomic location in relation to the RefSeqGenes structure (Fig. 2b). The relatively lower enrichment of active transcription marks at BORIS sites located outside of reference TSSs (Fig. 2b) might be attributed to their lower reliance on chromatin interactions involving active enhancers, in contrast to BORIS sites residing at RefSeq TSSs (Additional file 1: Fig. S2b). Consequently, thousands of intragenic and intergenic BORIS binding sites in K562 cells have the potential to function as alternative promoters in cancer cells, mirroring the scenario observed with GAL3ST1 and FERT genes.

To assess cancer-testis transcription driven by BORIS binding in K562 cells, we established a strategy. We assumed that transcripts from alternative promoters should be associated with de novo active TSSs, marked by enrichment of corresponding CAGE-seq tag reads. Consequently, we overlapped 47,785 and 43,579 TSSs identified by CAGE-seq in human testes and K562 cells, respectively (Additional file 2: Table S1) (Fig. 2c). Out of these, 19,177 TSSs were found to be active in both cell types. Since the majority of these TSSs were also active in normal somatic cells, we excluded those active in 12 normal somatic tissues (187,714 TSSs, Additional file 2: Table S1) (Fantom5 data, [49]). That produced a list of 3442 TSSs that were transcribed in both human testes and K562 cells, but not in normal somatic tissues. Of these TSSs, 1025 (30%) overlapped with BORIS binding sites in K562 cells (Additional file 3: Table S2a), thus giving a set of potential targets for BORIS-driven transcription in both male germ and K562 cells. We categorized these TSSs into three groups based on their genomic location to RefSeqGenes structure: (1) Promoter regions (48.2%), 4 kb centered at RefSeqGenes TSSs; (2) Intragenic regions (18%), within introns and exons of RefSeqGenes located over 2 kb downstream of reference promoters; and (3) Intergenic regions (33.9%), situated at least 2 kb outside of RefSeqGenes (Fig. 2d, Additional file 3: Table S2). This approach was validated by the presence of intronic promoters of GAL3ST1 and FER genes among the recovered genes with intragenic TSSs (Additional file 3: Table S2).

Functional enrichment analysis of 790 RefSeqGenes corresponding to the 1025 TSSs, which were active in both testis and K562 cells, but not in 12 normal somatic tissues revealed a significant association of these genes with cancer, endocrine and reproductive system disorders, and cell cycle pathways (Fig. 2e). The highest expression of the 790 genes was detected in acute myeloid leukemia (AML) among 218 samples of 14 common tumor types from the global cancer map [50], which is correlated with the original source of gene selection from K562 cells (Additional file 1: Fig. S2c). Furthermore, 103 out of 790 genes were previously described as highly expressed during spermatogenesis [51], with 18 of them belonging to the cancer-testis antigen (CTA) group [52]. To analyze whether BORIS is directly involved in the activation of these CT genes in cancers, we compared their expression in wild-type to BORIS-depleted K562 cells. This revealed a significant downregulation of five CTAs: CTAG2, MAGEB1, PAGE4, SAGE1, MAGEA8 upon loss of BORIS occupancy at the respective promoter regions (Additional file 1: Fig. S2d-f). Moreover, the ectopic expression of BORIS in MDA-MB-435 cells resulted in the upregulation of 75 CT genes, 15 of them from a silent state, thus confirming the role of BORIS in the direct regulation of multiple CTAs (Additional file 1: Fig. S2g-i).

Using the approach illustrated in Fig. 2c, we identified at least 193 genes, which may express from alternative BORIS-bound intronic promoters in both cancer and male germ cells (Additional file 3: Table S2c), similar to GAL3ST1 and FERT. Some of the genes have already been reported to express alternative testis-specific transcripts and/or protein isoforms. For example, pyridoxal kinase PDXK/PKH is highly expressed in somatic tissues, but it is also expressed as an alternative isoform (PKH-T) during spermatogenesis [53]. This testis-specific isoform of the PKH gene is expressed from the intronic, BORIS-bound promoter in K562 cells (Additional file 3: Table S2b). However, most of the testis-specific promoters of the 193 genes have not been previously described. Among them, for example, is NOS3, nitric oxide synthase, which is highly expressed in a variety of human cancers and plays a key role in tumor progression [54]. Based on CAGE-seq analysis, the NOS3 gene is expressed as a shorter isoform in both male germ and K562 cells (Additional file 1: Fig. S3a). This isoform has the same TSS as the protein-coding isoform NOS3-006 (Ensembl.org) and is initiated from an intronic CTCF/BORIS binding site, which shows all epigenetic characteristics of actively transcribed TSS in K562 but not NHEK cells (Additional file 1: Fig. S3a). Another example, RNFT2 (RING finger transmembrane-domain containing protein 2), is expressed from two alternative BORIS-bound intronic promoters in both testes and K562 cells (Additional file 1: Fig. S3b), but not in BORIS-negative cells. Similar to NOS3 gene, RNTFT2 expression is associated with poor prognosis in cancer [55]. The full list of genes with intronic TSSs is presented in Additional file 3: Table S2c.

Besides alternative intragenic TSSs, there were also 347 intergenic BORIS-bound TSSs located outside the reference genes. A detailed interrogation of these TSSs revealed that some of them served as promoters for long noncoding RNAs (lncRNAs) (Additional file 1: Fig. S3c), which were not included in the list of RefSeqGenes. The NONCODE v6 database (http://www.noncode.org/) showed that the majority of these intergenic TSSs (70%, 242 out of 347) were indeed associated with unannotated long noncoding transcripts (Additional file 3: Table S2d). A review of the remaining 105 intergenic TSSs revealed that 25 of them were associated with retroposed genes and the rest belonged to transcripts that are neither in the list of RefSeqGenes nor in NONCODE databases (Additional file 1: Fig. S3d,e, Additional file 3: Table S2d). Taking these results together, we conclude that BORIS binding sites, independent of their genomic positions, are associated with transcription of CT genes, including known CTAs, long noncoding RNAs, unannotated transcripts, and retroposed genes in both cancer and male germ cells.

### Ectopic expression of BORIS leads to cellular transformation, accompanied by pervasive upregulation of transcription

**Fig. 3:**
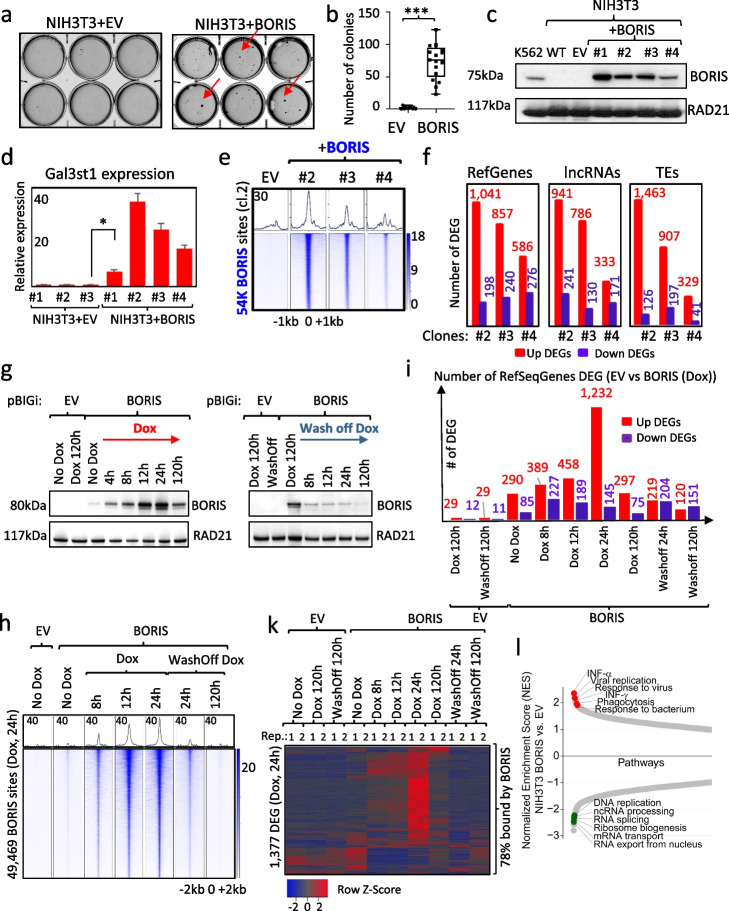
Ectopic BORIS expression transforms NIH3T3 cells and deregulates gene transcription. **a** Representative images of soft agar colonies. **b** Quantification of the number of soft agar colonies. Two-tailed Student’s *t* test (***—*p* < 0.0001). **c** Western blot analysis of BORIS expression in nuclear extracts isolated from four single-cell clones of NIH3T3 cells, recovered from a soft agar. K562—positive control. Parental (WT) and EV-expressing NIH3T3 cells were used as a negative control for BORIS expression. RAD21 Abs were used as a loading control. **d** RT-qPCR results displaying the relative expression levels of testis-specific Gal3St1 in NIH3T3 + EV, including EV-total culture (#1), and single-cell clones with EV (#2, #3), compared to NIH3T3 + BORIS clones recovered from soft agar (#1, 2, 3, 4). Two-tailed Student’s *t* test ( *—*p* < 0.005). Error bars indicate mean ± SD (*n* = 3). **e** Heatmap depicting BORIS occupancy in three BORIS-expressing clones (#2,3,4) compared to EV. **f** Quantification of the number of upregulated (red) and downregulated (blue) RefSeqGenes, lncRNAs, and TEs in three BORIS-expressing clones (#2,3,4), compared to EV, with log_2_ fold change > 1.3 and *p*-value (padj) < 0.05. **g–k** NIH3T3 cells were stably transfected with either the doxycycline-inducible empty vector (pBIGi-EV) or BORIS (pBIGi-BORIS). The cells were cultured under different conditions: in the absence of doxycycline (No Dox), induced with doxycycline (Dox) for a specified number of hours, and doxycycline removal (Wash Off) for an indicated number of hours. **g** Western blot confirms BORIS activation by dox. **h** Heatmap of BORIS occupancy in doxycycline-treated cells compared to EV and cells without doxycycline treatment. **i** Quantification of upregulated (red) and downregulated (blue) RefSeqGenes in doxycycline-induced EV or BORIS cells compared to EV (no dox) or EV (dox 12 h), respectively, with log_2_ > 1.3, *p*-value < 0.001. **k** The heatmap depicts the expression profile of 1377 genes (RNA-seq data, 2 replicates for each condition), which were significantly deregulated in BORIS-expressing cells treated with dox for 24 h, comparing the expression in EV versus BORIS-induced cells. **l** GSEA of RNA-seq data for BORIS-expressing cells isolated from soft agar, compared to EV

While BORIS expression in the germline is only a part of a testis-specific pathway, which leads to gamete differentiation [43, 46, 47], its ectopic expression in somatic and aberrant activation in cancer cells is overwhelming enough to alter the regulation of thousands of genes [18, 19, 22, 36]. However, it is unclear whether BORIS activation can actually lead to the malignant transformation of normal somatic cells. To address this, we transfected BORIS-negative, normal cell lines (NHDF, MCF10A, HMEC, NHEK, NIH3T3) with either a CpG-free BORIS-expressing vector, or with a control plasmid (empty vector, EV), expressing beta-galactosidase. The newly transfected cells were plated in soft agar to grow without any antibiotic pressure. Only NIH3T3 cells, which stably express BORIS (NIH3T3 + BORIS), were able to produce multiple colonies in soft agar, thus acquiring the transformed phenotype (Fig. 3a,b). Several single-cell-derived NIH3T3 clones were extracted from soft agar and propagated under regular cell culture conditions, now under antibiotic pressure. The presence of the BORIS protein in all single-cell clones recovered from the soft agar was confirmed by Western blotting using specific antibodies (Fig. 3c), indicating that BORIS expression was necessary for cell transformation. After transitioning NIH3T3 + BORIS cells from agar to regular culture conditions, the cells became phenotypically similar to NIH3T3 + EV cells, which were obtained from a mixed clone culture isolated through antibiotic selection (Additional file 1: Fig.S4a). However, the transformed phenotype could still be observed when cells were grown in serum-free media (Additional file 1: Fig. S4b). In this environment, BORIS-expressing cells formed multiple distinct foci, which could survive for up to 10 days without serum, unlike EV cells, which could not (Additional file 1: Fig. S4b).

To investigate whether BORIS activates cancer-testis-specific transcription in somatic cells, we analyzed Gal3st1 expression in NIH3T3 cells expressing either EV or BORIS. We observed a robust induction of Gal3st1 expression in all four BORIS-expressing clones, with the highest activation in clones #2, #3, and #4 (Fig. 3d). These three clones were chosen for further genome-wide analyses using ChIP-seq and RNA-seq to evaluate the transcriptomic and epigenetic changes following ectopic BORIS expression. ChIP-seq analysis demonstrated the strongest genome-wide BORIS binding in the clone#2 compared to the other clones (Fig. 3e), which was in agreement with the highest BORIS expression observed in qPCR analysis (Additional file 1: Fig. S5a). Concurrently, the RNA-seq data revealed that the ectopic expression of BORIS induced the most profound transcriptomic changes in the clone#2, resulting in the upregulation of 1041 RefSeqGenes and the downregulation of 198 RefSeqGenes, respectively (log_2_ fold change > 1.3, *p*-value (padj < 0.05) (Fig. 3f, Additional file 1: Fig. S5b.). Similar transcriptional changes were observed in the clone #3, showing a significant overlap of upregulated genes among the clones (504 genes, Fisher test, *p* < 0.00001) (Fig. 3f, Additional file 1: Fig. S5c,e). Conversely, fewer genes were deregulated in the clone#4 (Fig. 3f, Additional file 1: Fig. S5d), which paralleled its lower overall BORIS expression and binding. Therefore, the clone#2, exhibiting the most profound BORIS occupancy and transcriptional changes (Fig. 3e,f), was selected for a more comprehensive genome-wide analysis.

The list of RefSeqGenes includes only well-annotated coding and noncoding transcripts, while our analysis of TSSs in K562 cells revealed that BORIS binding was also associated with the expression of unannotated long noncoding transcripts (Additional file 1: Fig. S3, Additional file 3: Table S2d). Given this, we conducted an additional analysis of noncoding RNAs, which are generally not included in the list of RefSeqGenes. RNA-seq analysis of NIH3T3 cells expressing BORIS showed an upregulation of 941 and downregulation of 241 nonannotated lncRNAs (Noncode, v6), as well as an upregulation of 1463 and downregulation of 126 transposable elements (TEs) expression in the clone#2, compared to EV, respectively (log_2_ fold change > 1.3, *p*-value (padj) < 0.05) (Fig. 3f, Additional file 1: Fig. S5b). Similar changes of noncoding transcripts were detected for the clones #3 and #4, proportional to the numbers of differently expressed RefSeqGenes (DEG) for the same clones (Fig. 3f, Additional file 1: Fig. S5c,d). Overall, the number of upregulated transcripts was several times greater than the number of downregulated transcripts in all three clones (Fig. 3f). This suggests that BORIS binding is more strongly associated with the activation of transcription than the repression. A manual curation of the RNA-seq data revealed that there are still some transcripts deregulated by BORIS expression, but they are not included in the list of either RefSeqGenes or lncRNAs (Additional file 1: Fig. S5f). Therefore, we compiled all transcripts expressed in NIH3T3 + BORIS (clone#2) and NIH3T3 + EV cells, but not included in any list of reference coding or noncoding transcripts. Thus, we identified 642 novel transcripts (Additional file 4: Table S3), out of which 125 were either significantly upregulated (118) or downregulated (7) in NIH3T3 + BORIS cells compared to NIH3T3 + EV cells (Additional file 1: Fig. S5g). Some of these novel transcripts were upregulated from a previously silent state (Additional file 1: Fig. S5f) and may represent cases of spurious transcription activated by BORIS binding. Additionally, to explore whether ectopic BORIS expression induces similar transcriptomic changes in human cell lines, we examined gene expression in MDA-MB-435 and MM057 human melanoma cell lines, both of which stably expressed either EV or BORIS from vector. Similar to NIH3T3 + BORIS cells, ectopic BORIS expression in both human cell lines led to the deregulation of hundreds of coding and lncRNA transcripts, as well as retroposed genes and transposable elements (Additional file 1: Fig. S6a,b). The combination of RNA-seq with ChIP-seq data revealed that the majority of both upregulated (78%) and downregulated (57%) RefSeqGenes in NIH3T3 + BORIS (clone#2) versus NIH3T3 + EV cells exhibited BORIS binding within the deregulated loci, suggesting a direct involvement of BORIS in the corresponding transcriptional changes (Additional file 1: Fig. S5b). Similar results were observed for the clones #3 and #4 of NIH3T3 + BORIS cells (Additional file 1: Fig. S5c,d), as well as in human MDA-MD-435 + BORIS cells (Additional file 1: Fig. S6a). Also, in genes directly bound by BORIS, the binding tended to localize upstream of TSSs for the upregulated genes and downstream of TSSs for downregulated ones (Additional file 1: Fig. S6c). Taken together, this affirms the transcription factor role of BORIS in governing gene expression.

Furthermore, to demonstrate the direct impact of BORIS binding on gene expression, we employed a doxycycline (dox)-inducible system (pBIGi vector) [56] to either transiently overexpress or repress BORIS expression in NIH3T3 cells, in comparison to the empty vector (EV). First, we conducted a time course of BORIS activation with dox treatment for 4, 8, 12, 24, and 120 h (Fig. 3g–k). Interestingly, the highest BORIS activation was detected after 24 h of dox treatment, decreasing after a total of 5 days (120 h) of treatment (Fig. 3g). The decline in BORIS levels may be attributed to the potential toxicity associated with BORIS expression, underscoring the necessity for BORIS protein levels to be tightly regulated for cells to survive and/or propagate [25, 57, 58]. Following BORIS activation by dox, hundreds of genes were significantly deregulated, with the highest number of those coinciding with the peak of BORIS activation and genome-wide occupancy, compared to control cells with vector only (Fig. 3g–k, Additional file 1: Fig. S7a). Genes that showed changes in expression at 8 h substantially overlapped with those deregulated at 12 and 24 h, albeit with a greater number of genes deregulated at 24 h (Fig. 3k, Additional file 1: Fig. S7b). The majority of deregulated RefSeq genes (78%) exhibited a direct BORIS binding in the vicinity of a gene (Fig. 3k). Furthermore, washing off doxycycline after the induction of BORIS, accompanied by the loss of BORIS binding, restored the parental NIH3T3 gene expression signature (Fig. 3h,k). The coincidence of gene expression changes with the ectopic expression of BORIS in transient (dox-induced) and stable (soft agar colonies) conditions revealed that there were 354 genes strongly activated by direct BORIS binding (Additional file 1: Fig. S7c). In summary, these data indicate that BORIS binding directly contributes to gene expression deregulation, as evidenced by both dose-dependent and time-dependent responses to BORIS expression.

While analyzing gene expression in BORIS-expressing clones, isolated from soft agar, we observed that the deregulation of noncoding RNAs (lncRNAs and TEs) was proportional to the deregulation of RefSeq genes (Fig. 3f). Namely, there was a trend that the higher number of upregulated RefSeq genes was accompanied by a higher number of upregulated noncoding RNAs, compared to a lower number of downregulated genes (Fig. 3f). Correspondingly, in the time course of dox-inducible cells, we observed a similar tendency: the maximal activation of lncRNAs and TEs coincided with the highest number of deregulated RefSeq genes at 24 h of dox treatment (Fig. 3i, Additional file 1: Fig. S7d). Notably, both lncRNAs and TEs exhibited a less prominent association with BORIS binding sites (Additional file 1: Figs. S5b-d and S6a), suggesting that their deregulation may be mediated by some intermediary factors conceivably activated by BORIS expression. Incidentally, genomic analysis of deregulated noncoding RNAs reveals a significantly high proportion of them (Fisher test, *p* < 0.00001) located close by or within the body of long transcripts directly bound by BORIS (Additional file 1: Fig. S8a,b). Specifically, 30.6 and 13.5% of upregulated lncRNAs and TEs, respectively, overlap with upregulated RefSeq genes in NIH3T3 + BORIS (clone#2). Similarly, 20.6 and 11.9% of downregulated lncRNAs and TEs, respectively, overlap with downregulated RefSeq genes in the same clone. Importantly, there is no genomic overlap observed between deregulated noncoding RNAs and RefSeq genes when they are differentially expressed in opposite directions. These findings suggest that the upregulation or downregulation of some noncoding RNAs is likely linked to the upregulation or downregulation, respectively, of the hosting coding or noncoding long transcripts, in turn directly controlled by BORIS expression (Additional file 1: Fig. S8a,b). Given that TEs are typically maintained in a silent state through various mechanisms, including CpG methylation and the heterochromatinization [59], we conducted mapping of active (H3K36me3) and repressive (H3K27me3) histone marks in NIH3T3 + BORIS (clone#2) and NIH3T3 + EV cells. In line with the observed expression changes, we observed a gain of active histone modification (H3K36me3) at upregulated TEs and a loss of this modification at downregulated TEs (Additional file 1: Fig. S8a-c). In contrast, no changes in repressive histone marks were observed (Additional file 1: Fig. S8c). In summary, we suggest that one of the mechanisms leading to the deregulation of TEs involves direct binding of BORIS to promoter regions of RefSeq or lncRNAs genes. This binding results in the activation or downregulation of the transcription of long transcripts. Consequently, this process can initiate epigenetic changes at the locus, potentially causing the deregulation of TEs embedded within these long transcripts or situated in close proximity.

The activation of TEs likely triggers their transposition, leading to the upregulation of intracellular antiviral responses, such as the interferon alpha and interferon gamma pathways in NIH3T3 + BORIS cells (soft-agar clones), as compared to NIH3T3 + EV (Fig. 3l). The activation of inflammatory pathways subsequent to ectopic BORIS expression was initially identified through Gene Set Enrichment Analysis (GSEA). This finding was subsequently confirmed via Western blotting, which revealed increased expression levels of key markers associated with the upregulated pathways (Fig. 3l, Additional file 1: S8d, Additional file 5: Table S4). Analogously, activation of antiviral responses through the Aim2 inflammasome-based pathway was also described upon knockout of G9a/Ehmt2 (H3K9 methyltransferase), which is responsible for keeping TEs repressed [60]. Aim2 protein is involved in the innate antiviral response by recognizing cytosolic double-stranded DNA [61]. Similarly to Avgustinova et al. [60], we detected an upregulation of Aim2 expression in all three BORIS-expressing clones, derived from soft agar (Additional file 1: Fig. S8e). Moreover, the highest activation of long-interspersed nuclear elements-1 (LINE1) ORF1p in the clone#4 of NIH3T3 + BORIS coincided with the highest activation of Aim-2 expression, suggesting an interdependence between the two events (Additional file 1: Fig. S8f). It is worth to emphasize that the activation of the autonomous LINE1 is particularly significant, as LINE1 has the capability to mobilize other transposable elements (TEs) [12] and induce DNA double-strand breaks [62], connecting the activation of LINE1 with the upregulation of Aim2 observed in the clone#4. Comparable to soft-agar-derived NIH3T3 + BORIS cells, the main common pathways upregulated upon ectopic BORIS expression in dox-inducible NIH3T3 and in human cells (MDA-MB-435 and MM057) were related to inflammation and antiviral responses, while downregulated pathways were associated with cell cycle progression (Additional file 1: Fig. S8g, Additional file 5: Table S4). To summarize, we have demonstrated that BORIS binding activates the transcription of both protein-coding and long noncoding transcripts. This transcriptional activation likely leads to the opening of chromatin throughout the gene body, resulting in the upregulation of TE expression and the activation of inflammation pathways.

### BORIS initiates alternative promoter usage by epigenetically reprogramming clustered CTCF binding sites into active transcription start sites

**Fig. 4:**
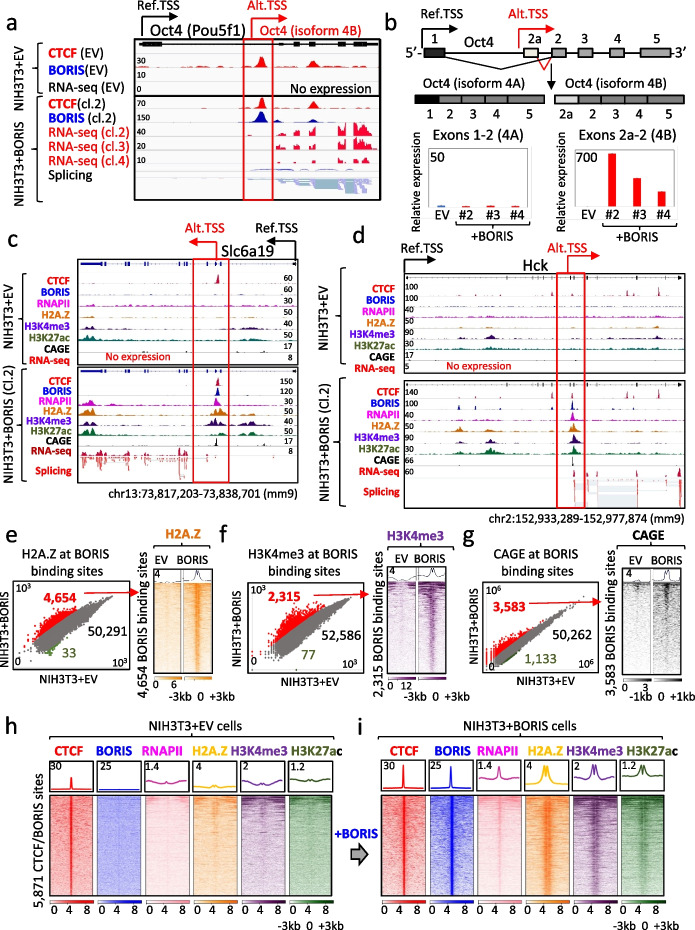
BORIS binding epigenetically reprograms transcriptionally inert CTCF binding sites into active promoters. **a** Genome browser view of ChIP-seq data and RNA-seq data for NIH3T3 + EV versus NIH3T3 + BORIS (clone#2, soft-agar derived) cells. CTCF (red) and BORIS (blue) co-occupancy in NIH3T3 + BORIS cells leads to the activation of Oct4 (Pou5f1) gene expression from an alternative intronic promoter (highlighted by red arrow and open box) in all three BORIS-expressing clones (RNA-seq data), compared to no expression in NIH3T3 + EV cells. **b** Upper panel shows the exon/intron structure of Pou5f1. Black and red arrows indicate reference and alternative promoters of Pou5f1, respectively. Black and white boxes represent coding exons specific to isoforms 4A and 4B, respectively; grey boxes represent exons shared by both isoforms. Lower panel compares the relative expression of Pou5f1 isoforms in three BORIS-expressing soft-agar-derived NIH3T3 clones to NIH3T3 + EV cells, revealing upregulation of only isoform 4B upon BORIS expression. **c,d** Genome browser view showing that CTCF and BORIS co-binding in NIH3T3 + BORIS (clone#2) cells is associated with the enrichment of active histones/marks (H2A.Z, H3K4me3, H3K27ac), RNAPII, CAGE-seq reads and the activation of alternative transcription (RNA-seq) from *Slc6a19* (**c**) and *Hck* (**d**) genes. The alternative promoters are silent under CTCF-only occupancy in NIH3T3 + EV cells. **e–g** Left panel shows scatter plots of normalized read counts (log_10_) for H2A.Z **e**, H3K4me3 **f**, and CAGE **g** occupancy at BORIS-bound sites in NIH3T3 + BORIS (clone#2) cells compared to the same genomic sites in NIH3T3 + EV cells. BORIS sites with gain or loss of active histone marks or CAGEs are highlighted by red or green colors, respectively. The right panel displays a heatmap of H2A.Z **e**, H3K4me3 **f**, and CAGE **g** occupancy at BORIS-bound sites that gained active marks in NIH3T3 + BORIS (clone#2) cells compared to NIH3T3 + EV cells. Red arrows connect the left and right panels, indicating the number of gained active histone marks at BORIS-bound sites. **h,i** Heatmap representation of CTCF (red), BORIS (blue), RNAPII (pink), H2A.Z (yellow), H3K4me3 (purple), and H3K27ac (green) occupancy in NIH3T3 + EV cells (**h**) compared to NIH3T3 + BORIS (clone#2) cells (**i**) at the 5871 CTCF/BORIS binding sites, which gained occupancy of at least one active mark of transcription

While the ectopic expression of BORIS leads to the activation of numerous genes, including long noncoding RNAs (lncRNAs) and transposable elements (TEs) in NIH3T3, MDA-MB-435, and MM057 cells (Fig. 3, Additional file 1: Fig. S5-S8), the precise molecular mechanisms underlying this activation remain unknown. To address this, we examined the genes that underwent significant activation from a silent state in soft-agar-derived NIH3T3 + BORIS cells, in comparison to NIH3T3 + EV cells. One such gene is Oct4/Pou5f1, a well-studied factor associated with the pluripotency of embryonic stem (ES) cells. Through the integration of ChIP-seq and RNA-seq data for NIH3T3 cells before and after ectopic BORIS expression, we confirmed that BORIS binding to an already bound by CTCF genomic region within the first intron of the Oct4 gene triggered activation of the alternative intronic promoter (Fig. 4a, Additional file 1: Fig. S9a). Aligning our findings with previously published studies on Oct4 isoforms, we established that BORIS binding to this intronic sequence specifically activates the Oct4b isoform [63]. It is well-documented that, unlike the extensively studied Oct4a isoform (commonly referred to as Oct4), Oct4b has a distinct N-terminal transactivation domain. Oct4b is unable to maintain the stemness properties of ES cells; however, it is expressed during the earliest stages of embryogenesis [63] and functions as a stress response factor [64]. To evaluate BORIS’s role in Oct4b activation, we designed a set of primers to differentiate the expression of Oct4a (exon 1–2) from Oct4b (exon 2a-2) using qRT-PCR. As depicted in Fig. 4b, only Oct4b demonstrated activation in NIH3T3 cells across all three BORIS-expressing clones, while no activation was observed in EV cells. Furthermore, we verified a direct, dose-dependent role of BORIS in activating Oct4b through a time course doxycycline induction of BORIS expression by qPCR (Additional file 1: Fig. S9b), ChIP-seq, and RNA-seq (Additional file 1: Fig. S9c). Consequently, the mechanism underlying Oct4b activation in this model likely parallels that of GAL3ST1 and FERT regulation by BORIS (Fig. 1): the co-occupation of intronic regions by both CTCF and BORIS proteins results in the activation of alternative transcriptional start sites.

Upon further examination of genes that were highly activated from a previously silent state, it became evident that many of them were expressed from alternative promoters bound by both CTCF and BORIS in NIH3T3 + BORIS cells, derived from soft agar. This stands in contrast to NIH3T3 + EV cells, where the same genes remained silent, relying solely on CTCF binding. For instance, Slc6a19 (Solute Carrier Family 6 Member 19) and Hck (HCK Proto-Oncogene, Src Family Tyrosine Kinase) were activated from an intronic CTCF binding site, which was bound by both CTCF and BORIS in NIH3T3 + BORIS cells (Fig. 4c,d). These genes, however, remained silent under CTCF occupancy alone in control cells. Moreover, some of the highly activated genes, such as Ly6h (Lymphocyte Antigen 6 Family Member H), were upregulated from CTCF/BORIS binding sites located 44 kb upstream of the known gene’s TSS, effectively bypassing another coding gene, Gpihbp1 (Additional file 1: Fig. S10a). This suggests a clear tendency for some CTCF binding sites located within intragenic or intergenic regions to undergo transformation into active TSSs following BORIS recruitment.

To investigate the aforementioned intragenic and intergenic CTCF sites reprogramming further, we conducted ChIP-seq mapping for RNA Polymerase II (RNAPII) and active epigenetic marks (H2A.Z, H3K4me3, H3K27ac) occupancies in both NIH3T3 + EV and NIH3T3 + BORIS cells (clone#2). In order to distinguish between the activation of canonical and alternative promoters, we complemented our analysis with CAGE-seq. The results, as represented by the five examples in Fig. 4a–d and Additional file 1: Fig. S10a,b strongly indicate that the co-binding of BORIS with CTCF in NIH3T3 + BORIS cells initiated an epigenetic reprogramming of chromatin around CTCF-bound sites, coinciding with BORIS recruitment. After conducting sequence analysis on all five illustrative examples of CTCF sites transformed by BORIS binding into active transcription start sites, it becomes apparent that these sequences contain clustered CTCF motifs positioned beneath the CTCF peaks (Additional file 1: Fig. S10c). This places them into the class of clustered CTCF sites, or 2xCTSes, a classification previously detailed in [22]. The chromatin remodeling observed at the clustered CTCF sites is evidenced by the recruitment of RNAPII, the enrichment of CAGEs, and the presence of active histone marks including H3K27ac, H2A.Z, and H3K4me3, ultimately leading to a robust activation of transcription as confirmed by RNA-seq. Overall, we observed a synchronized appearance of all examined marks associated with active transcription at BORIS binding sites in NIH3T3 + BORIS (clone#2) cells when compared to NIH3T3 + EV (Fig. 4e–i, Additional file 1: Fig. S11a). Furthermore, we identified at least 4654 BORIS binding sites that acquired H2A.Z occupancy de novo in NIH3T3 + BORIS (clone#2) cells, exhibiting more than a fourfold increase compared to NIH3T3 + EV cells (Fig. 4e). Similarly, 4479 new H2A.Z ChIP-seq peaks detected in NIH3T3 + BORIS (clone#2) cells, but not in NIH3T3 + EV, displayed a strong association with BORIS binding (Additional file 1: Fig. S11b), along with a noticeable upregulation of gene transcription (Additional file 1: Fig. S11c). The same observation regarding the CTCF and BORIS-bound sites in NIH3T3 + BORIS (clone#2) cells, in contrast to CTCF-bound sites in NIH3T3 + EV cells, holds true for the active promoter histone mark, H3K4me3 (Fig. 4f, Additional file 1: Fig. S12a), the active enhancer histone mark, H3K27ac (Additional file 1: Fig. S12b,e), RNAPII occupancy (Additional file 1: Fig. S12c,f), and CAGEs enrichment (Fig. 4g, Additional file 1: Fig. S12d). Moreover, it is worth noting that the ectopically expressed BORIS protein, when bound to an endogenous BORIS promoter in NIH3T3 cells, evidently activated its own transcription (Additional file 1: Fig. S10b), a phenomenon reminiscent of other self-regulating reprogramming factors such as Oct4, Sox2, and Nanog [65].

Next, we sought to determine how many genomic regions changed their epigenetic status upon BORIS binding in NIH3T3 + BORIS (clone#2) cells, compared to the same regions in EV cells. We counted a total of 5871 BORIS binding sites, which were epigenetically altered in NIH3T3 + BORIS cells (clone#2), based on the gain of at least one epigenetic mark of active transcription. Figure 4h shows that these sites are bound only by CTCF in NIH3T3 + EV cells and depleted of any marks of active transcription. However, upon BORIS co-binding, these CTCF sites were epigenetically reprogrammed into active promoters, as documented by the enrichment of RNAPII, H2A.Z, H3K4me3, and H3K27ac (Fig. 4i). Similar enrichment of active histone marks was detected at the 5871 BORIS sites in the NIH3T3 + BORIS clones #3 and #4, albeit at a lower level in accordance with lower BORIS binding in these clones (Additional file 1: Fig. S13a-c). Moreover, CTCF occupancy at these sites was dramatically increased in coordination with BORIS co-binding (Fig. 4h,i), suggesting, in line with our prior studies [22, 43], that CTCF heterodimerization with BORIS may lead to more stable DNA binding compared to CTCF homodimerization by itself. To validate whether the reprogrammed set of CTCF binding sites belongs to the clustered CTCF sites class, we conducted a ChIP-Re-ChIP assay and performed motif analysis of CTCF and BORIS occupancy in NIH3T3 + BORIS (clone#2) cells (Additional file 1: Fig. S13d-f). Out of the 5871 sites examined, 88% overlapped with both CTCF and BORIS co-occupied sites as per the sequential chromatin immunoprecipitations, and 45% displayed the occurrence of at least two equal CTCF motifs under ChIP-seq peaks with a *p*-value less than 0.0001 (Additional file 1: Fig. S13d-f). The genomic distribution of these 5871 CTCF/BORIS sites, relative to RefSeq gene positions, is such that the majority of sites are located either outside of genes (47%) or inside introns/exons (41%), while only a minority (12%) of these sites reside in annotated RefSeq gene promoters (Additional file 1: Fig. S13g). Comparison between the BORIS binding sites and CAGE-seq data revealed that 99.9% (5868) of them were associated with CAGEs and 32% (1891) were upregulated in more than two times in NIH3T3 + BORIS cells (clone#2), compared to EV. As the normal expression pattern of BORIS is defined by spermatogenesis, it is noteworthy that 582 TSS regions active during spermatogenesis were part of these 5871 sites, reprogrammed by BORIS in NIH3T3 cells (Additional file 1: Fig. S13h).

Taken together, these results suggest that BORIS can epigenetically reprogram transcriptionally inert intragenic and intergenic CTCF binding sites, converting them into active alternative promoters. Thus, the aberrant activation of BORIS in multiple cancers may explain the activation of some testis-specific and alternative promoters frequently described as signatures of different cancers [10, 11].

### BORIS recruitment of chromatin remodeling factor SRCAP initiates an increase of H2A.Z histone occupancy at CTCF/BORIS binding sites

**Fig. 5:**
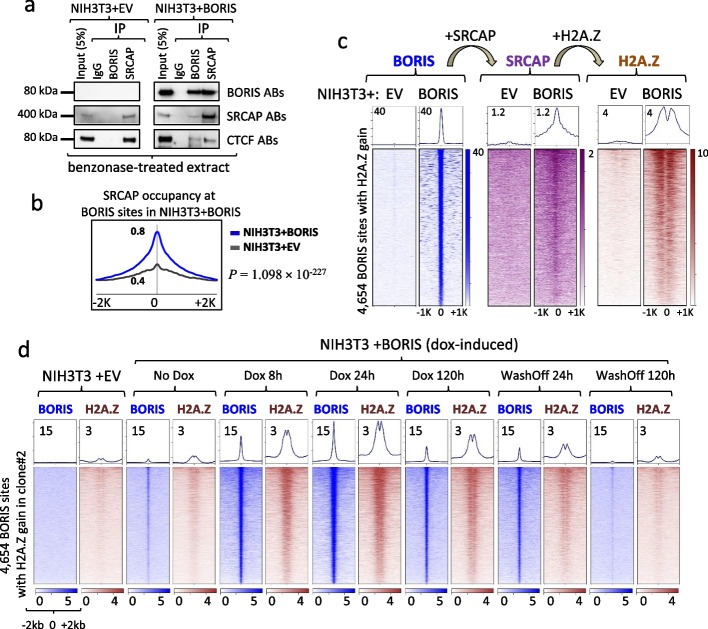
BORIS recruits SRCAP, which drives H2A.Z occupancy at its target sites. **a** Co-IP: BORIS directly interacts with SRCAP in NIH3T3 + BORIS (clone#2) cells. Benzonase-treated nuclear cell extracts were immunoprecipitated (IP) with either mouse IgG, anti-BORIS Abs, or anti-SRCAP Abs. Western blot analysis with indicated antibodies confirms the interaction. **b** Average plot of SRCAP occupancy (ChIP-seq data) at BORIS-bound sites in NIH3T3 + BORIS (clone#2) cells compared to NIH3T3 + EV cells. The *p-*value is calculated by Kolmogorov–Smirnov test. **c** Heatmap displaying BORIS (left panel, blue), SRCAP (middle panel, purple), and H2A.Z (right panel, brown) occupancy at the 4654 BORIS-bound sites in NIH3T3 + BORIS (clone#2) cells compared to NIH3T3 + EV cells. Arrows atop the heatmaps indicate the proposed sequence of events: BORIS binding to chromatin leads to SRCAP recruitment, resulting in the gain of H2A.Z occupancy around BORIS sites. **d** Heatmap displaying the correlation between H2A.Z occupancy and BORIS binding in both doxycycline-inducible empty vector (EV) and BORIS-expressing cells. The cells were subjected to treatment with or without doxycycline for a specified duration of time, as indicated

Based on the prior data, we investigated what mechanisms may contribute to the epigenetic reprogramming of CTCF binding sites by BORIS. Upon further analysis of our ChIP-seq data, we have identified that the most pronounced epigenetic change observed with ectopic BORIS expression is the substantial increase in H2A.Z histone occupancy at BORIS binding sites (Fig. 4e, Additional file 1: Fig. S11b). The H2A.Z histone, which plays an essential role in the initiation and regulation of transcription [66], is known to be incorporated into nucleosomes by two homologous proteins, SRCAP (SNF-2-related CREBBP activator protein) and p400 [66–68]. Incidentally, the SRCAP protein was also identified as a BORIS-interacting partner in a yeast two-hybrid system [69]. To investigate BORIS interaction with SRCAP in our own cell model, we first performed co-immunoprecipitation (co-IP) and reverse co-IP of the two proteins with both anti-SRCAP and anti-BORIS antibodies. Indeed, through both anti-BORIS and anti-SRCAP pull downs, our co-IP demonstrated a physical interaction between BORIS and SRCAP in NIH3T3 + BORIS (clone#2) cells. This interaction was observed both in the absence (Additional file 1: Fig. S14a) and presence of benzonase treatment of nuclear extracts (Fig. 5a). Additionally, staining of co-immunoprecipitates with anti-CTCF antibodies revealed that CTCF also interacts with SRCAP, albeit to a much lesser extent than with BORIS (Fig. 5a, Additional file 1: Fig. S14a). Similar co-IP with p400 antibodies showed no direct interaction between BORIS and p400 (Additional file 1: Fig. S14b). Furthermore, SRCAP ChIP-seq in NIH3T3 + EV and NIH3T3 + BORIS showed a significant (Kolmogorov–Smirnov test, *p* = 1.098 × 10^−227^) increase of SRCAP occupancy at BORIS binding sites in BORIS-positive cells (clone#2), compared to NIH3T3 + EV cells, consistent with the overall gain of H2A.Z occupancy at the same binding sites (Fig. 5b, Additional file 1: Fig. S14c). The analysis of 4654 H2A.Z sites induced by BORIS binding confirmed a significant enrichment of SRCAP at these sites in NIH3T3 + BORIS (clone#2) cells, while no such enrichment was observed in NIH3T3 + EV cells (Fig. 5c). The dox-inducible system also demonstrated that the gain of H2A.Z occupancy follows dox-induced BORIS binding in NIH3T3 cells (Fig. 5d). Additionally, the treatment of dox-inducible NIH3T3 + BORIS cells with anti-H2A.Z siRNA prior to dox-induction of BORIS expression showed that H2A.Z downregulation dramatically affected the activation of BORIS targets relatively to cells treated with control siRNA (Additional file 1: Fig. S14d-g). Furthermore, the time course of dox-inducible cells revealed that the increase in H2A.Z occupancy at BORIS sites precedes a gain of H3K4me3 (Additional file 1: Fig. S14h), underscoring the importance of H2A.Z histone recruitment for BORIS activated transcription. The *K*-means clustering of H2A.Z occupancy at BORIS binding sites showed that H2A.Z histone was either introduced into both nucleosomes flanking BORIS sites or only on one side (Additional file 1: Fig. S14i). Thus, BORIS can remodel chromatin around CTCF binding sites by recruiting SRCAP protein, which, in turn, incorporates H2A.Z histones into surrounding nucleosomes, facilitating activation of transcription from these sites.

To investigate whether the ectopic BORIS expression could cause a gain of H2A.Z occupancy in other cell types, we analyzed H2A.Z occupancy in several BORIS-negative cell systems prior to and after ectopic expression of BORIS (Additional file 1: Fig. S14k). We recently described a gain of ectopically expressed BORIS occupancy in mutant CH12 cells (CTCF with only 8 ZFs), compared to WT CH12 cells [34]. In this context, the rise in BORIS occupancy, when compared to wild-type CH12 cells, was associated with a significant increase in H2A.Z signal (Additional file 1: Fig. S14k, Kolmogorov–Smirnov test, *p* = 6.625 × 10^−103^). Furthermore, both stable expression of BORIS in MDA-MB-435 cells and transient expression of BORIS in MCF7 cells led to a significant increase in H2A.Z occupancy at BORIS binding sites (Additional file 1: Fig. S14k, Kolmogorov–Smirnov test, *p* = 6.644 × 10^−41^ (MDA-MB-435 + BORIS cells) and *p* = 7.989 × 10^−30^ (MCF7 + BORIS cells)). This indicates a strong correlation between the gain of H2A.Z occupancy and BORIS expression in different cell systems, providing further evidence that BORIS binding leads to epigenetic modifications favoring a more open and active chromatin state.

### BORIS induces chromatin relaxation, leading to an increase in CTCF occupancy

**Fig. 6:**
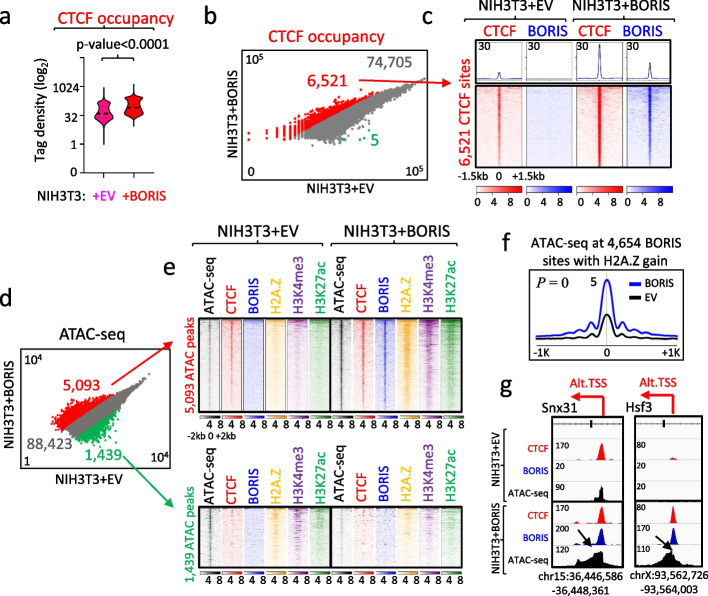
BORIS expression is associated with a significant increase in CTCF occupancy and a more open chromatin state around CTCF sites. **a** CTCF occupancy mapped in NIH3T3 + EV versus NIH3T3 + BORIS (clone#2) cells, depicted through a violin plot. **b** Scatter plot shows normalized read counts (log_10_) for CTCF occupancy at the combined set of CTCF binding sites in NIH3T3 + BORIS (clone#2) cells compared to NIH3T3 + EV cells. CTCF sites with increased or decreased occupancy by more than threefold are highlighted in red and green, respectively. **c** Heatmap illustrating CTCF (red) and BORIS (blue) occupancy at the 6521 CTCF sites from panel **b** (connected by red arrow). **d** Scatter plot of normalized read counts (log_10_) for ATAC-seq tag density at the combined set of ATAC-seq genomic sites mapped in both NIH3T3 + EV and NIH3T3 + BORIS (clone#2) cells. Genomic sites with increased or decreased occupancy by more than twofold are highlighted in red and green, respectively. **e** Heatmaps combining ATAC-seq (black) accessibility with CTCF, BORIS, H2A.Z, H3K4me3, and H3K27ac occupancy at genomic regions from panel **d** (connected by red and green arrows). Upper and lower panels display data for increased and decreased ATAC-seq sites, respectively. **f** Average plot of chromatin accessibility (ATAC-seq) at 4654 BORIS binding sites that gained H2A.Z occupancy in NIH3T3 + BORIS (blue) cells compared to NIH3T3 + EV cells (black). *P*-value calculated by the Kolmogorov–Smirnov test. **g** Genome browser view of two alternative intronic promoters in Snx31 and Hsf3 genes activated by BORIS (blue) binding to CTCF (red) sites in NIH3T3 + BORIS (clone#2) cells. Black arrows indicate increased chromatin accessibility (ATAC-seq, black) beyond CTCF and BORIS ChIP-seq peaks. Red arrows show alternative transcription initiated from a CTCF binding site with BORIS recruitment

The observed epigenetic reprogramming of CTCF binding sites by BORIS demonstrates a significant increase in overall CTCF occupancy upon BORIS recruitment (Fig. 4h,i). To delve into this process, we examined CTCF occupancy in NIH3T3 cells before and after BORIS expression. The cells stably expressing BORIS (clone#2) exhibited thousands more CTCF binding sites compared to the EV control (Additional file 1: Fig. S15a). Normalized ChIP-seq tag density analysis of CTCF occupancy at the combined set of CTCF sites (81,231) revealed that the majority of new CTCF sites detected in BORIS-expressing cells were also bound by CTCF in NIH3T3 + EV cells, albeit with weaker signal (Fig. 6a,b). Notably, the detection of CTCF binding sites by ChIP-seq markedly increased in NIH3T3 + BORIS (clone#2) cells, possibly due to a more relaxed chromatin state induced by BORIS expression. Tag density analysis showed that CTCF occupancy increased by at least threefold at 6521 binding sites in NIH3T3 + BORIS (clone#2) cells compared to NIH3T3 + EV (Fig. 6b). In contrast, only five CTCF sites exhibited a more than threefold decrease in CTCF occupancy upon BORIS expression in NIH3T3 cells. A heatmap visualizing the 6521 CTCF sites with significantly increased CTCF occupancy demonstrated their strong association with BORIS recruitment (Fig. 6c). Furthermore, we observed de novo CTCF occupancy upon BORIS expression in NIH3T3 cells, leading to the activation of previously silent genomic regions. For instance, the endogenous Boris promoter was activated through the de novo recruitment of both CTCF and BORIS proteins (Additional file 1: Fig. S10b). Similarly, de novo activation of Isx and Dnah8 transcripts was associated with the appearance of new CTCF/BORIS and CTCF ChIP-seq peaks at the promoter regions and throughout the activated gene body, respectively (Additional file 1: Fig. S15b,c). Therefore, our data suggest that BORIS may act as a pioneer transcription factor, capable of opening chromatin and increasing accessibility for CTCF binding.

To test this theory, we identified genomic regions that altered their chromatin accessibility in response to BORIS expression by performing an assay for transposable-accessible chromatin sequencing (ATAC-seq) in NIH3T3 + BORIS (clone#2) and NIH3T3 + EV cells (Fig. 6d). As expected, we observed a shift towards more open chromatin states associated with ectopic BORIS expression (Additional file 1: Fig. S16a-d). Firstly, we detected an overall increase in chromatin accessibility at BORIS binding sites, with thousands of genomic sites where chromatin accessibility increased by more than twofold (Additional file 1: Fig. S16b). Secondly, there were over 5000 genomic regions that became either newly accessible or at least twice as accessible for Tn5 transposase activity in NIH3T3 + BORIS (clone#2) cells, compared to NIH3T3 + EV (Fig. 6d). Analysis of the newly accessible sites in these cells revealed a general association with BORIS binding, an increase in active histone marks (Fig. 6e, upper panel), and the activation of gene transcription (Additional file 1: Fig. S16e,f) related to the cellular processes upregulated in carcinogenesis (Additional file 1: Fig. S16h). In contrast, the 1439 genomic regions that lost or decreased chromatin accessibility by more than twofold in NIH3T3 + BORIS (clone#2) cells were neither associated with BORIS binding (Fig. 6e, lower panel) nor with a specific direction of gene transcription (Additional file 1: Fig. S16g), suggesting that these changes are secondary in the cascade of events involving ectopic BORIS expression. As established in this study, BORIS binding recruits SRCAP and leads to a de novo gain of H2A.Z occupancy (Fig. 5). Consistently, chromatin accessibility increased significantly and dramatically (Kolmogorov–Smirnov test, *p* = 0) at the 4654 BORIS sites with a de novo gain of H2A.Z occupancy (Fig. 6f, Additional file 1: Fig. S16d). An examination of individual CTCF sites that were epigenetically converted into active promoters (Additional file 1: Fig. S16i) demonstrated that BORIS binding not only opened chromatin for CTCF binding footprint, but also in the surrounding regions beyond the CTCF/BORIS peak (Fig. 6g). This suggests that the more relaxed chromatin state around the CTCF/BORIS binding sites may attract other transcription factors (TFs) and chromatin remodeling proteins to bind neighboring sequences for the initiation of de novo transcription.

### BORIS binding opens chromatin around CTCF sites, paving the way for other chromatin-binding factors to bind and activate transcription

**Fig. 7:**
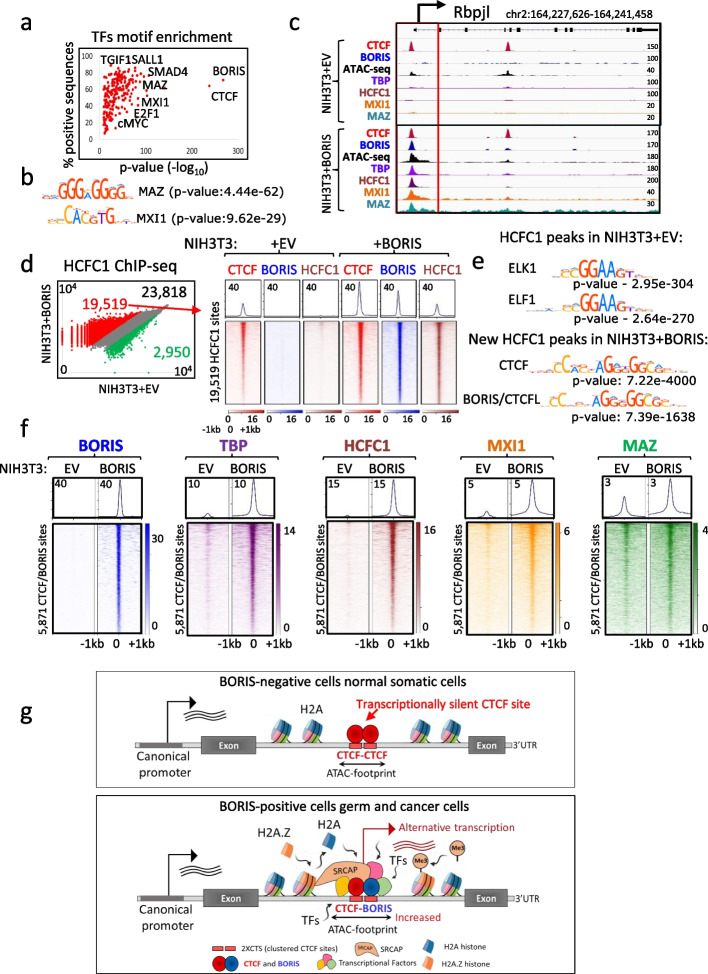
Opening of chromatin by BORIS facilitates binding of other transcriptional factors.** a** Scatter plot displaying the enrichment of 190 TF motifs at CTCF/BORIS binding sites that reprogrammed into active promoters, compared to transcriptionally silent CTCF/BORIS sites in NIH3T3 + BORIS (clone#2) cells. **b** MAZ and MXI1 motifs are significantly enriched at CTCF/BORIS binding sites converted into active promoters. **c** Genome browser view illustrates the recruitment of TBP, HCFC1, MXI1, and MAZ proteins at the CTCF site within the Rbpjl promoter, activated by BORIS binding in NIH3T3 + BORIS (clone#2) cells. The activated promoter is highlighted by a red open box. **d** Left panel: Scatter plot of normalized read counts (log_10_) for HCFC1 occupancy at the combined set of HCFC1 binding sites (46,287) in NIH3T3 + BORIS (clone#2) cells compared to the same genomic sites in EV cells. Right panel: Heatmap of CTCF (red), BORIS (blue), and HCFC1 (brown) occupancy at the 19,519 HCFC1 sites from the left panel (connected by red arrow). **e** TF motifs enriched at HCFC1 peaks (60 bp around the summit of peak) in NIH3T3 + EV versus NIH3T3 + BORIS (clone#2) cells. **f** Heatmap of BORIS (blue), TBP (purple), HCFC1 (brown), MXI1 (orange), and MAZ (green) occupancy at the 5871 CTCF/BORIS binding sites converted into active promoters in NIH3T3 + BORIS (clone#2) cells compared to NIH3T3 + EV cells from Fig. 4h. **g** Summary of epigenetic reprogramming: BORIS binding recruits SRCAP, which replaces H2A histone with H2A.Z, leading to the opening of chromatin around CTCF sites. This, in turn, attracts other TFs to bind and stimulate transcription, resulting in the conversion of transcriptionally inert CTCF sites into active promoters

We aimed to investigate whether the BORIS-mediated opening of chromatin (Fig. 6) indeed facilitates the recruitment of other transcription factors (TFs). Examination of RNAPII occupancy at BORIS binding sites revealed that, although the majority of sites were associated with active transcription in NIH3T3 + BORIS (clone#2) cells, not all of them were transformed into transcription start sites (TSSs) (Additional file 1: Fig. S17a). This suggests that other TFs may be necessary to initiate and drive transcription through cooperation, as is typically the case for promoter activation [70]. To delve into the differential opening of CTCF sites for transcription by BORIS, we conducted an analysis of TF motif enrichment. We compared the 5871 CTCF/BORIS binding sites that were epigenetically transformed into active promoters (Fig. 4h,i) with the CTCF/BORIS sites that lacked RNAPII enrichment and were not associated with active transcription. Our analysis revealed over 190 TF binding motifs that were significantly enriched (*p*-value < 3.39E − 20) around CTCF/BORIS binding sites converted into active promoters, compared to the transcriptionally silent CTCF sites (Fig. 7a, Additional file 6: Table S5). Furthermore, to identify potential corresponding BORIS protein partners, we overlapped the 1025 BORIS-bound K562-testis transcription start sites (TSSs) with all ChIP-seq data generated by ENCODE for the same cell type. In addition to the previously identified active histone modifications (H2A.Z, H3K4me3, H3K27ac) and RNAPII, we found that the TFs HCFC1 (Host Cell Factor C1), MAZ (Myc-associated zinc finger protein), TBP (TATA-binding protein), MXI1 (Max-interacting protein 1), and the histone demethylase PHF8 were also highly enriched at the 1025 TSSs (Additional file 1: Fig. S17b,c). Among these factors, both MAZ and MXI1 were among the 190 TFs significantly enriched at the 5871 CTCF/BORIS sites (Fig. 7b), as exemplified with MAZ protein binding to both the intronic GAL3ST1 and FERT promoters in K562 cells, along with BORIS (Additional file 1: Fig. S17d).

To investigate whether BORIS binding enables the recruitment of other TFs to chromatin, we performed ChIP-seq with TBP, HCFC1, MAZ, and MXI1 Abs in NIH3T3 + BORIS (clone#2) and control cells. ChIP-seq analysis showed that the number of mapped sites for all four TFs increased dramatically upon ectopic BORIS expression (Additional file 1: Fig. S18). Moreover, thousands of de novo binding sites for TFs were specifically associated with BORIS binding, indicating its direct involvement in their recruitment. For example, 14,533, 19,519, and 6237 of new TBP, HCFC1, and MXI binding sites, respectively, were mapped in NIH3T3 + BORIS (clone#2) cells following BORIS binding to CTCF sites (Fig. 7c,d, Additional file 1: Fig. S18a-c). Incidentally, the same sites were bound only by CTCF in control cells and did not have an enrichment in TF occupancy, suggesting that CTCF binding itself is not sufficient for these TFs to bind the chromatin around the sites (Fig. 7c, Additional file 1: Fig. S18a-c). HCFC1 occupancy increased dramatically with BORIS expression, as we mapped twice as many new HCFC1 binding sites (40,636) in NIH3T3 + BORIS (clone#2) cells, compared to EV cells (21,294 ChIP-seq peaks) (Additional file 1: Fig. S18b). All new HCFC1 ChIP-seq peaks were strongly and specifically associated with BORIS binding to CTCF sites (Fig. 7d). HCFC1, in contrast to other TFs (e.g. TBP, MXI1, and MAZ), is not associated with any specific DNA motif. As HCFC1 is known to have a weak DNA-binding domain, it depends on other factors for its recruitment to chromatin [71]. Previously, HCFC1 was identified as a partner of BORIS in the yeast two-hybrid system with the two probes representing the N-terminus of BORIS, similar to SRCAP [69]. To further confirm the role of BORIS in HCFC1 recruitment to chromatin, we analyzed new HCFC1 ChIP-seq peaks for the TFs motif enrichment in NIH3T3 cells prior to and after BORIS expression. This analysis highlighted CTCF and CTCFL/BORIS as the most significant motifs accompanying the new HCFC1 occupancy (Fig. 7e, lower panel). In control cells, the most enriched motifs under HCFC1 ChIP-seq peaks were ELK1 and ELF1, confirming the dependance of HCFC1 recruitment on other factors in BORIS-negative cells (Fig. 7e, upper panel). Moreover, analysis of HCFC1 binding sites in K562 cells with endogenous BORIS expression showed a strong correlation between HCFC1 and BORIS binding in this cancer cell line (Additional file 1: Fig. S19a,b). To investigate whether TBP, HCFC1, MXI1, and MAZ contribute to the initiation of transcription facilitated by BORIS, we examined their enrichment at the 5871 CTCF/BORIS sites that were converted into active promoters. Our analysis (Fig. 7f) confirmed that BORIS binding in NIH3T3 + BORIS (clone #2) cells acts as a prerequisite for other TFs to bind chromatin. Additionally, we validated the increased occupancy of TFs subsequent to BORIS binding in two independent clones derived from soft agar (#3 and #4) (Additional file 1: Fig. S19c,d). Moreover, the dox-induction time course illustrated the dependency of TF binding on BORIS recruitment to CTCF sites (Additional file 1: Fig. S19e). This suggests that BORIS may function similarly to pioneer transcription factors, facilitating the opening of chromatin around transcriptionally inert CTCF binding sites, thereby enabling other TFs to bind and consequently activating transcription.

## Discussion

Transcription factors and epigenetic modifiers govern cell identities by implementing cell type-specific transcriptional programs. The two paralogous genes, CTCF and BORIS, are normally co-expressed only during spermatogenesis. Their co-binding at the promoter regions of certain testis-specific genes is essential for male germ cell development [43]. The same testis-specific genes are aberrantly expressed in different types of cancer cells, coinciding with BORIS activation [14, 22, 23]. In this study, we demonstrate, for the first time, that BORIS can initiate the expression of testis-specific transcripts from transcriptionally inert, clustered CTCF binding sites. This initiation of transcription relies on the epigenetic reprogramming of the CTCF binding sites through BORIS-facilitated recruitment of chromatin remodeling factors and other TFs to a given CTCF site.

Activation of testis-specific transcription in somatic cells is a common hallmark of cancer [10, 72, 73]. However, whether the transcription of testis-specific genes is a cause or a consequence of carcinogenesis is yet to be determined. Several independent studies uncovered the role of CTCF and BORIS in the transcriptional regulation of testis-specific isoforms from GAL3ST1 and FER genes [9, 46, 47]. In this study, we showed that both GAL3ST1 and FER testis-specific transcripts are abnormally activated in cancer cells, coinciding with aberrant BORIS expression and its binding to the intronic promoters. We demonstrated that either transient or stable ectopic BORIS expression in BORIS-negative cells is sufficient to activate the promoters of the two CT genes from the intronic CTCF-bound sites. Based on these data, we propose that clustered CTCF binding sites possess an intrinsic quality that allows them to become active transcriptional start sites upon BORIS binding. Thus, in this study, we investigated how many clustered CTCF sites are, in fact, capable of turning into active promoters upon BORIS binding and what biochemical mechanisms are involved in this process. We first compared CTCF and BORIS genome-wide binding profiles in cancer cells (K562) with the transcriptional programs executed during both spermatogenesis and carcinogenesis. We found that multiple genes, similar to GAL3ST1 and FER genes, that are normally expressed from reference promoters in somatic cells, were in fact controlled by alternative intronic and intergenic promoters during male germ cell development and aberrantly activated in K562 cells. Using different cell systems with modified levels of BORIS protein, we demonstrated that BORIS co-binding with CTCF is essential for the activation of such alternative testis-specific promoters in cancers. The extent to which testis-specific alternative transcriptional start site usage takes place in cancer cells has not been previously systematically addressed; however, numerous efforts have been made to compile a list of CT genes [52, 74, 75]. Such efforts have proven useful in providing targets for cancer diagnostics and immunotherapy. In this study, we showed that the list of CT genes is, in fact, much more extensive than is currently accepted (if male germ cell-specific transcripts of genes expressed in somatic cells are included). Indeed, the examples we recovered in this study, including GAL3ST1, FERT, NOS3, PKH-T, and others, belong to the list of CT genes, which are not conventionally branded as CT genes.

The classic approach to explore a functional role of TFs in gene regulation is to ectopically overexpress a factor in TF-negative cells to predict its impact on gene expression. Multiple studies have reported that ectopic BORIS expression in BORIS-negative somatic cells is sufficient to activate some testis-specific gene transcription from a silent state [23, 36, 45, 47]. The question of whether BORIS itself can epigenetically reprogram its binding sites was approached in several studies as well. For example, Debruyne et al. reported an activation of BORIS expression in anaplastic lymphoma kinase (ALK)-mutated, MYCN-amplified neuroblastoma cells that develop resistance to ALK inhibition [21]. The increase of BORIS expression in ALK-resistant cells resulted in the gain of new BORIS-bound sites, which emerged together with a gain of H3K27ac mark, thus reprogramming the transcriptional outcome of ALK-resistant cells compared to parental cells. In another report, BORIS activation of CTA promoters in lung cancer was associated with a gain of active histone marks at BORIS binding sites [45]. However, the question of how BORIS can initiate transcription from a silent state and what the mechanism of epigenetic remodeling around its binding site is, was never addressed. In this study, we established the NIH3T3 cell model where ectopic BORIS expression is well tolerated by the cells. We demonstrated that NIH3T3 cells following ectopic BORIS expression were able to undergo soft agar selection and acquired a tumorigenic phenotype, possibly via the deregulation of multiple coding and noncoding transcripts. The analysis of epigenetic and transcriptomic changes in NIH3T3 cells, expressing either BORIS or EV showed that BORIS can epigenetically reprogram transcriptionally inert CTCF binding sites into active promoters.

How does BORIS transform CTCF binding sites into active promoters? BORIS is a testis-specific paralog of the ubiquitously expressed CTCF gene, which is a multifunctional 3D genome organizer. Hundreds of thousands of CTCF binding sites are positioned through mammalian genomes [76]. CTCF binding sites could be divided into the two major groups, based on the number of CTCF motifs present under ChIP-seq peaks [22]. The majority of single CTCF binding sites (1xCTSes) are located outside of gene promoter regions and are not associated with active transcription. In contrast, clustered CTCF binding sites (2xCTSes) are co-bound by CTCF and BORIS and are associated with active transcription in germ and cancer cells [22]. When BORIS is co-expressed with CTCF in the same cells, it forms heterodimers at the 2xCTSes, replacing CTCF homodimers at the same DNA regions [22, 43]. As the N- and C-termini of CTCF and BORIS lack homology, epigenetic and transcriptional outcomes resulting from such heterodimerization is distinct from CTCF homodimerization [22, 43]. Indeed, among previously mapped BORIS-interacting partners are multiple epigenetic remodeling proteins, which are not CTCF partners [77]. For example, epigenetic modifiers SRCAP, BAT3, SET1A, and PRMT7 have been shown to interact with BORIS, but not CTCF [69, 77, 78]. Epigenetic remodeling, which accompanies BORIS binding, has been shown to be associated with a change of transcription [78, 79]. Here, we have characterized the transcriptional outcome of CTCF-BORIS heterodimerization in somatic normal and cancer cells. Comparing different marks of active transcription, including histone modifications and RNAPII recruitment, we showed that BORIS binding at the 2xCTSes promotes profound epigenetic changes, such as a gain of H2A.Z histones at most BORIS binding sites. Furthermore, BORIS can directly interact with and recruit the chromatin remodeling protein, SRCAP, which replaces the H2A histone with H2A.Z. This creates a more open chromatin state, as we demonstrated in this study by the increased accessibility for Tn5 transposase activity. A known consequence of incorporating H2A.Z into the nucleosome is the destabilization of intra- and inter-nucleosome histone-histone interactions [80]. H2A.Z has also been proven to be sufficient to initiate transcription in yeast [81] and is involved in transcriptional machinery recruitment in eukaryotic cells [82, 83]. Additionally, unlike many other epigenetic marks, H2A.Z does not spread from the affected nucleosome to neighboring regions, making it an informative mark for chromatin remodeling dynamics [84]. Here we showed that the opening of chromatin through H2A.Z enrichment around BORIS sites stimulates the recruitment of other transcription factors, thus initiating transcription from intronic or intergenic alternative promoters. The enrichment of motifs for multiple transcription factors around CTCF/BORIS sites serves as a prerequisite for the conversion of clustered CTCF sites into active alternative promoters through BORIS binding. Besides a gain of H2A.Z histones around BORIS binding sites, there is also a gain of other histone modifications (H3Kme3, H3K27ac) known to correlate with active transcription. Since other tested histone marks are enriched to a much lesser extent than H2A.Z, as well as the dox-induction time course data show that the gain of H2A.Z precedes the gain of H3K4me3, we suggest that the appearance of other active histone modifications is likely a secondary event in the epigenetic reprogramming of CTCF/BORIS sites.

Transcription factors that can determine cell fate through activating genes silenced by repressive chromatin structures are called pioneer transcription factors. Such factors can bind transcriptionally inactive nucleosome-bound sites and remodel them, attracting additional transcription factors to bind and cooperate in the regulation of gene expression [85]. In this study, we demonstrated, for the first time, that BORIS functions akin to pioneer transcription factors by binding to chromatin. BORIS exhibits two distinct mechanisms for epigenetic remodeling, depending on the initial state of corresponding sites. The primary and most prevalent method involves its incorporation into transcriptionally silent CTCF-bound sites. Subsequently, these sites undergo epigenetic remodeling into active promoters through the recruitment of SRCAP, H2A.Z, and various transcription factors. The second mechanism involves BORIS binding to CTCF motifs not already bound by CTCF, which are within closed chromatin regions. Here, BORIS either recruits CTCF to these sites or binds independently (referred to as BORIS-only sites), resulting in chromatin remodeling in the vicinity. In both scenarios, the interaction between CTCF and BORIS emerges as pivotal for the epigenetic conversion of CTCF sites into functional promoters. It is noteworthy that in certain instances, BORIS-only sites also demonstrate an association with active transcription. CTCF itself is not sufficient for such conversion but serves as a precise landmark for BORIS to bind and reprogram CTCF sites in germ and cancer cells. Ectopic BORIS expression in NIH3T3 cells showed that BORIS transforms thousands of CTCF sites into active promoters, and some of these promoters drive spurious transcription, which was not annotated before. Such promiscuous transcription is a well-known signature of germ cell-specific transcription and could be evolutionarily beneficial for the emergence of new genes [5]. Thus, we would suggest that BORIS may be one of the factors that drives the development of new genes from CTCF binding sites during spermatogenesis.

Ectopic expression of BORIS in somatic cells, apart from activation of alternative promoters of coding genes, also results in the activation of lncRNAs and transposable elements (TEs). While protein-coding transcripts represent only 1% of transcribed RNAs, the majority (76–90%) of the human genome is transcribed into lncRNAs and TEs [86]. Moreover, lncRNAs are expressed at much higher levels within the male reproductive system compared to somatic tissues [87]. Some of these lncRNAs are consistently activated in cancers and have been shown to be involved in carcinogenesis [88]. Similarly, activation of TEs is characteristic for both spermatogenesis and carcinogenesis [89]. As we have detected hundreds of lncRNA transcripts driven from BORIS-bound promoters in both K562 and testes, we analyzed whether ectopic BORIS expression can deregulate the expression of lncRNAs and TEs in normal and cancer somatic cells. RNA-seq analysis of NIH3T3, MDA-MB-435, and MM057 cells ectopically expressing BORIS showed thousands of lncRNAs and TEs transcripts being up- or downregulated. There are multiple reports showing that lncRNAs and TEs regulate transcription of protein-coding genes [90]. However, whether the expression of coding genes could result in a deregulation of TEs hosted within these genes had not previously been investigated. Using our cell models with ectopic BORIS expression, we showed that BORIS binding to promoters of coding and noncoding genes activated their expression, which in turn induced transcription of TEs nested in said genes. The deregulation of TEs by ectopic BORIS expression is therefore a secondary event, as the majority of such TEs are not directly bound by BORIS. Interestingly, among the most upregulated TEs by BORIS was LINE1, the only known class of autonomously active retrotransposons in humans and mice [91]. LINE1 expression is accompanied by genomic instability and has become one indicator for the occurrence, development, and poor prognosis of many diseases, including many cancers [92, 93]. The deregulation of TEs may explain why the main upregulated pathways in response to ectopic BORIS expression in different cell lines are related to inflammation and antiviral responses. Inflammation pathways are known to be upregulated in cancers and in aging tissues [94], as well as BORIS overexpression was linked to the activation of inflammatory pathways [18, 22]. As a result of the present study, we would suggest that the activation of inflammatory pathways by BORIS is, at least in part, mediated by a massive deregulation of TEs harbored by long activated transcripts, thus triggering the accumulation of cytosolic double-stranded TE DNAs as it has been described in a previous study [60].

## Conclusions

Our results indicate that BORIS binding is sufficient to epigenetically convert transcriptionally inert CTCF binding sites into active promoters (Fig. 7g). Particularly, BORIS recruits SRCAP protein to the clustered CTCF sites. SRCAP replaces H2A histone with H2A.Z, thus, creating a more open and relaxed chromatin state. The resulting open chromatin attracts multiple transcription factors to bind and stimulate transcription (Fig. 7g). The BORIS-reprogrammed promoters then drive the transcription of both coding and noncoding RNAs, also triggering the expression of TEs. The derepression of TEs triggers the activation of inflammation pathways, which may in turn contribute to carcinogenesis. Thus, BORIS is able to initiate profound transcriptional and epigenetic changes in both germ and cancer cells. This further substantiates the role of BORIS in cancer development and provides evidence of a mechanism enabling BORIS, through CTCF binding sites, to contribute to tumorigenesis through the promiscuous and pervasive activation of alternative promoters.

## Methods

### Cell culture

K562 (obtained from ATCC), mutant K562 (clone#3,4,7 derived from WT K562), HEK293T (obtained from ATCC), MDA-MB-435 (obtained from NCI collection of NCI-60 cancer cell lines), MCF7 (obtained from NCI collection of NCI-60 cancer cell lines), and NIH3T3 (a gift from Dr. Joseph Califano, Johns Hopkins Medical Institutions, Baltimore, MD, USA) cell lines were grown in Dulbecco’s modified Eagle’s medium (DMEM) supplemented with 10% of fetal calf serum and penicillin–streptomycin. For the foci formation, NIH3T3 cells were grown in serum-free DMEM for 10 days. CH12 LX B lymphoma cell line was maintained in RPMI 1640 supplemented with 10% FBS, 1% penicillin/streptomycin, and 55 μM 2-β mercaptoethanol. CH12 wild-type and mutant cells were described previously [34, 95] and obtained as a gift from Dr. Rafael Casellas (NIH, NIAMS). Mutant K562 (clone#3,4,7) cells were made using Zinc Finger Nuclease and were characterized in our previous study [22]. The wild-type (WT) K562 single-cell clones (control cells) were generated through the transfection of a single ZFN mRNA. This is in contrast to the use of two ZFN mRNAs, which is employed to induce the dimerization of the FokI cleavage domain for DNA cleavage. All transfections, except dox-inducible plasmids, were done with the vector (pCpGvitro-hygro, InvivoGen) encoding either LacZ (EV, empty vector) or the open reading frame (ORF) of human BORIS. HEK293T cells were transiently transfected using jetPEI (Polyplus) without antibiotic pressure. MDA-MB-435 and MCF7 cells were transfected using the Cell Line Nucleofector® Kit V (Lonza Group Ltd) and propagated for 3 weeks under hygromycin pressure (150 mg/l); several single-cell clones stably growing under antibiotic selection were selected and analyzed by Western blot for the presence of ectopic BORIS expression. NIH3T3 cells were transfected using jetPEI (Polyplus) and, 48 h days later, plated in soft agar to grow without antibiotic pressure. Only NIH3T3 cells, which stably express BORIS, were able to produce multiple colonies in soft agar. Single-cell-derived NIH3T3 clones were extracted from soft agar and propagated under normal cell culture conditions under hygromycin selection (150 mg/l). Dox-inducible plasmids expressing either BORIS or luciferase (EV) were constructed on a template containing the tetracycline-responsive, autoregulated, bidirectional expression vector pBIG2i, described here [56]. NIH3T3 cells were transfected using jetPEI according to the manufacturer’s protocol and propagated for 3 weeks under hygromycin pressure (150 mg/l) to obtain cells with stably integrated plasmids. The transfected cells were grown in DMEM media supplemented with 10% Tet system approved FBS (ThermoFisher) to avoid any induction of BORIS expression due to a leaky promoter in the plasmid. The dox concentration (1 μg/mL) was optimized based on the maximum activation of Oct4B and Gal3st1 isoforms expression by dox-induced BORIS. To induce BORIS expression, 1 μg/mL of dox was added into the media for indicated number of hours or days and refreshed every 2 days in case of 5 days treatment. To downregulate H2A.Z expression, NIH3T3 cells with dox-inducible BORIS were transfected with ON-TARGETplus siRNA either against mouse H2A.Z (Horizon Discovery, Smart pool, Catalog #L-042994–01-0020) or non-targeting control pool (HorizonDiscovery, Catalog #D-001810–10-05) using polyplus-transfection INTERFERin® siRNA transfection reagent (Genesee Scientific, Catalog#55–129). All cells were routinely screened for mycoplasma contamination.

### ChIP-seq

For ChIP-seq, 2 × 10^6^ asynchronously growing cells were crosslinked with 1% formaldehyde for 10 min at room temperature followed by quenching with 125 mM glycine for 10 min, washed twice with 1 × phosphate buffered saline (PBS), and resuspended in chromatin immunoprecipitation (ChIP) lysis buffer (150 mM NaCl, 1% Triton X-100, 0.1% SDS, 20 mM Tris–HCl pH8.0, 2 mM EDTA). Chromatin was sheared to 200–600 bp and immunoprecipitated with 5 μg of antibody. Immunoprecipitated DNA was purified with QIAquick columns (ZymoResearch). DNA concentration was assessed with a Qubit4 (ThermoFisher), and 5–10 ng was used to generate sequencing libraries using either a TruSeq ChIP Sample Preparation Kit (Illumina, Inc., USA) or NEBNext UltraII DNA Library Prep Kit for Illumina (BioLabs, E7645S). Libraries were single-end or paired-end-sequenced on either an Illumina HiSeq 2000 or Illumina NextSeq550 platform. Most ChIP-seq experiments were performed with a minimum of two biological and/or technical replicates. Each replicate was analyzed individually and compared with other technical replicates. A list of all NGS data generated in this study are supplied in Additional file 7: Table S6.

### ChIP-Re-ChIP

Chromatin was prepared as for ChIP-seq as described previously [22]. Briefly, chromatin was immunoprecipitated using our custom-made BORIS monoclonal antibodies [22], which were chemically crosslinked to magnetic beads using crosslinking buffer (0.2 M triethanolamine pH 8.2, 20 mM DMP), 30 min at room temperature. After overnight incubation with crosslinked antibodies, the chromatin was washed and eluted three times using elution buffer (0.1 M glycine–HCl pH 2.5). Eluted chromatin was neutralized using 1 M Tris (pH 8) and used for the second round of ChIP with custom-made CTCF monoclonal antibodies following the standard ChIP protocol as described above. As a control, we used NIH3T3 + EV cells with the same two rounds of immunoprecipitation. ChIP-Re-ChIP peaks were called using cut off more than 100 of − 10*log_10_ (*q*-value) significance.

### Antibodies used in ChIP-seq

Custom-made mouse monoclonal CTCF antibodies were previously described. [96]. Custom-made mouse monoclonal BORIS antibodies were also described previously [22]. Rabbit polyclonal antibody against SRCAP (Kerafast, ESL103), rabbit polyclonal against HCFC1 (Novus Biologicals, NB100-68209), goat affinity-purified polyclonal antibody against Mxi1 (R&D Systems, AF4185), rabbit monoclonal to TATA-binding protein TBP (Abcam, ab220788), rabbit polyclonal anti-PHF8 antibody (Bethyl Laboratories, A301-772A), rabbit polyclonal to MAZ (Abcam, ab85725), rabbit polyclonal antibody against the region of histone H3 containing the trimethylated lysine 27 (H3K27me3) (Diagenode, C15410195), rabbit polyclonal antibody against histone H3, trimethylated at lysine 36 (H3K36me3) (Diagenode, C15410058), rabbit polyclonal antibody against histone H2A.Z (Abcam, ab4174), rabbit polyclonal to Histone H3 (acetyl K27) (Abcam, ab4729), rabbit polyclonal to Histone H3 (tri methyl K4) (Abcam, ab8580), anti-RNA Polymerase II Antibody, CTD Antibody, clone 8WG16 (Millipore, 05–952-I-100UG).

### ATAC-seq

NIH3T3 cells (5 × 10^4^) were pelleted by centrifugation and resuspended in 50 µL of cold lysis buffer (10 mM Tris–HCl, pH 7.4, 10 mM NaCl, 3 mM MgCl2, 0.1% IGEPAL CA-630), gently pipetting up and down ten times. Nuclei were pelleted by centrifugation at 2500* g* for 10 min at 4 °C and resuspended in 25 µL of 2 × TD Buffer (Illumina Cat #FC-121–1030), containing 8 µL of Tn5 Transposes (Illumina Cat #FC-121–1030) and 17 µL of nuclease-free water. The reaction was incubated for 30 min at 37 °C. Tagmented DNA was purified by using a Zymo Research DNA Clean & Concentrator™-5—Capped Columns. The libraries were amplified using NEBNext High-Fidelity 2 × PCR Master Mix (New England Biolabs, M0541). The libraries were size-selected by adding 1.8X volume Agencourt AMPure XP beads (Beckman, 63881). Library concentration was measured by DNA High Sensitivity Kit (Invitrogen) on a Qubit fluorometer (Invitrogen). Library quality and fragment sizes were assessed on a Bioanalyzer (Agilent). ATAC-Seq libraries from three biological replicates for each condition were paired-end-sequenced on an Illumina NextSeq550 platform.

### RNA-seq

The RNA sequencing library preparation and sequencing procedures were carried out according to Illumina protocols. Briefly, poly(A)-mRNA was purified from 5 μg of RNA with streptavidin-coated magnetic beads. After chemical fragmentation, mRNA fragments were reverse-transcribed and converted into double-stranded cDNA. Following end repair and A-tailing, paired-end adaptors were ligated to the ends of the DNA fragments. The ligated products were amplified with 18 cycles of PCR followed by purification using AMPure beads (Beckman Coulter). The enriched libraries were diluted to a final concentration of 5 nM. RNA-Seq libraries from at least two biological and technical replicates for each cell type and condition were single-end or paired-end sequenced on either an Illumina HiSeq 2000 or NovaSeqS1 6000 platform.

### CAGE-seq

CAGE-seq libraries (human testes and NIH3T3 cells) were performed by DNAFORM (Yokohama, Kanagawa, Japan) according to their protocol and using total RNA provided by us. Total RNA extracted from testis was purchased from OriGene Technologies. Three replicates of human testes RNAs were extracted from different donors, all younger than 45 years old. Total RNA was extracted from NIH3T3 cells by Invitrogen™ TRIzol reagent according to the manufacturer’s recommendations. Quality of total RNA was assessed by Agilent 2100 Bioanalyzer.

### Bioinformatic analysis of ChIP-seq data

Sequences generated by the Illumina genome analyzer (36–60 bp reads) were aligned against either the human (build hg19) or mouse (build mm9) genome using the Bowtie2 program with the default parameters [97]. The reproducibility of ChIP-seq data between technical replicates was assessed by computing the Pearson correlation coefficient (PCC) based on mapped read counts within MACS-called genomic regions. Initially, the MACS2 peak calling algorithm was applied independently for each replicate. Subsequently, the called ChIP-seq peaks from each replicate were merged into one combined set to calculate the PCC between replicates. All ChIP-seq data replicates exhibited an optimal range of correlation, typically higher than 0.85 to 0.95, ensuring robust reproducibility. To call the final ChIP-seq peaks, the two BAM file replicates were merged, duplicate reads were removed, and the MACS2 algorithm was applied. The ChIP-seq data were visualized using the Integrative Genomics Viewer (IGV) [98]. The peak overlaps between ChIP-seq data sets were determined with the BedTools Suite [99]. We defined peaks as overlapping if at least 1 bp of reciprocal peaks intersect. The normalized tag density profiles were generated using the BedTools coverage option from the BedTools Suite [99], normalized to the number of mapped reads, and plotted by Microsoft Excel. The heatmaps and the average profiles of ChIP-Seq tag densities for different clusters were generated using either seqMINER 1.3.3 platform [100] or using DeepTools [101]. We used *k*-means ranked method for clustering normalization. Position weight matrices were calculated using Multiple EM for Motif Elicitation (MEME) software [102]. The sequences under the summit of ChIP-seq peaks were extended 100 bp upstream and downstream for motif discovery. We ran MEME with parameters (-nmotifs 1 -mod oops -revcomp -w 14) to identify the motif under HCFC1, CTCF, and BORIS ChIP-seq peaks. The Kolmogorov–Smirnov test was used to calculate the *p*-value for ChIP-seq average plots in Additional file 1: Fig. S11a and Fig. S14k, using "ks.test" function of R (version 4.0.4). To assess the presence of CTCF motifs within sequences occupied by either CTCF, BORIS, or both proteins simultaneously, we employed FIMO software from the MEME suite using default parameters. Each occurrence of the CTCF motif displayed a *p*-value of < 0.0001 within the 200 bp sequences around the summit of either CTCF (CTCF-only and CTCF&BORIS-bound regions) or BORIS (BORIS-only bound regions).

### Bioinformatic analysis of RNA-seq data

FASTQ files were either mapped to the UCSC mouse reference genome (build mm9) or human ((build hg19) reference genomes using STAR [103] with default settings using either NCBI RefSeq Gene annotation [104] or long noncoding RNA database [105] or Repetitive Elements database [106] or Retroposed Genes [107] annotations as a guide for the assembly of expressed transcripts. To call novel transcripts activated by BORIS in NIH3T3 cells (soft-agar colonies), we used StringTie [108] to assemble all transcripts expressed in NIH3T3 + BORIS cells. RefSeqGene and NONCODE annotations were used as a guide for the assembly of novel transcripts. Next, we removed the known transcripts, using NONCODE and RefSeqGene annotation, resulting in a gtf file with the “novel/new” transcripts. Normalized gene counts and differentially expressed genes (DEG) were obtained with the R package DESeq2 (Love, Huber et al., 2014). The expression of each transcript was quantified as the number of reads mapping to a transcript divided by the transcript length in kilobases and the total number of mapped reads in millions (FPKM). Transcripts having more than log_2_ > 1.3 changes in their expression and FDR < 0.05 were further analyzed. Gene Set Enrichment Analysis (GSEA) was performed using the GSEA v4.1.0 software [109] and the following Molecular Signatures Databases (MSigDB): the Hallmark gene sets (H), Canonical Pathways gene sets derived from the KEGG pathway database (C2: KEGG), and the Gene sets derived from the GO Biological Process ontology (C5:BP) (http://www.gsea-msigdb.org/gsea/msigdb/collections.jsp). We chose the Signal2Noise metric for ranking genes, and 1000 gene set permutations were used to generate a null distribution for the enrichment score, which was used to yield a normalized enrichment score (NES) for the gene sets. Functional enrichment analysis of 790 reference genes corresponding to the 1025 TSSs was performed by Ingenuity Pathway Analysis (IPA). In the case of dox-inducible NIH3T3 cells, we conducted two sets of RNA-seq experiments using paired-end sequencing, one with 50-bp reads and the other with 100-bp reads from both ends. When calculating the list of differentially expressed genes (DEGs), using basemean > 10, log_2_ > 1.3, *p*-value < 0.001, we ensured that we compared RNA-seq libraries that were prepared and sequenced with the same read length for accurate comparisons. The cells with stable expression of either empty vector (EV) or BORIS were compared against each other in pairs, with respect to both treatment with doxycycline and without.

### Bioinformatic analysis of ATAC-seq data

Raw reads were qualified with FastQC tool (http://www.bioinformatics.babraham.ac.uk/projects/fastqc). Processed reads were aligned to the mm9 genome using Bowtie2 [110] with options “–very-sensitive -X 2000.” Sorting and indexing of bam files was performed with SAMTools [111]. Open chromatin regions were called using MACS2 [112] with options: –nomodel – shift -100 – extsize 200 -f BAMPE. Bdg files were generated by MACS2. Normalized bigWig files were generated from the filtered BAM files using DeepTools [101] bamCoverage with the options –normalizeUsing “RPKM.” The normalized bigWig files were used in subsequent analyses for heatmap generation. The evaluation of ATAC-seq data reproducibility through Pearson correlation coefficients among three technical replicates demonstrated an optimal value of greater than 0.9.

### Bioinformatic analysis of CAGE-seq data

The CAGE data were trimmed and quality controlled using FASTX Toolkit. The trimmed reads were mapped to either mouse (mm9) or human (hg19) genomes using Bowtie with the following parameters: -n2, -m20, -strata, -best. The genome-wide coverage data were generated in deepTools (3.5.0) with bamCoverage command (parameters: –normalizeUsing RPKM). The clustering of CAGE-seq reads were analyzed by MACS2 using options: callpeak -f BAM –nomodel –shift -1. The significant (*p*-value < 0.0005) clusters of CAGE-seq reads with more than 1 read/tag per million mapped reads were counted in as TSSs. CAGE-seq mapped reads were split into positive and negative strands with Samtools and then visualized in the IGV genome browser.

### Heatmaps, average, and scatter plots for NGS data visualization

To create NGS heatmaps, we applied deepTools using bamCoverage to generate normalized bigWig files, in combination with computeMatrix and plotHeatmap [101]. To create average plots, we used HOMER annotatePeaks.pl option. To generate scatter plots between two different conditions, EV versus BORIS, BAM files were converted into BED files using the bamToBed option of BEDTools [99]. Duplicated reads were removed using filterdup MACS2 with option –keep-dup = 1. Next, all BED files were normalized to 30 million reads using a custom script. The normalized BED files were used to calculate the number of reads mapped at each base pair position of genomic regions mapped by either ChIP-seq, ATAC-seq, or CAGE-seq using the bedtools coverage option of BEDTools. The set of genomic regions used for scatter plots to calculate read/tag density was created by combining all genomic regions mapped in both conditions. For example, in Fig. 6b, all CTCF binding sites mapped by ChIP-seq in either NIH3T3 + EV or NIH3T3 + BORIS (clone#2) cells were combined in one set of CTCF binding sites, and the genomic regions extended 500 bp upstream and downstream from the summit of CTCF ChIP-seq peak were used for scatter plots. Scatter plots for the number of reads mapped at each base pair position were generated by Excel. For a heatmap analysis of the 790 genes (Additional file 1: Fig. S2c), we used the Gene Set Enrichment Analysis (GSEA) tool [109], using gene expression by the Global Cancer Map data [50].

### RT-qPCR protocol

Total RNA was extracted with Trizol reagent (Invitrogen, CA, USA) according to the manufacturer’s instructions. RNA quantity and quality were measured using Nanodrop. 2.5 µg of RNA was used as a template for reverse-transcriptase PCR, following the manufacturer protocol (SuperScript™ IV VILO™ Master Mix (ThermoFisher). The cDNA was diluted 10 × , and 1 μl was used for qPCR amplification, following the manufacturer protocol (SYBR™ Green PCR Master Mix (ThermoFisher). Quantitative PCR was performed using the 7900HT sequence detection system (Applied Biosystems). GAPDH expression was used as an internal control for gene expression normalization. A statistical test between normalized gene expression in each sample was performed using two-tailed Student’s *t* test. Additional file 8: Table S7 contains primers sequences used for RT-qPCR.

### Western blotting

Protein extracts were prepared with RIPA Lysis buffer (Millipore) containing 50 mM Tris–HCl, pH 7.4, 1% Nonidet P-40, 0.25% sodium deoxycholate, 500 mM NaCl, 1 mM EDTA, and 1 × protease inhibitor cocktail (Roche Applied Science). To detect LGALS3BP secreted protein, cell supernatant with trichloroacetic acid precipitation was prepared according to protocol [113]. The protein extracts were resolved by SDS-PAGE, transferred to a PVDF membrane, and incubated with the indicated antibodies. Detections were performed using ECL reagents. The antibodies used are as follows: custom-designed mouse monoclonal antibody against BORIS, mouse monoclonal antibody against CTCF (Santa Cruz, sc-271514), mouse monoclonal antibody against Tubulin (Abcam, ab7291), rabbit polyclonal antibody against SRCAP (Kerafast, ESL103), Rabbit IgG (Abcam, ab37415), rabbit monoclonal against histone H3 (Abcam, ab201456), rabbit monoclonal against LINE-1 ORF1p (Abcam, ab216324), rabbit monoclonal against IRF7 (Thermo Fisher Scientific, SC0617), rabbit polyclonal against LGALS3BP (Thermo Fisher Scientific, PA5-79597), rabbit polyclonal against IFIT1 (Abcam, ab236256), rabbit polyclonal against IFI44 (Thermo Fisher Scientific, PA5-96967), rabbit polyclonal against Aim2 (Cell Signaling, #63660), rabbit polyclonal against OAS1 (MyBioSource, MBS129033).

### Co-immunoprecipitation assay

For co-immunoprecipitation reactions, we used Nuclear Complex Co-IP Kit (Active Motif54001) according to the manufacturer’s recommendations. Briefly, 200 µg of nuclear extract was used per IP reaction and incubated with 3 µg of the corresponding antibody. Some of the nuclear extracts were treated with 125 U/mL of benzonase (Millipore-Sigma, 71205-M) on ice for 90 min. The antibody/extract mixture was incubated overnight at 4°C on a rotator. Protein G beads were added to each IP reaction. We used IP buffers from the Kit, containing low salt and detergent. Following the IP, 2X sample buffer was added to each IP reaction; samples were boiled and run on an SDS-PAGE gel. The antibodies used for Co-IP and detection: custom-designed mouse monoclonal antibody against BORIS, mouse monoclonal antibody against CTCF (Santa Cruz, # sc-271514), rabbit polyclonal antibody against SRCAP (Kerafast, ESL103), Rabbit IgG (Abcam, ab37415), and p400 (Novus, NB200-210). Immunoprecipitated samples were resolved by SDS-PAGE, transferred to a PVDF membrane, and incubated with the indicated antibodies. Detections were performed using ECL reagents.

### Supplementary Information

**Additional file 1:** **Fig. S1.** The co-binding of CTCF and BORIS to the intronic regions of GAL3ST1 and FER genes is linked to the activation of alternative testis-specific promoters. **Fig. S2.** BORIS binding is associated with activation of cancer-testis-specific transcription. **Fig. S3.** The association between BORIS binding and testis-specific transcripts in K562 cells. **Fig. S4.** The absence of serum in the media reveals the transformed characteristics of NIH3T3 cells that express BORIS. **Fig. S5.** The impact of ectopic BORIS expression on transcriptional alterations in NIH3T3 cells. **Fig. S6.** Ectopic BORIS expression induces transcriptional changes in human melanoma cancer cells, MDA-MB-435 and MM057. **Fig. S7.** Doxycycline-induced BORIS expression leads to the deregulation of transcription in NIH3T3 cells. **Fig. S8.** Ectopic BORIS expression in NIH3T3 cells initiates the deregulation of transposable elements and the activation of inflammation pathways. **Fig. S9.** The activation of an alternative intronic promoter in the Oct4 gene mediated by BORIS. **Fig. S10.** BORIS binding initiates a cascade of epigenetic changes that transform clustered CTCF sites into active promoters. **Fig. S11.** BORIS binding is accompanied by the acquisition of active histone marks. **Fig. S12.** BORIS binding results in the acquisition of ative transcription markers. **Fig. S13.** The epigenetic reprogramming of 5,871 clustered CTCF sites by BORIS in NIH3T3 cells. **Fig. S14.** BORIS recruits SRCAP, which leads to heightened H2A.Z histone incorporation around BORIS binding sites, consequently promoting transcriptional activation. **Fig. S15.** BORIS binding facilitates the de novo occupancy of CTCF. **Fig. S16.** The binding of BORIS results in chromatin opening that extends beyond the CTCF site. **Fig. S17.** In K562 cells, TSSs bound by BORIS show co-binding with several other transcription factors. **Fig. S18.** BORIS binding paves the way for other chromatin-binding factors to bind. **Fig. S19. **BORIS binding is accompanied by the occupancy of HCFC1 and TBP

**Additional file 2:** **Supplementary Table S1.** A list of TSSs identified through CAGE-seq across various human cell tissues.

**Additional file 3:** **Supplementary Table S2.** The overlapping of BORIS binding sites identified in K562 cells with CAGE-seq data obtained from both K562 and testes samples.

**Additional file 4:** **Supplementary Table S3.** A list of newly identified transcripts in NIH3T3 cells.

**Additional file 5:** **Supplementary Table S4.** A list of pathways deregulated upon ectopic BORIS expression in NIH3T3 cells.

**Additional file 6:** **Supplementary Table S5.** A list of significantly enriched transcription factor (TF) binding motifs at CTCF/BORIS binding sites converted into active promoters.

**Additional file 7:** **Supplementary Table S6.** A list of all NGS data generated in this study.

**Additional file 8**: **Supplementary Table S7.** A list of primers sequences used for RT-qPCR.

**Additional file 9.** Uncropped images for the blots in figures 1, 3 and 5 and supplementary figure S8, S14.

**Additional file 10.** Review history.

## Data Availability

The next-generation sequencing datasets generated in this study for ChIP-seq, RNA-seq, ATAC-seq, and CAGE-seq are available in NCBI Gene Expression Omnibus (GEO) repository, under accession number GSE207058: https://www.ncbi.nlm.nih.gov/geo/query/acc.cgi?acc=GSE207058 [114]. All other data supporting the findings of this study are available from the corresponding author upon reasonable request. The published next-generation experiments used in this study are CTCF and BORIS ChIP-seq data in K562 cell line from GSE70764 [22]. ENCODE ChIP-seq data for K562 and NHEK cell lines used in the study are as follows: GSE29611 [115], GSE31477 [116], GSE35583 [117]. ENCODE RNA-seq data for K562 and NHEK cells used in this study is GSE33480 [118]. ENCODE ChIA-PET data for H3K27ac in K562 is GSM1436262 [119]. CAGE-seq for whole cell polyA + for K562 and NHEK cells is GSE34448 [120]. CAGE-seq data for normal human and mouse tissues were downloaded from FANTOM 5 (Functional ANnoTation of Mammalian Genome) collection [121]. RNA-seq data for MM057 melanoma cell line, with dox-inducible BORIS expression, were downloaded from this study [19]. No other scripts and software were used other than those mentioned in the “Methods” section.

## Abbreviations

BORIS: Brother of the regulator of imprinted sites
ChIP: Chromatin immunoprecipitation
CTCF: CCCTC-binding factor
CTS: CTCF target site
2xCTSes: CTCF target sites with two CTCF consensuses
1xCTSes: CTCF target sites with one CTCF consensus
SRCAP: Snf2 Related CREBBP Activator Protein
TEs: Transposable elements
CAGE: Cap analysis of gene expression
PCR: Polymerase chain reaction
RNAPII: RNA polymerase II
TSS: Transcription start site
UCSC: University of California, Santa Cruz
ZF: Zinc finger
ZFN: Zinc finger nuclease
