# Supplementary material for: BORIS/CTCFL epigenetically reprograms clustered CTCF binding sites into alternative transcriptional start sites
Source: Genome Biol. 2024 Jan 31;25:40. doi: 10.1186/s13059-024-03175-0 (PMC10832218; doi:10.1186/s13059-024-03175-0)
Supplement: Supplementary file 1 — Additional file 1: Fig. S1. The co-binding of CTCF and BORIS to the intronic regions of GAL3ST1 and FER genes is linked to the activation of alternative testis-specific promoters. Fig. S2. BORIS binding is associated with activation of cancer-testis-specific transcription. Fig. S3. The association between BORIS binding and testis-specific transcripts in K562 cells. Fig. S4. The absence of serum in the media reveals the transformed characteristics of NIH3T3 cells that express BORIS. Fig. S5. The impact of ectopic BORIS expression on transcriptional alterations in NIH3T3 cells. Fig. S6. Ectopic BORIS expression induces transcriptional changes in human melanoma cancer cells, MDA-MB-435 and MM057. Fig. S7. Doxycycline-induced BORIS expression leads to the deregulation of transcription in NIH3T3 cells. Fig. S8. Ectopic BORIS expression in NIH3T3 cells initiates the deregulation of transposable elements and the activation of inflammation pathways. Fig. S9. The activation of an alternative intronic promoter in the Oct4 gene mediated by BORIS. Fig. S10. BORIS binding initiates a cascade of epigenetic changes that transform clustered CTCF sites into active promoters. Fig. S11. BORIS binding is accompanied by the acquisition of active histone marks. Fig. S12. BORIS binding results in the acquisition of ative transcription markers. Fig. S13. The epigenetic reprogramming of 5,871 clustered CTCF sites by BORIS in NIH3T3 cells. Fig. S14. BORIS recruits SRCAP, which leads to heightened H2A.Z histone incorporation around BORIS binding sites, consequently promoting transcriptional activation. Fig. S15. BORIS binding facilitates the de novo occupancy of CTCF. Fig. S16. The binding of BORIS results in chromatin opening that extends beyond the CTCF site. Fig. S17. In K562 cells, TSSs bound by BORIS show co-binding with several other transcription factors. Fig. S18. BORIS binding paves the way for other chromatin-binding factors to bind. Fig. S19. BORIS binding is accompanied by the occ [file 13059_2024_3175_MOESM1_ESM.pptx]

## Slide 1
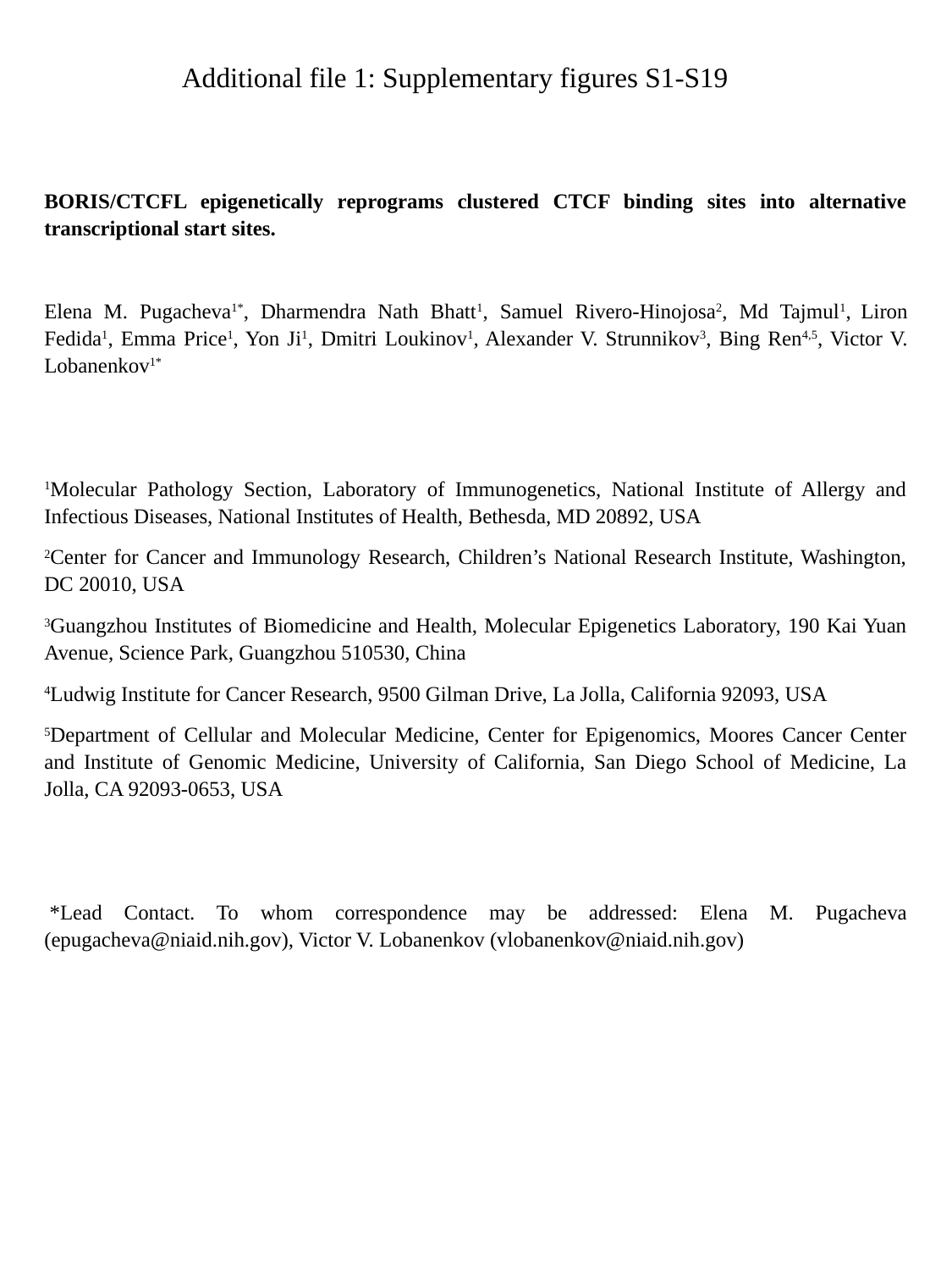

Additional file 1: Supplementary figures S1-S19
BORIS/CTCFL epigenetically reprograms clustered CTCF binding sites into alternative transcriptional start sites.
Elena M. Pugacheva1*, Dharmendra Nath Bhatt1, Samuel Rivero-Hinojosa2, Md Tajmul1, Liron Fedida1, Emma Price1, Yon Ji1, Dmitri Loukinov1, Alexander V. Strunnikov3, Bing Ren4,5, Victor V. Lobanenkov1*
1Molecular Pathology Section, Laboratory of Immunogenetics, National Institute of Allergy and Infectious Diseases, National Institutes of Health, Bethesda, MD 20892, USA
2Center for Cancer and Immunology Research, Children’s National Research Institute, Washington, DC 20010, USA
3Guangzhou Institutes of Biomedicine and Health, Molecular Epigenetics Laboratory, 190 Kai Yuan Avenue, Science Park, Guangzhou 510530, China
4Ludwig Institute for Cancer Research, 9500 Gilman Drive, La Jolla, California 92093, USA
5Department of Cellular and Molecular Medicine, Center for Epigenomics, Moores Cancer Center and Institute of Genomic Medicine, University of California, San Diego School of Medicine, La Jolla, CA 92093-0653, USA
 *Lead Contact. To whom correspondence may be addressed: Elena M. Pugacheva (epugacheva@niaid.nih.gov), Victor V. Lobanenkov (vlobanenkov@niaid.nih.gov)

## Slide 2
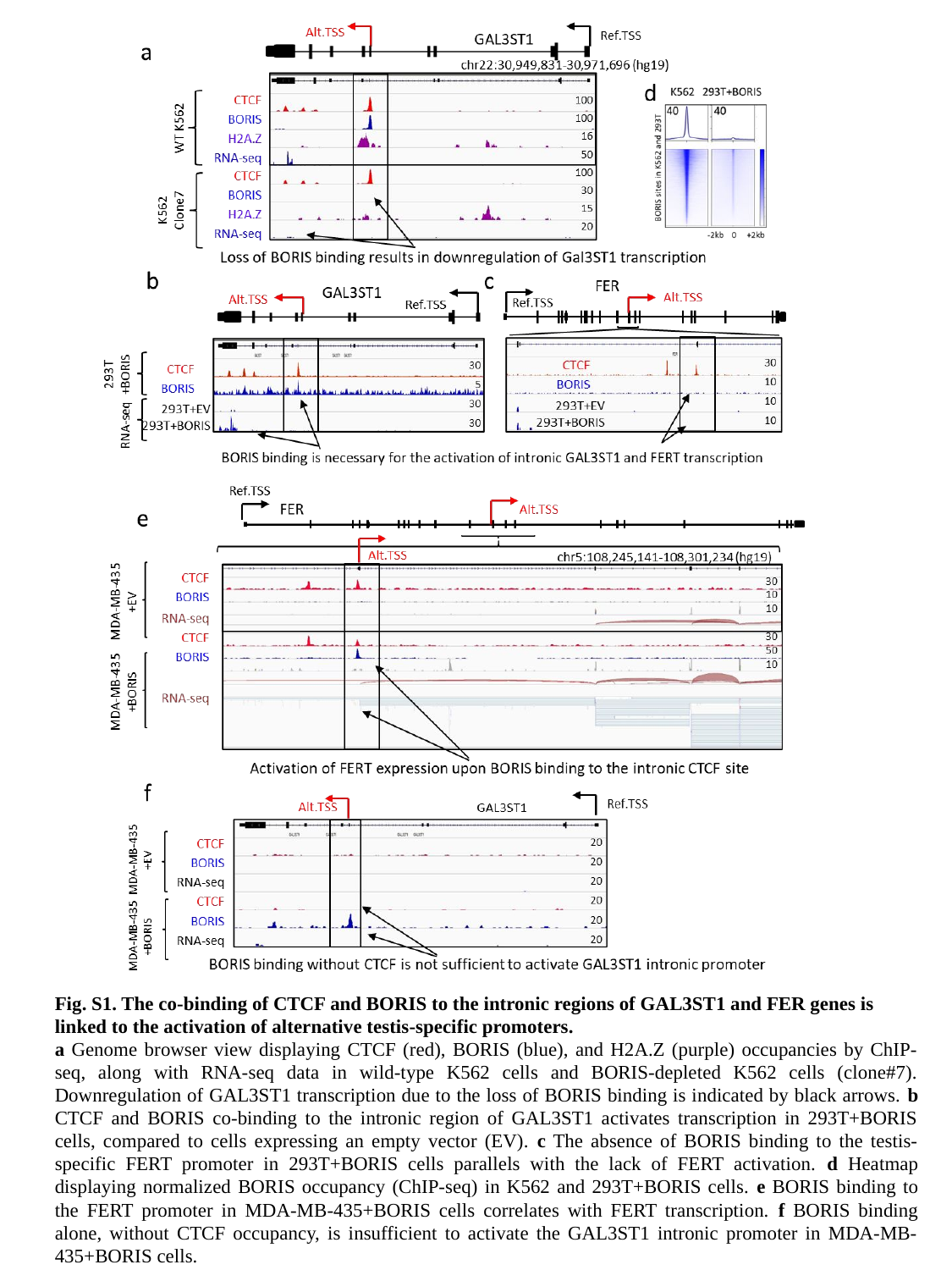

Fig. S1. The co-binding of CTCF and BORIS to the intronic regions of GAL3ST1 and FER genes is linked to the activation of alternative testis-specific promoters.
a Genome browser view displaying CTCF (red), BORIS (blue), and H2A.Z (purple) occupancies by ChIP-seq, along with RNA-seq data in wild-type K562 cells and BORIS-depleted K562 cells (clone#7). Downregulation of GAL3ST1 transcription due to the loss of BORIS binding is indicated by black arrows. b CTCF and BORIS co-binding to the intronic region of GAL3ST1 activates transcription in 293T+BORIS cells, compared to cells expressing an empty vector (EV). c The absence of BORIS binding to the testis-specific FERT promoter in 293T+BORIS cells parallels with the lack of FERT activation. d Heatmap displaying normalized BORIS occupancy (ChIP-seq) in K562 and 293T+BORIS cells. e BORIS binding to the FERT promoter in MDA-MB-435+BORIS cells correlates with FERT transcription. f BORIS binding alone, without CTCF occupancy, is insufficient to activate the GAL3ST1 intronic promoter in MDA-MB-435+BORIS cells.

## Slide 3
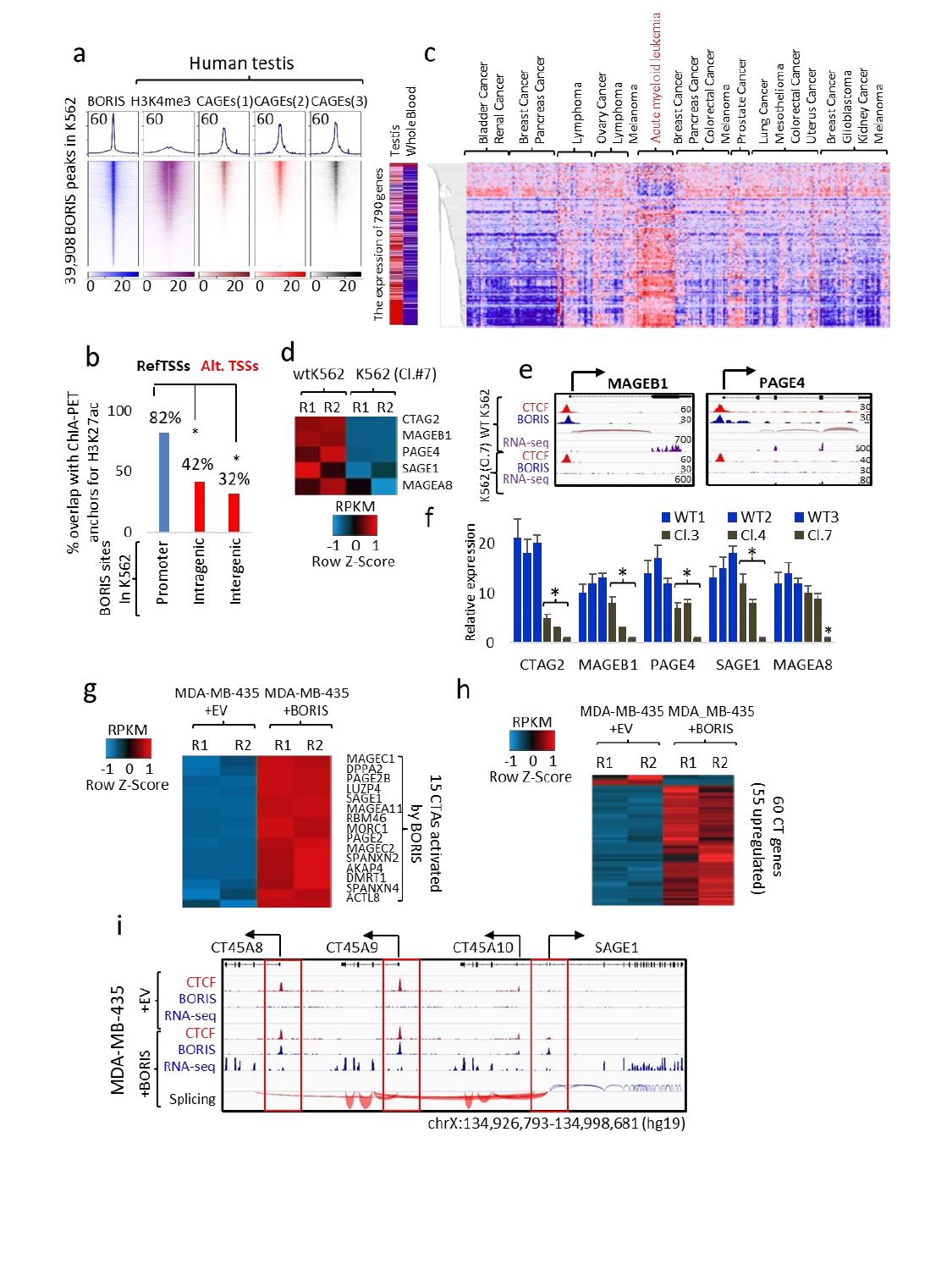

## Slide 4
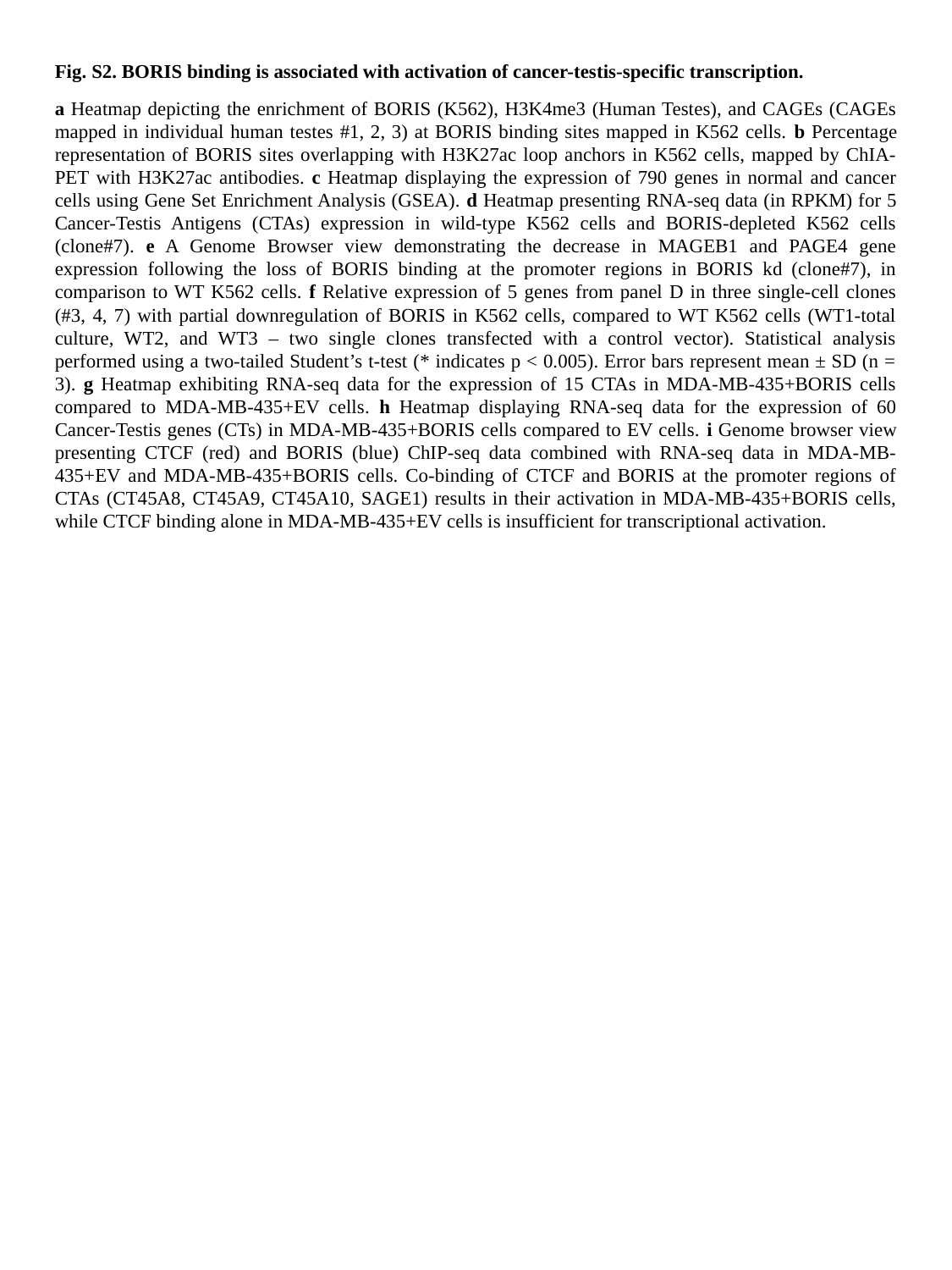

Fig. S2. BORIS binding is associated with activation of cancer-testis-specific transcription.
a Heatmap depicting the enrichment of BORIS (K562), H3K4me3 (Human Testes), and CAGEs (CAGEs mapped in individual human testes #1, 2, 3) at BORIS binding sites mapped in K562 cells. b Percentage representation of BORIS sites overlapping with H3K27ac loop anchors in K562 cells, mapped by ChIA-PET with H3K27ac antibodies. c Heatmap displaying the expression of 790 genes in normal and cancer cells using Gene Set Enrichment Analysis (GSEA). d Heatmap presenting RNA-seq data (in RPKM) for 5 Cancer-Testis Antigens (CTAs) expression in wild-type K562 cells and BORIS-depleted K562 cells (clone#7). e A Genome Browser view demonstrating the decrease in MAGEB1 and PAGE4 gene expression following the loss of BORIS binding at the promoter regions in BORIS kd (clone#7), in comparison to WT K562 cells. f Relative expression of 5 genes from panel D in three single-cell clones (#3, 4, 7) with partial downregulation of BORIS in K562 cells, compared to WT K562 cells (WT1-total culture, WT2, and WT3 – two single clones transfected with a control vector). Statistical analysis performed using a two-tailed Student’s t-test (* indicates p < 0.005). Error bars represent mean ± SD (n = 3). g Heatmap exhibiting RNA-seq data for the expression of 15 CTAs in MDA-MB-435+BORIS cells compared to MDA-MB-435+EV cells. h Heatmap displaying RNA-seq data for the expression of 60 Cancer-Testis genes (CTs) in MDA-MB-435+BORIS cells compared to EV cells. i Genome browser view presenting CTCF (red) and BORIS (blue) ChIP-seq data combined with RNA-seq data in MDA-MB-435+EV and MDA-MB-435+BORIS cells. Co-binding of CTCF and BORIS at the promoter regions of CTAs (CT45A8, CT45A9, CT45A10, SAGE1) results in their activation in MDA-MB-435+BORIS cells, while CTCF binding alone in MDA-MB-435+EV cells is insufficient for transcriptional activation.

## Slide 5
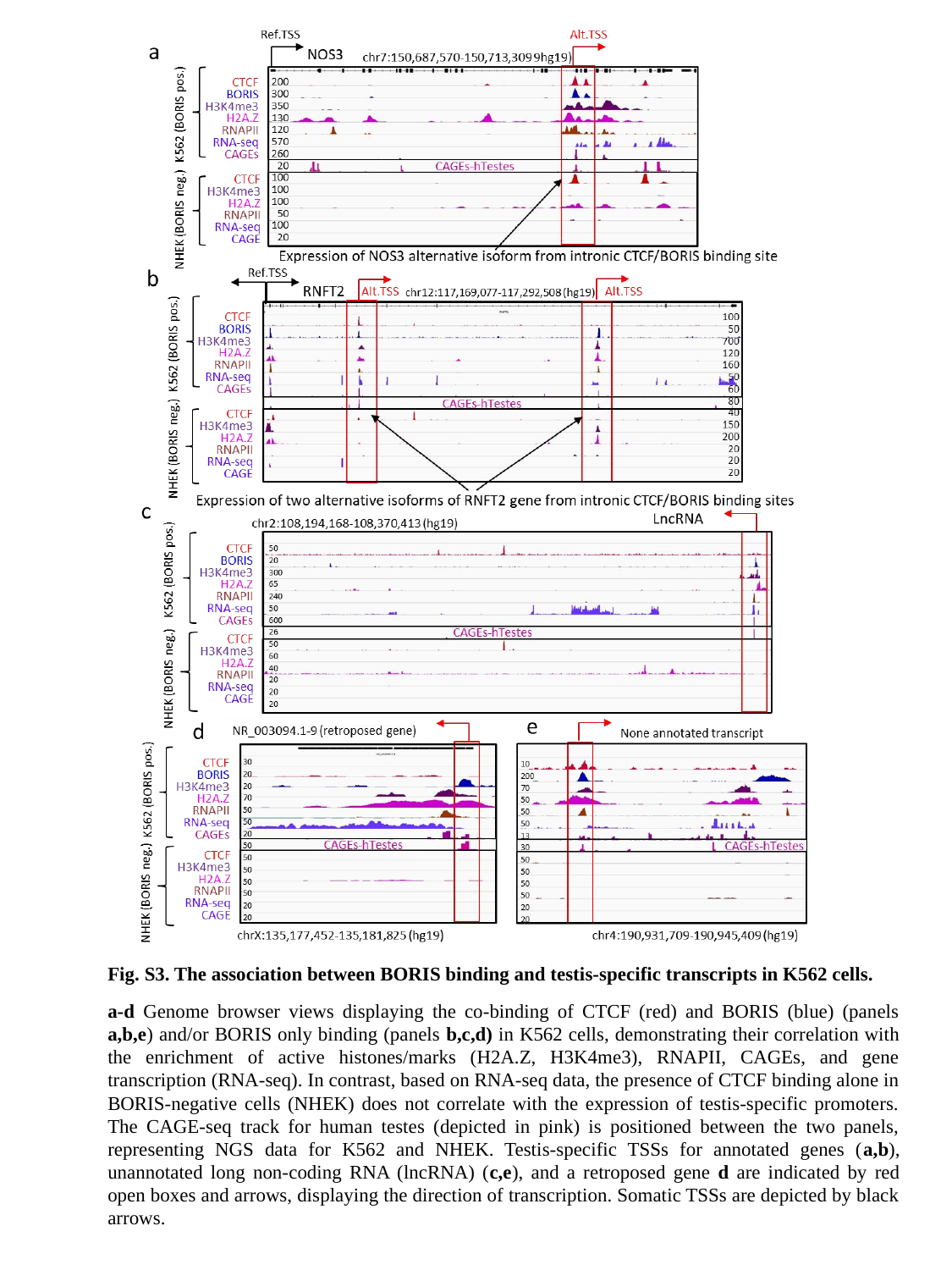

Fig. S3. The association between BORIS binding and testis-specific transcripts in K562 cells.
a-d Genome browser views displaying the co-binding of CTCF (red) and BORIS (blue) (panels a,b,e) and/or BORIS only binding (panels b,c,d) in K562 cells, demonstrating their correlation with the enrichment of active histones/marks (H2A.Z, H3K4me3), RNAPII, CAGEs, and gene transcription (RNA-seq). In contrast, based on RNA-seq data, the presence of CTCF binding alone in BORIS-negative cells (NHEK) does not correlate with the expression of testis-specific promoters. The CAGE-seq track for human testes (depicted in pink) is positioned between the two panels, representing NGS data for K562 and NHEK. Testis-specific TSSs for annotated genes (a,b), unannotated long non-coding RNA (lncRNA) (c,e), and a retroposed gene d are indicated by red open boxes and arrows, displaying the direction of transcription. Somatic TSSs are depicted by black arrows.

## Slide 6
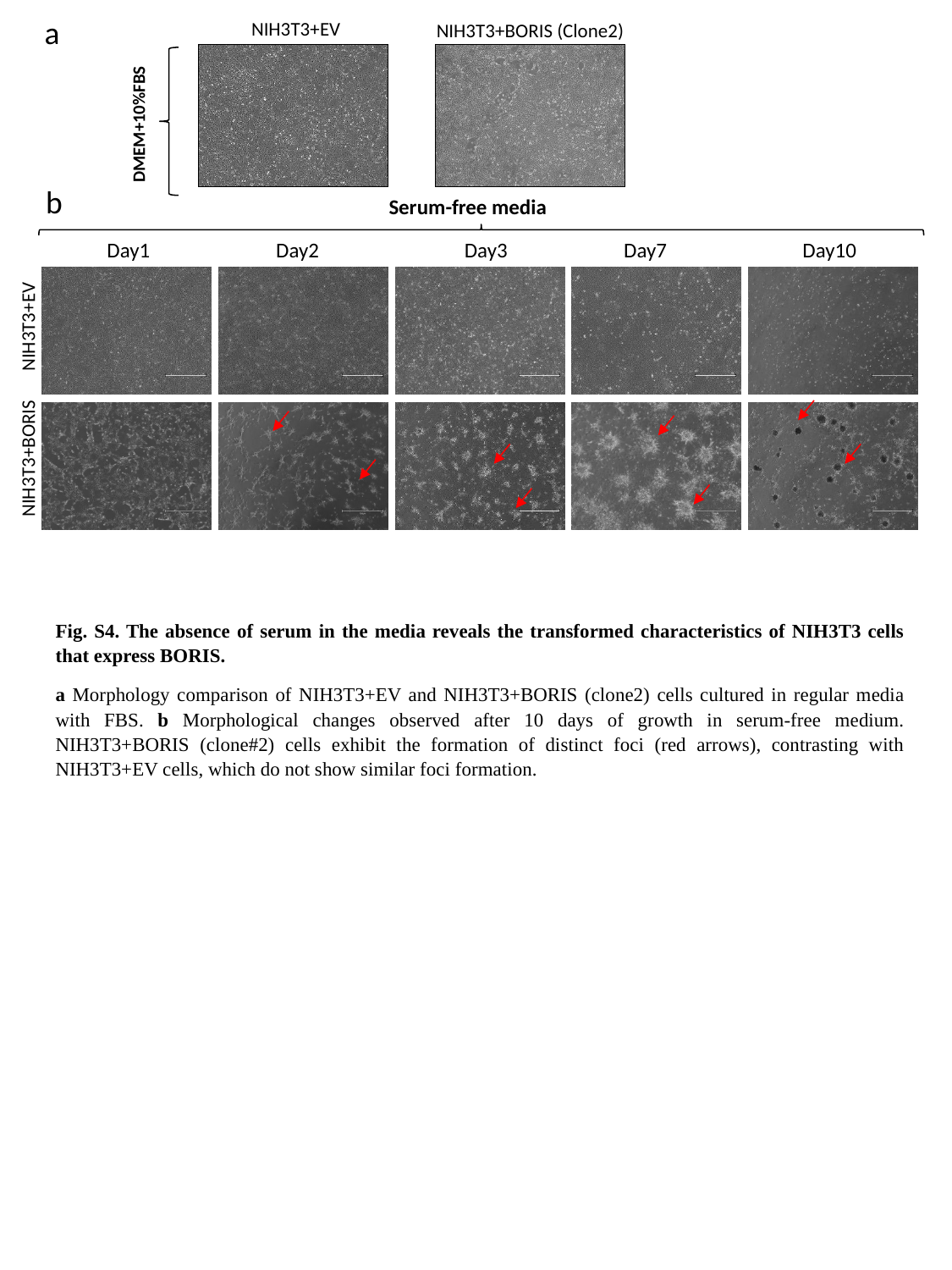

a
NIH3T3+EV
NIH3T3+BORIS (Clone2)
DMEM+10%FBS
b
Serum-free media
Day1 Day2 Day3 Day7 Day10
NIH3T3+EV
NIH3T3+BORIS
Fig. S4. The absence of serum in the media reveals the transformed characteristics of NIH3T3 cells that express BORIS.
a Morphology comparison of NIH3T3+EV and NIH3T3+BORIS (clone2) cells cultured in regular media with FBS. b Morphological changes observed after 10 days of growth in serum-free medium. NIH3T3+BORIS (clone#2) cells exhibit the formation of distinct foci (red arrows), contrasting with NIH3T3+EV cells, which do not show similar foci formation.

## Slide 7
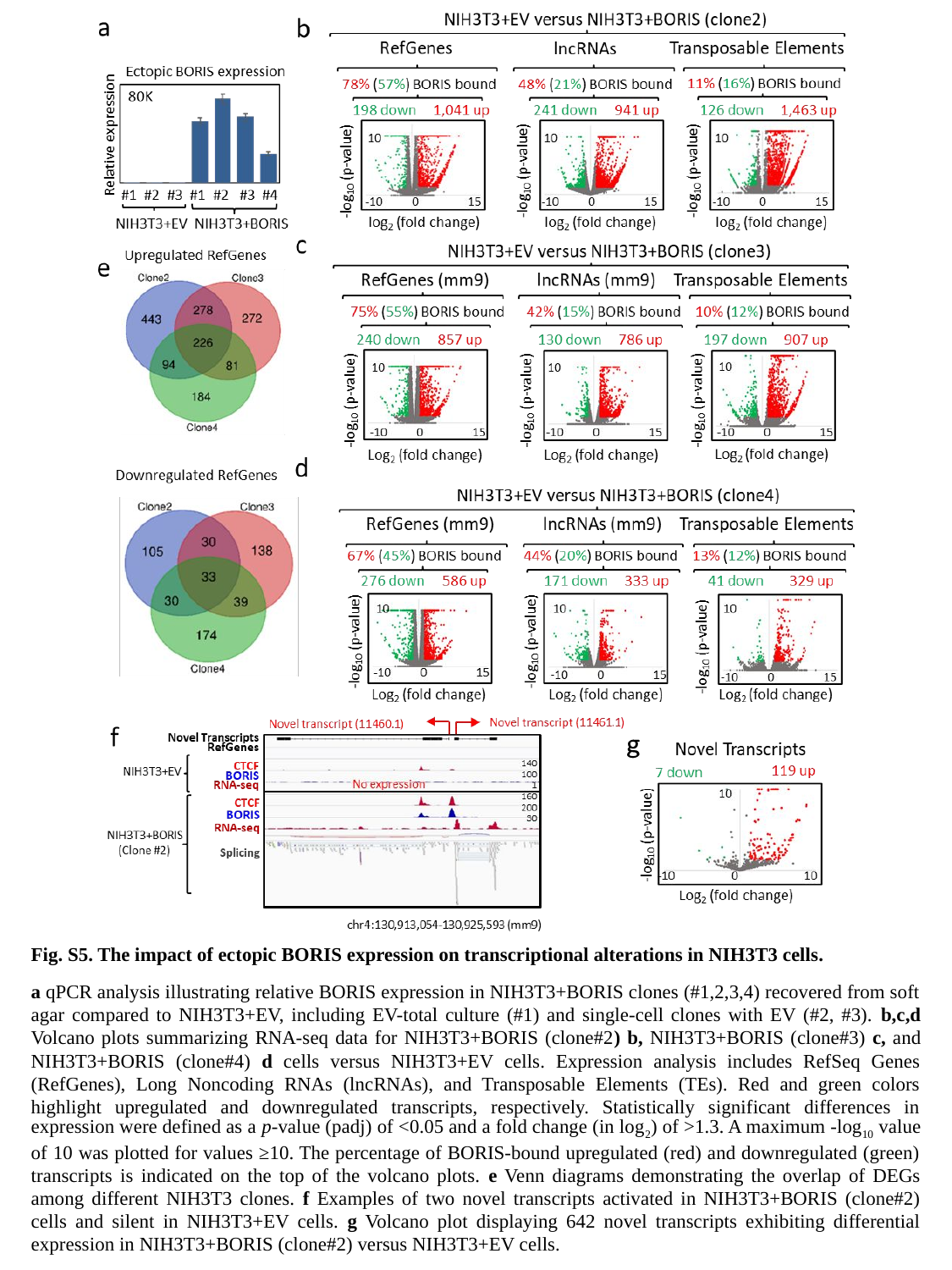

Fig. S5. The impact of ectopic BORIS expression on transcriptional alterations in NIH3T3 cells.
a qPCR analysis illustrating relative BORIS expression in NIH3T3+BORIS clones (#1,2,3,4) recovered from soft agar compared to NIH3T3+EV, including EV-total culture (#1) and single-cell clones with EV (#2, #3). b,c,d Volcano plots summarizing RNA-seq data for NIH3T3+BORIS (clone#2) b, NIH3T3+BORIS (clone#3) c, and NIH3T3+BORIS (clone#4) d cells versus NIH3T3+EV cells. Expression analysis includes RefSeq Genes (RefGenes), Long Noncoding RNAs (lncRNAs), and Transposable Elements (TEs). Red and green colors highlight upregulated and downregulated transcripts, respectively. Statistically significant differences in expression were defined as a p-value (padj) of <0.05 and a fold change (in log2) of >1.3. A maximum -log10 value of 10 was plotted for values ≥10. The percentage of BORIS-bound upregulated (red) and downregulated (green) transcripts is indicated on the top of the volcano plots. e Venn diagrams demonstrating the overlap of DEGs among different NIH3T3 clones. f Examples of two novel transcripts activated in NIH3T3+BORIS (clone#2) cells and silent in NIH3T3+EV cells. g Volcano plot displaying 642 novel transcripts exhibiting differential expression in NIH3T3+BORIS (clone#2) versus NIH3T3+EV cells.

## Slide 8
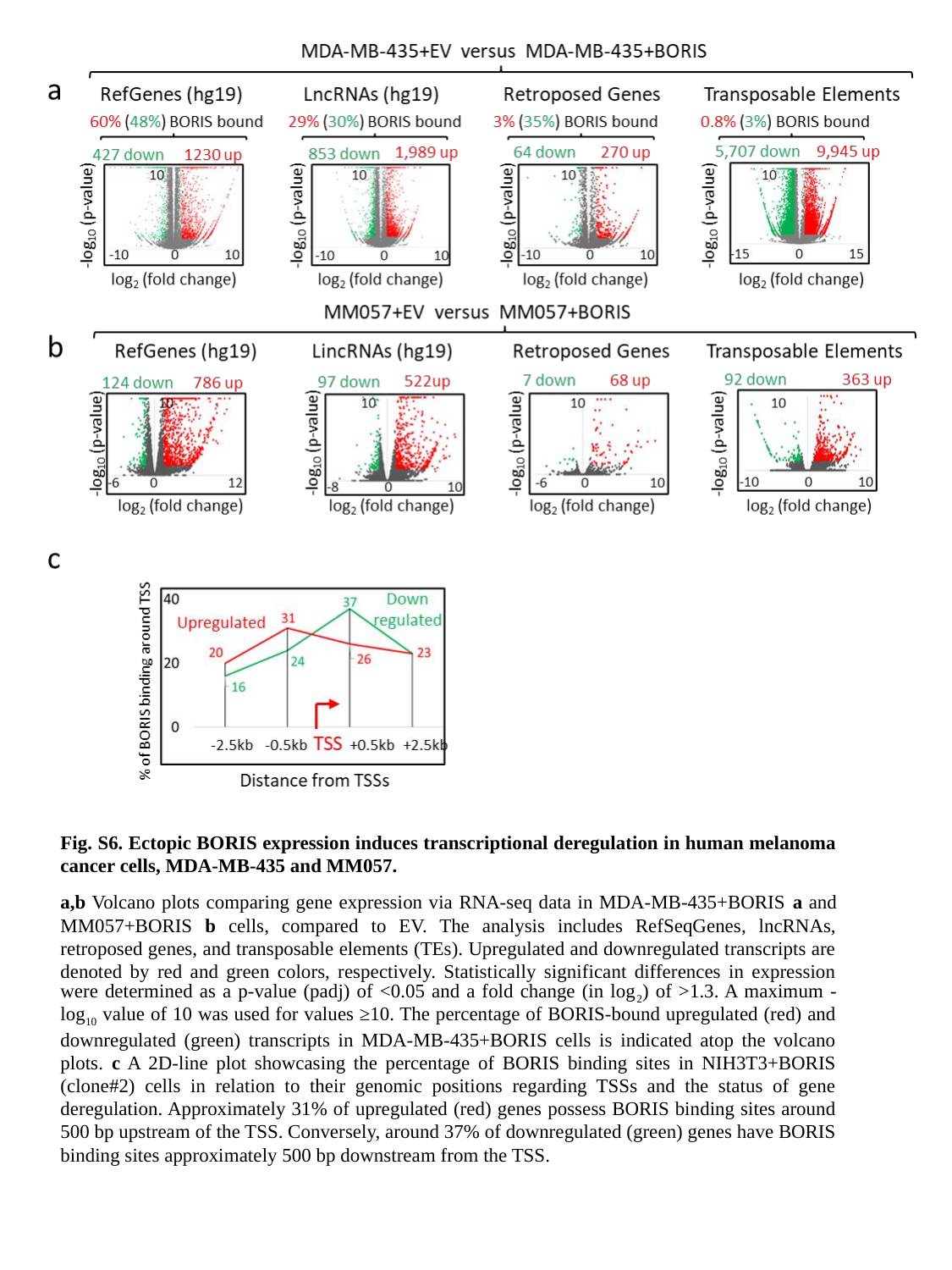

Fig. S6. Ectopic BORIS expression induces transcriptional deregulation in human melanoma cancer cells, MDA-MB-435 and MM057.
a,b Volcano plots comparing gene expression via RNA-seq data in MDA-MB-435+BORIS a and MM057+BORIS b cells, compared to EV. The analysis includes RefSeqGenes, lncRNAs, retroposed genes, and transposable elements (TEs). Upregulated and downregulated transcripts are denoted by red and green colors, respectively. Statistically significant differences in expression were determined as a p-value (padj) of <0.05 and a fold change (in log2) of >1.3. A maximum -log10 value of 10 was used for values ≥10. The percentage of BORIS-bound upregulated (red) and downregulated (green) transcripts in MDA-MB-435+BORIS cells is indicated atop the volcano plots. c A 2D-line plot showcasing the percentage of BORIS binding sites in NIH3T3+BORIS (clone#2) cells in relation to their genomic positions regarding TSSs and the status of gene deregulation. Approximately 31% of upregulated (red) genes possess BORIS binding sites around 500 bp upstream of the TSS. Conversely, around 37% of downregulated (green) genes have BORIS binding sites approximately 500 bp downstream from the TSS.

## Slide 9
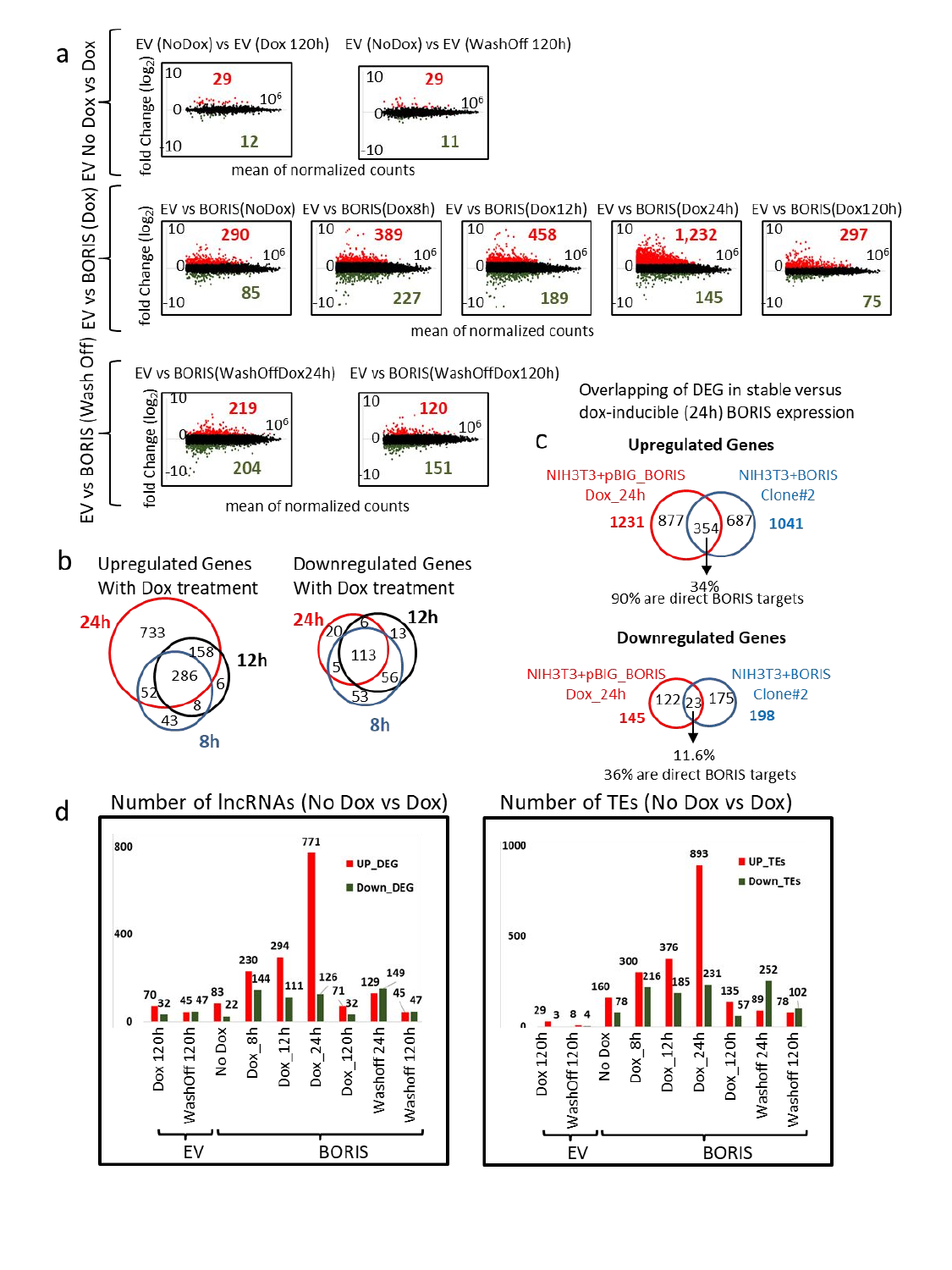

## Slide 10
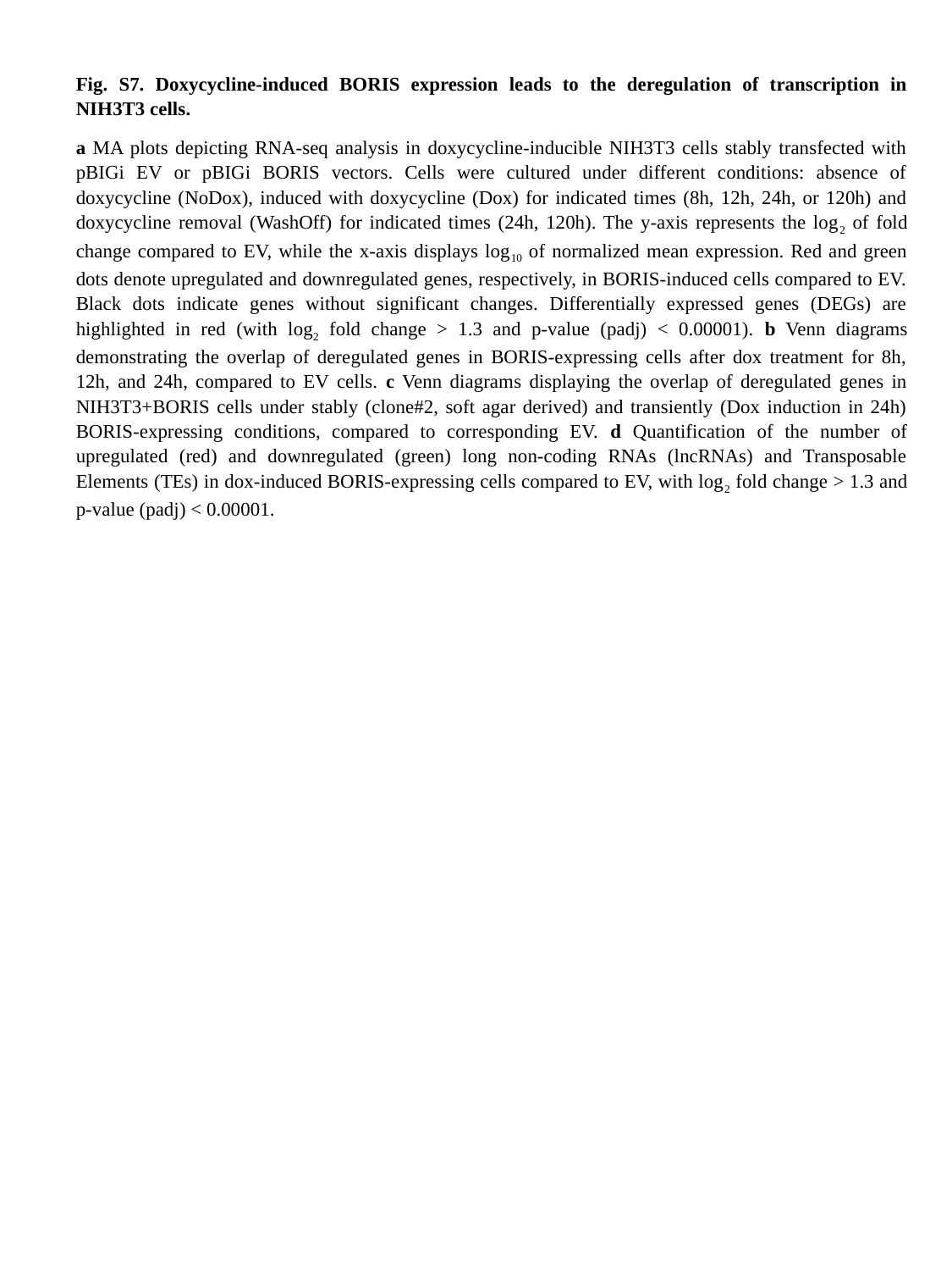

Fig. S7. Doxycycline-induced BORIS expression leads to the deregulation of transcription in NIH3T3 cells.
a MA plots depicting RNA-seq analysis in doxycycline-inducible NIH3T3 cells stably transfected with pBIGi EV or pBIGi BORIS vectors. Cells were cultured under different conditions: absence of doxycycline (NoDox), induced with doxycycline (Dox) for indicated times (8h, 12h, 24h, or 120h) and doxycycline removal (WashOff) for indicated times (24h, 120h). The y-axis represents the log2 of fold change compared to EV, while the x-axis displays log10 of normalized mean expression. Red and green dots denote upregulated and downregulated genes, respectively, in BORIS-induced cells compared to EV. Black dots indicate genes without significant changes. Differentially expressed genes (DEGs) are highlighted in red (with log2 fold change > 1.3 and p-value (padj) < 0.00001). b Venn diagrams demonstrating the overlap of deregulated genes in BORIS-expressing cells after dox treatment for 8h, 12h, and 24h, compared to EV cells. c Venn diagrams displaying the overlap of deregulated genes in NIH3T3+BORIS cells under stably (clone#2, soft agar derived) and transiently (Dox induction in 24h) BORIS-expressing conditions, compared to corresponding EV. d Quantification of the number of upregulated (red) and downregulated (green) long non-coding RNAs (lncRNAs) and Transposable Elements (TEs) in dox-induced BORIS-expressing cells compared to EV, with log2 fold change > 1.3 and p-value (padj) < 0.00001.

## Slide 11
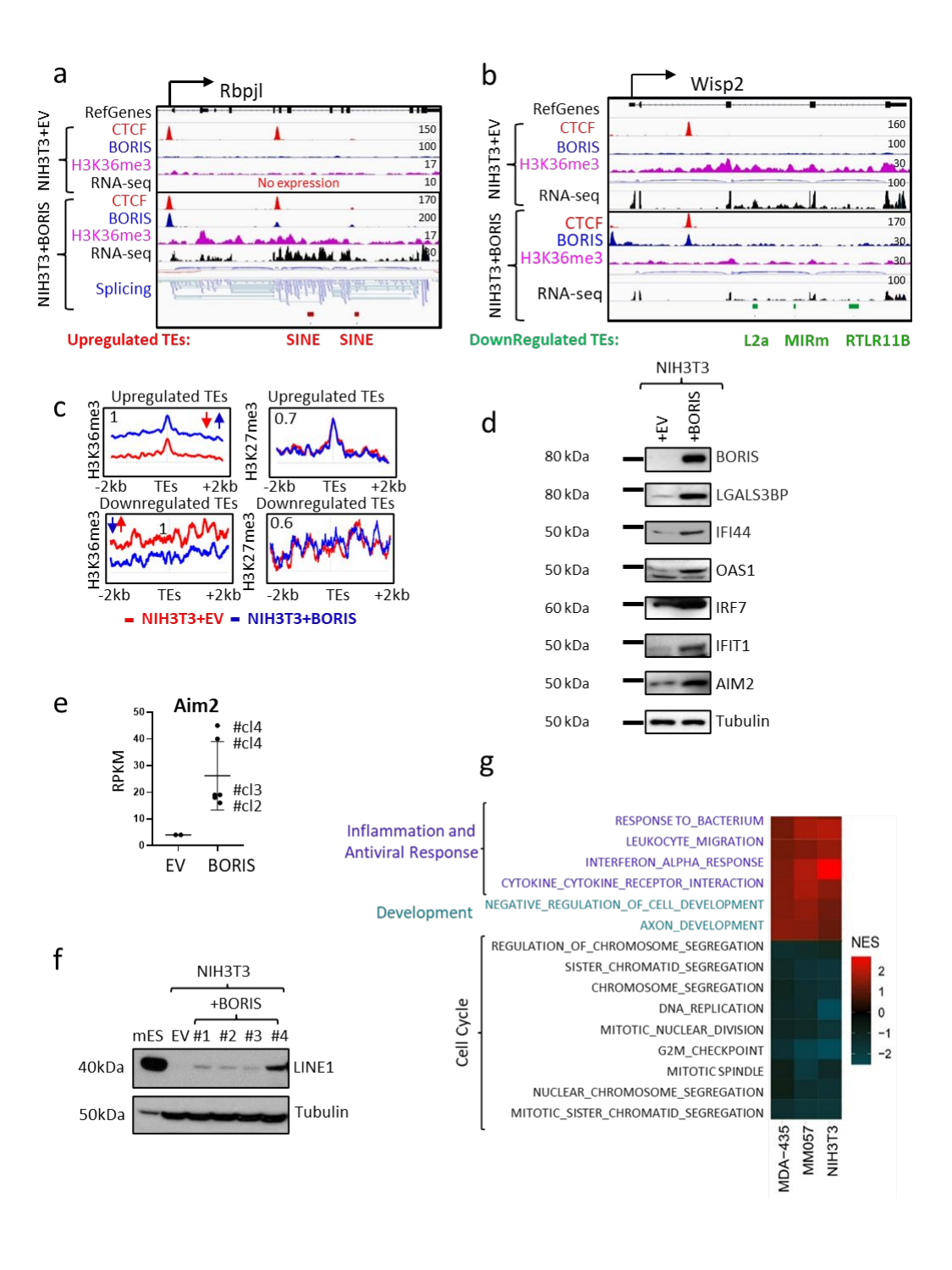

## Slide 12
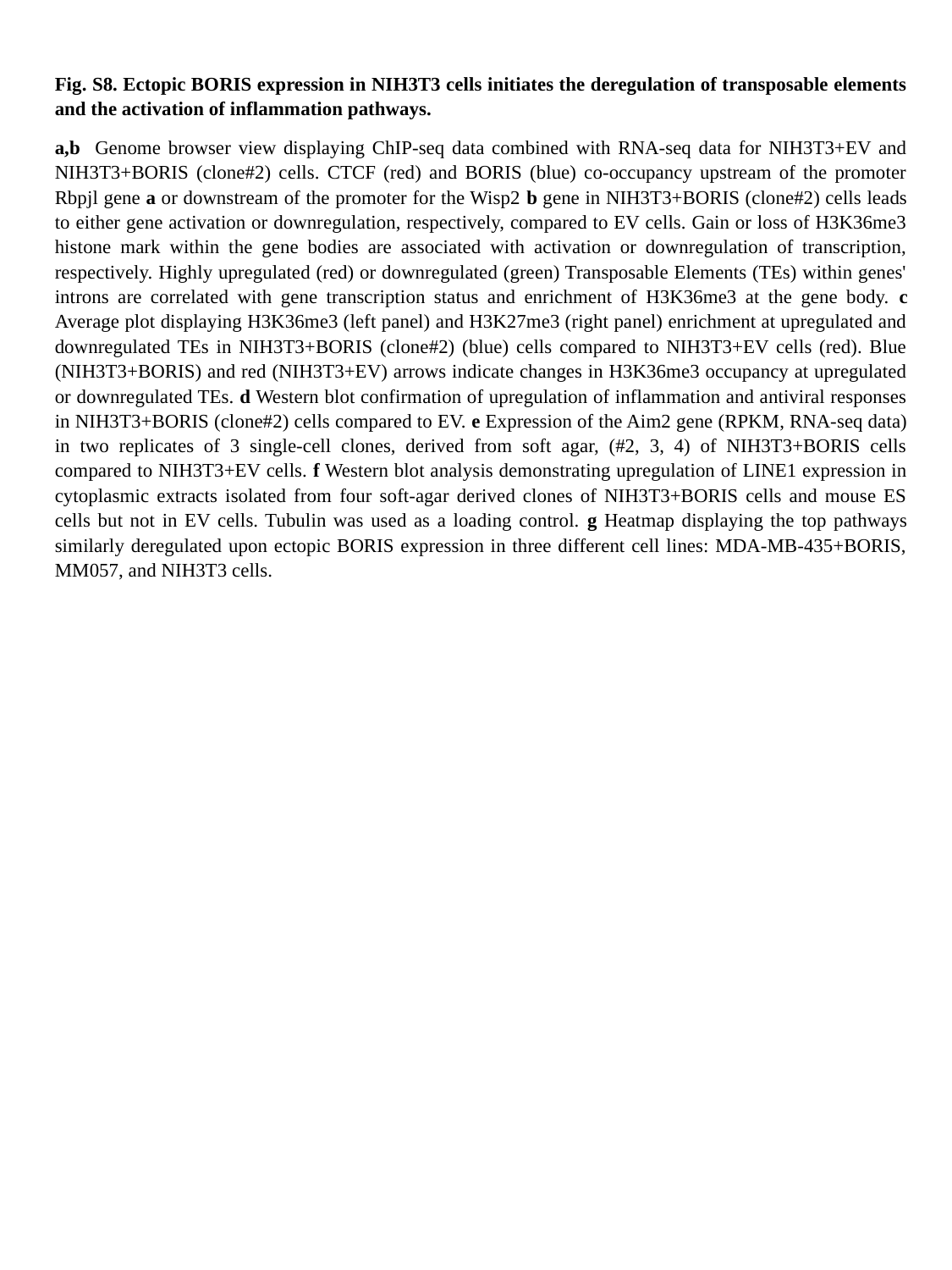

Fig. S8. Ectopic BORIS expression in NIH3T3 cells initiates the deregulation of transposable elements and the activation of inflammation pathways.
a,b Genome browser view displaying ChIP-seq data combined with RNA-seq data for NIH3T3+EV and NIH3T3+BORIS (clone#2) cells. CTCF (red) and BORIS (blue) co-occupancy upstream of the promoter Rbpjl gene a or downstream of the promoter for the Wisp2 b gene in NIH3T3+BORIS (clone#2) cells leads to either gene activation or downregulation, respectively, compared to EV cells. Gain or loss of H3K36me3 histone mark within the gene bodies are associated with activation or downregulation of transcription, respectively. Highly upregulated (red) or downregulated (green) Transposable Elements (TEs) within genes' introns are correlated with gene transcription status and enrichment of H3K36me3 at the gene body. c Average plot displaying H3K36me3 (left panel) and H3K27me3 (right panel) enrichment at upregulated and downregulated TEs in NIH3T3+BORIS (clone#2) (blue) cells compared to NIH3T3+EV cells (red). Blue (NIH3T3+BORIS) and red (NIH3T3+EV) arrows indicate changes in H3K36me3 occupancy at upregulated or downregulated TEs. d Western blot confirmation of upregulation of inflammation and antiviral responses in NIH3T3+BORIS (clone#2) cells compared to EV. e Expression of the Aim2 gene (RPKM, RNA-seq data) in two replicates of 3 single-cell clones, derived from soft agar, (#2, 3, 4) of NIH3T3+BORIS cells compared to NIH3T3+EV cells. f Western blot analysis demonstrating upregulation of LINE1 expression in cytoplasmic extracts isolated from four soft-agar derived clones of NIH3T3+BORIS cells and mouse ES cells but not in EV cells. Tubulin was used as a loading control. g Heatmap displaying the top pathways similarly deregulated upon ectopic BORIS expression in three different cell lines: MDA-MB-435+BORIS, MM057, and NIH3T3 cells.

## Slide 13
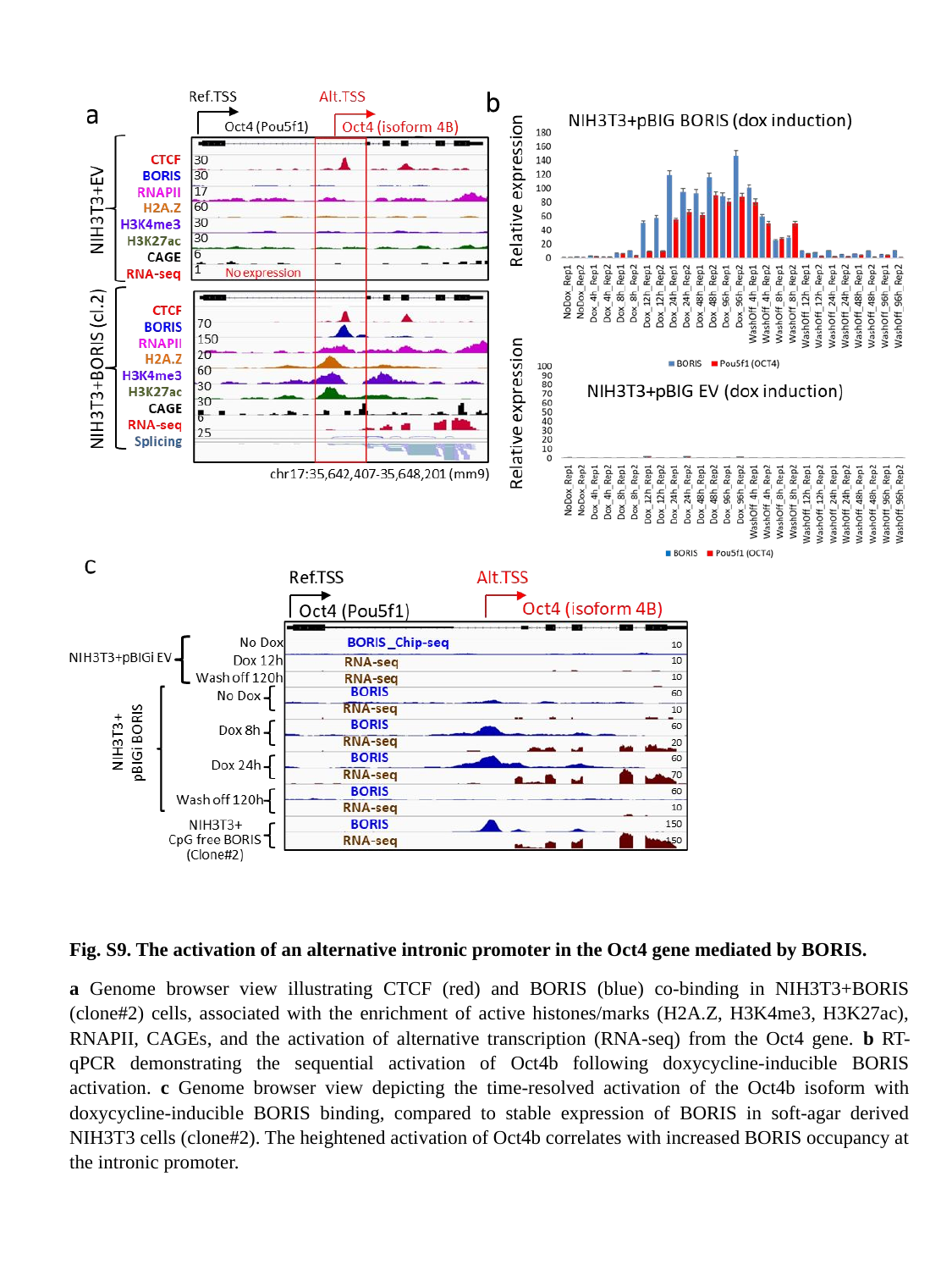

Fig. S9. The activation of an alternative intronic promoter in the Oct4 gene mediated by BORIS.
a Genome browser view illustrating CTCF (red) and BORIS (blue) co-binding in NIH3T3+BORIS (clone#2) cells, associated with the enrichment of active histones/marks (H2A.Z, H3K4me3, H3K27ac), RNAPII, CAGEs, and the activation of alternative transcription (RNA-seq) from the Oct4 gene. b RT-qPCR demonstrating the sequential activation of Oct4b following doxycycline-inducible BORIS activation. c Genome browser view depicting the time-resolved activation of the Oct4b isoform with doxycycline-inducible BORIS binding, compared to stable expression of BORIS in soft-agar derived NIH3T3 cells (clone#2). The heightened activation of Oct4b correlates with increased BORIS occupancy at the intronic promoter.

## Slide 14
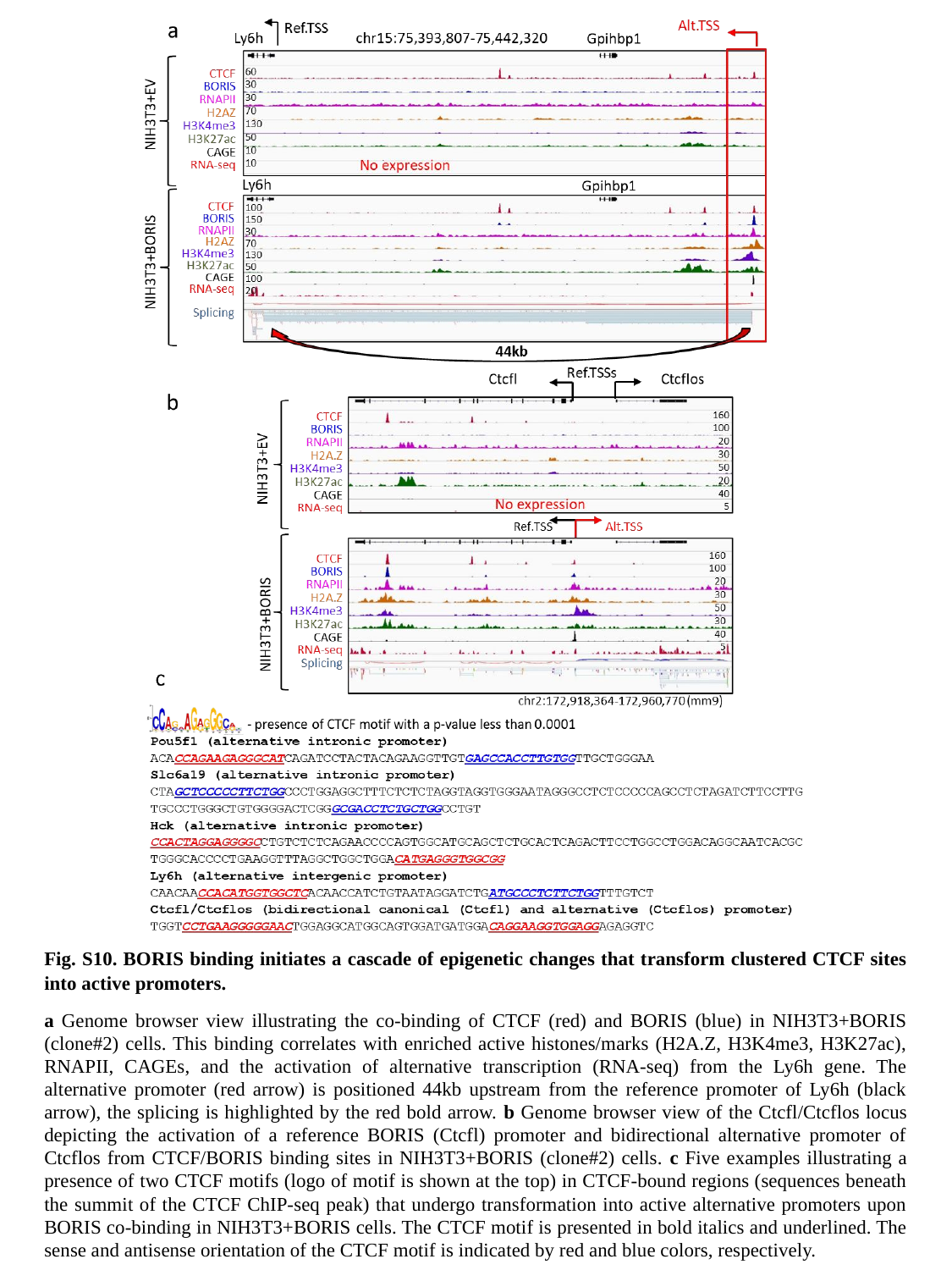

Fig. S10. BORIS binding initiates a cascade of epigenetic changes that transform clustered CTCF sites into active promoters.
a Genome browser view illustrating the co-binding of CTCF (red) and BORIS (blue) in NIH3T3+BORIS (clone#2) cells. This binding correlates with enriched active histones/marks (H2A.Z, H3K4me3, H3K27ac), RNAPII, CAGEs, and the activation of alternative transcription (RNA-seq) from the Ly6h gene. The alternative promoter (red arrow) is positioned 44kb upstream from the reference promoter of Ly6h (black arrow), the splicing is highlighted by the red bold arrow. b Genome browser view of the Ctcfl/Ctcflos locus depicting the activation of a reference BORIS (Ctcfl) promoter and bidirectional alternative promoter of Ctcflos from CTCF/BORIS binding sites in NIH3T3+BORIS (clone#2) cells. c Five examples illustrating a presence of two CTCF motifs (logo of motif is shown at the top) in CTCF-bound regions (sequences beneath the summit of the CTCF ChIP-seq peak) that undergo transformation into active alternative promoters upon BORIS co-binding in NIH3T3+BORIS cells. The CTCF motif is presented in bold italics and underlined. The sense and antisense orientation of the CTCF motif is indicated by red and blue colors, respectively.

## Slide 15
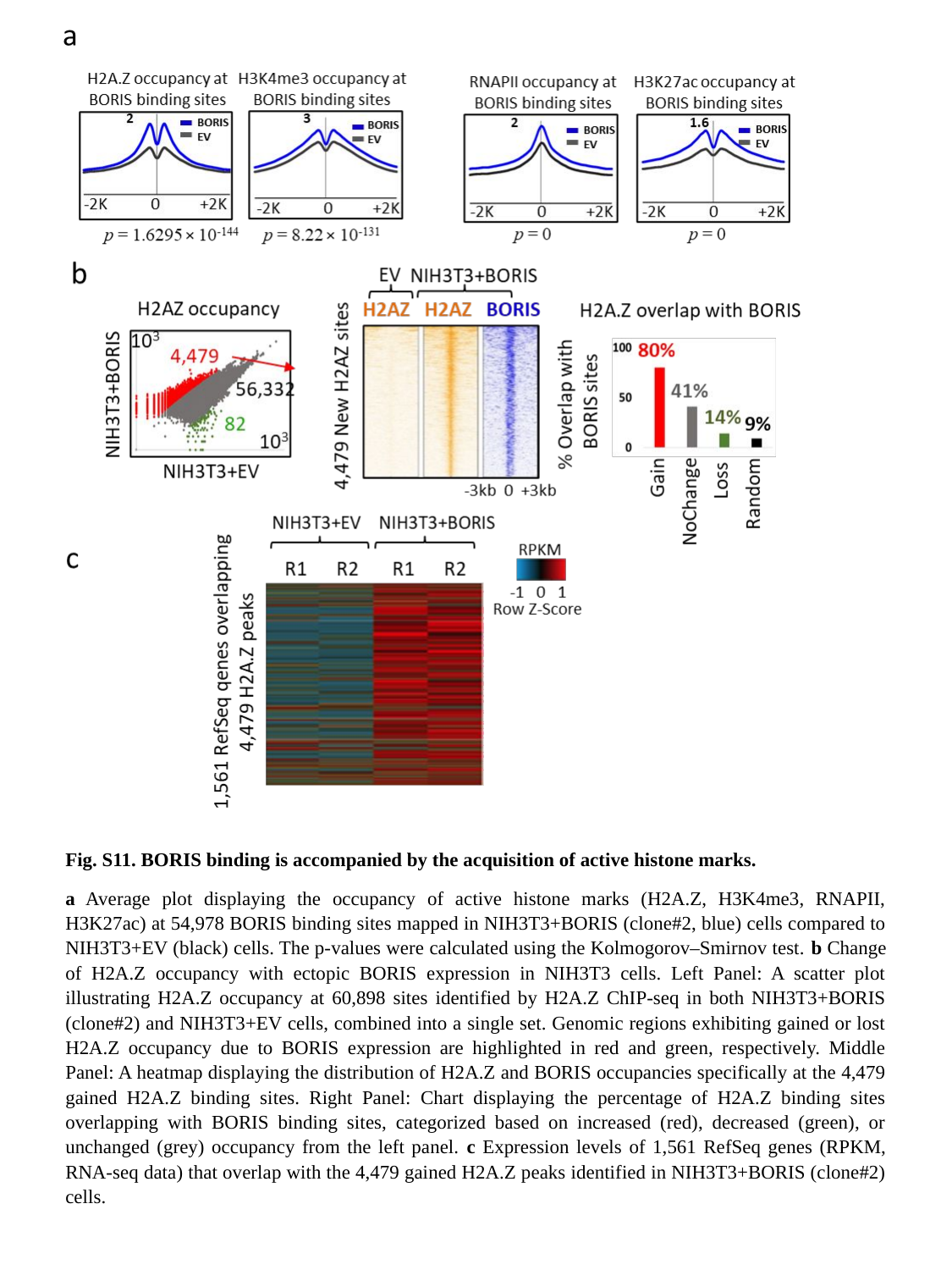

Fig. S11. BORIS binding is accompanied by the acquisition of active histone marks.
a Average plot displaying the occupancy of active histone marks (H2A.Z, H3K4me3, RNAPII, H3K27ac) at 54,978 BORIS binding sites mapped in NIH3T3+BORIS (clone#2, blue) cells compared to NIH3T3+EV (black) cells. The p-values were calculated using the Kolmogorov–Smirnov test. b Change of H2A.Z occupancy with ectopic BORIS expression in NIH3T3 cells. Left Panel: A scatter plot illustrating H2A.Z occupancy at 60,898 sites identified by H2A.Z ChIP-seq in both NIH3T3+BORIS (clone#2) and NIH3T3+EV cells, combined into a single set. Genomic regions exhibiting gained or lost H2A.Z occupancy due to BORIS expression are highlighted in red and green, respectively. Middle Panel: A heatmap displaying the distribution of H2A.Z and BORIS occupancies specifically at the 4,479 gained H2A.Z binding sites. Right Panel: Chart displaying the percentage of H2A.Z binding sites overlapping with BORIS binding sites, categorized based on increased (red), decreased (green), or unchanged (grey) occupancy from the left panel. c Expression levels of 1,561 RefSeq genes (RPKM, RNA-seq data) that overlap with the 4,479 gained H2A.Z peaks identified in NIH3T3+BORIS (clone#2) cells.

## Slide 16
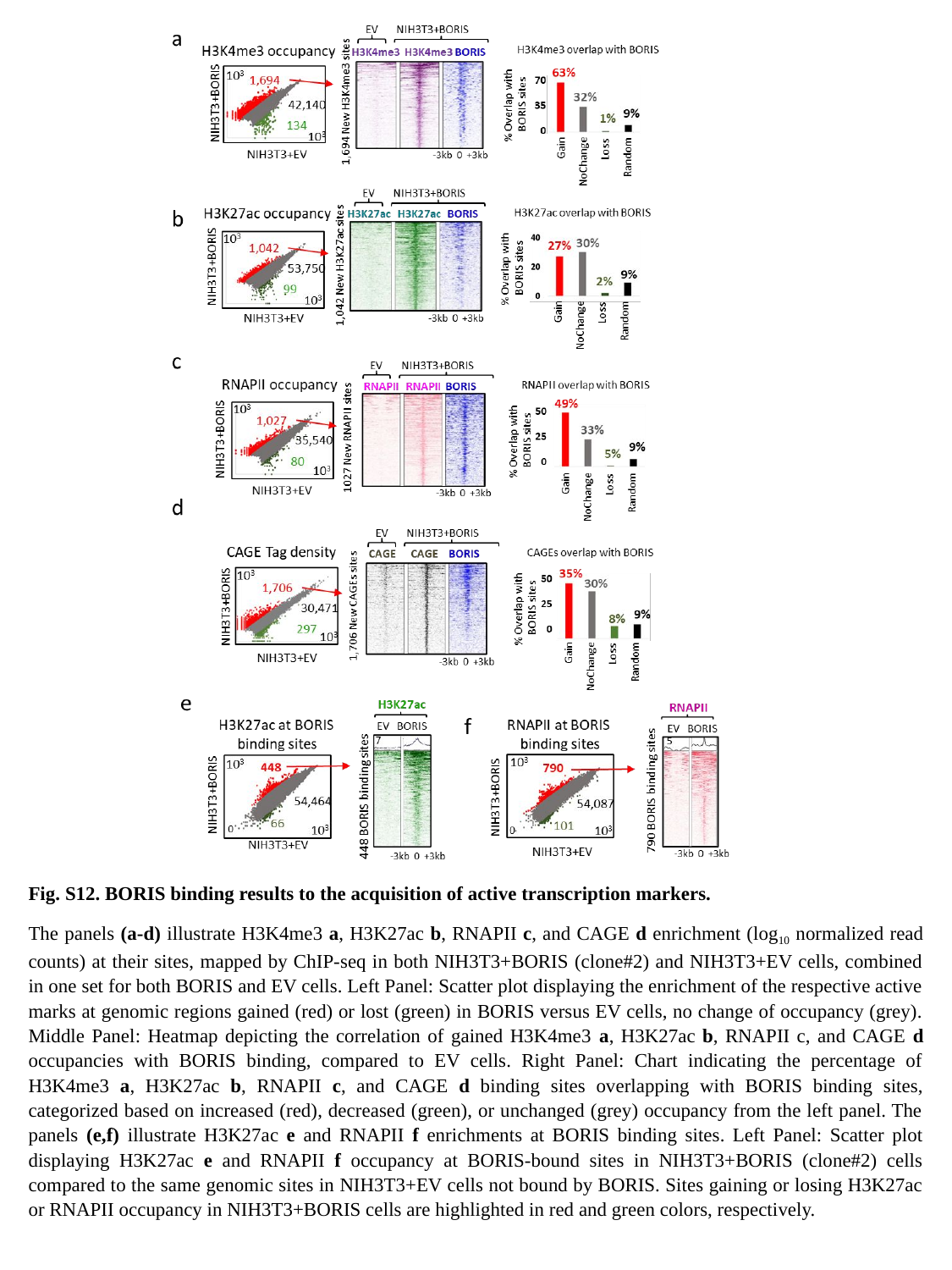

Fig. S12. BORIS binding results to the acquisition of active transcription markers.
The panels (a-d) illustrate H3K4me3 a, H3K27ac b, RNAPII c, and CAGE d enrichment (log10 normalized read counts) at their sites, mapped by ChIP-seq in both NIH3T3+BORIS (clone#2) and NIH3T3+EV cells, combined in one set for both BORIS and EV cells. Left Panel: Scatter plot displaying the enrichment of the respective active marks at genomic regions gained (red) or lost (green) in BORIS versus EV cells, no change of occupancy (grey). Middle Panel: Heatmap depicting the correlation of gained H3K4me3 a, H3K27ac b, RNAPII c, and CAGE d occupancies with BORIS binding, compared to EV cells. Right Panel: Chart indicating the percentage of H3K4me3 a, H3K27ac b, RNAPII c, and CAGE d binding sites overlapping with BORIS binding sites, categorized based on increased (red), decreased (green), or unchanged (grey) occupancy from the left panel. The panels (e,f) illustrate H3K27ac e and RNAPII f enrichments at BORIS binding sites. Left Panel: Scatter plot displaying H3K27ac e and RNAPII f occupancy at BORIS-bound sites in NIH3T3+BORIS (clone#2) cells compared to the same genomic sites in NIH3T3+EV cells not bound by BORIS. Sites gaining or losing H3K27ac or RNAPII occupancy in NIH3T3+BORIS cells are highlighted in red and green colors, respectively.

## Slide 17
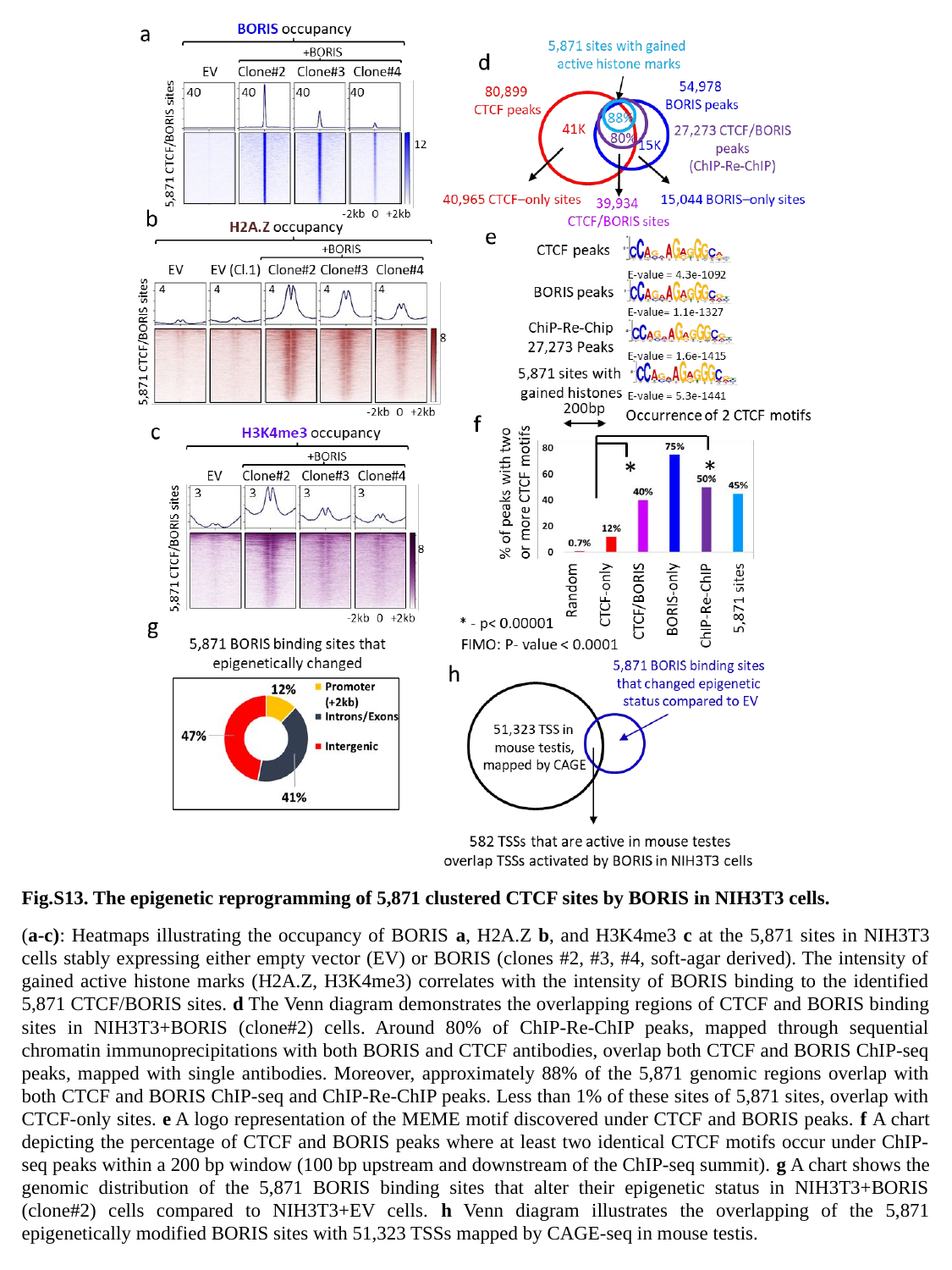

Fig.S13. The epigenetic reprogramming of 5,871 clustered CTCF sites by BORIS in NIH3T3 cells.
(a-c): Heatmaps illustrating the occupancy of BORIS a, H2A.Z b, and H3K4me3 c at the 5,871 sites in NIH3T3 cells stably expressing either empty vector (EV) or BORIS (clones #2, #3, #4, soft-agar derived). The intensity of gained active histone marks (H2A.Z, H3K4me3) correlates with the intensity of BORIS binding to the identified 5,871 CTCF/BORIS sites. d The Venn diagram demonstrates the overlapping regions of CTCF and BORIS binding sites in NIH3T3+BORIS (clone#2) cells. Around 80% of ChIP-Re-ChIP peaks, mapped through sequential chromatin immunoprecipitations with both BORIS and CTCF antibodies, overlap both CTCF and BORIS ChIP-seq peaks, mapped with single antibodies. Moreover, approximately 88% of the 5,871 genomic regions overlap with both CTCF and BORIS ChIP-seq and ChIP-Re-ChIP peaks. Less than 1% of these sites of 5,871 sites, overlap with CTCF-only sites. e A logo representation of the MEME motif discovered under CTCF and BORIS peaks. f A chart depicting the percentage of CTCF and BORIS peaks where at least two identical CTCF motifs occur under ChIP-seq peaks within a 200 bp window (100 bp upstream and downstream of the ChIP-seq summit). g A chart shows the genomic distribution of the 5,871 BORIS binding sites that alter their epigenetic status in NIH3T3+BORIS (clone#2) cells compared to NIH3T3+EV cells. h Venn diagram illustrates the overlapping of the 5,871 epigenetically modified BORIS sites with 51,323 TSSs mapped by CAGE-seq in mouse testis.

## Slide 18
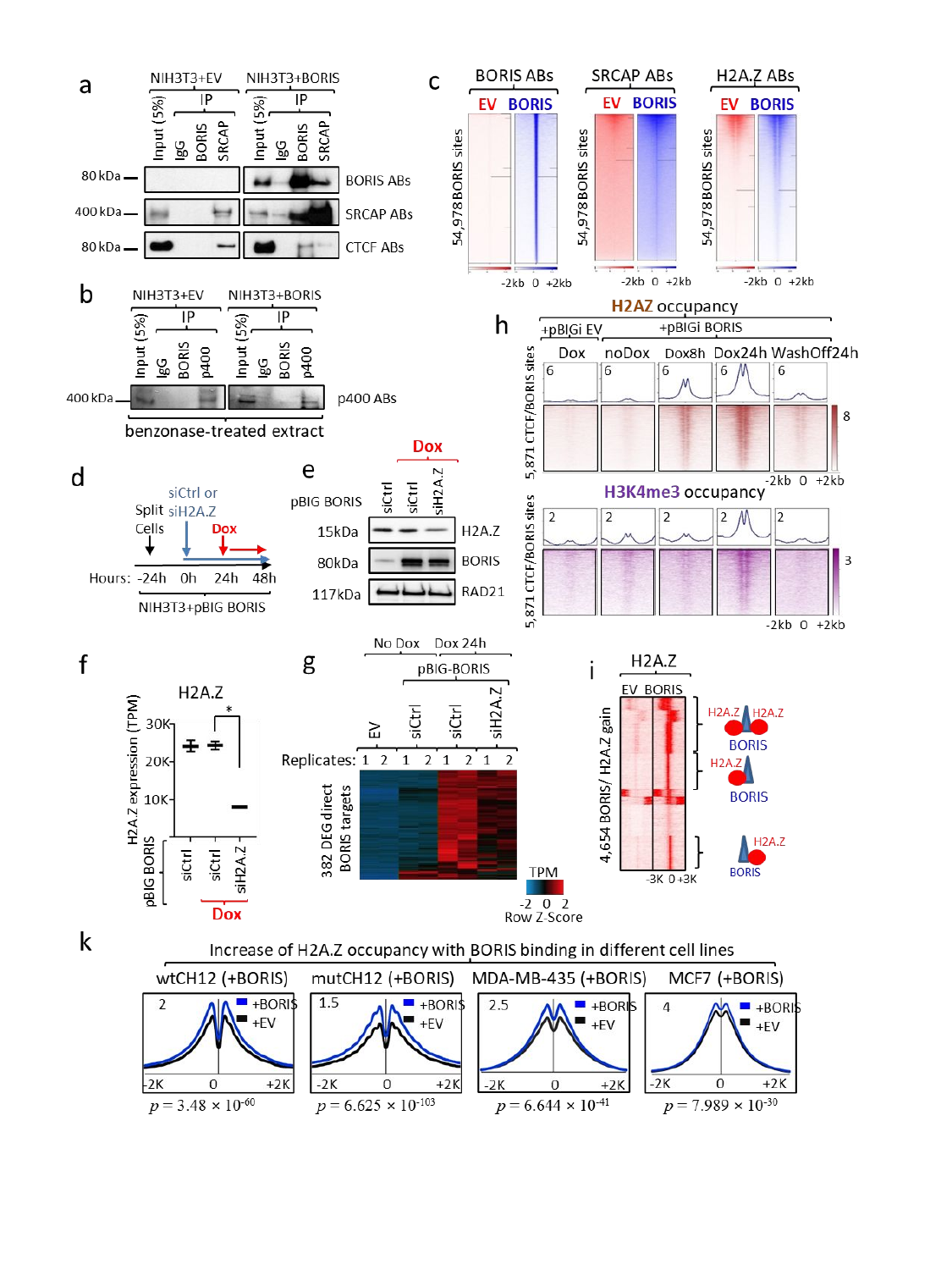

## Slide 19
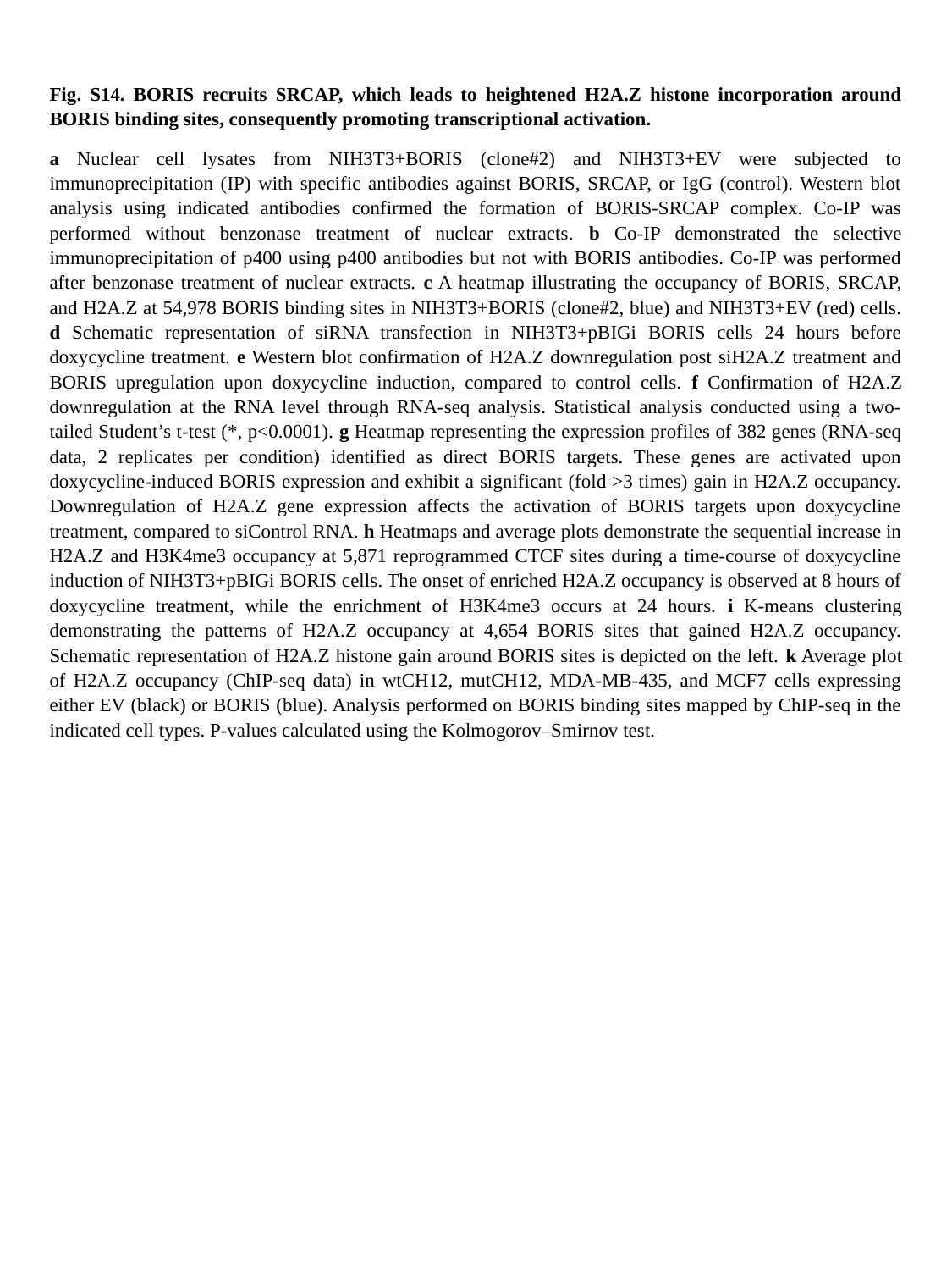

Fig. S14. BORIS recruits SRCAP, which leads to heightened H2A.Z histone incorporation around BORIS binding sites, consequently promoting transcriptional activation.
a Nuclear cell lysates from NIH3T3+BORIS (clone#2) and NIH3T3+EV were subjected to immunoprecipitation (IP) with specific antibodies against BORIS, SRCAP, or IgG (control). Western blot analysis using indicated antibodies confirmed the formation of BORIS-SRCAP complex. Co-IP was performed without benzonase treatment of nuclear extracts. b Co-IP demonstrated the selective immunoprecipitation of p400 using p400 antibodies but not with BORIS antibodies. Co-IP was performed after benzonase treatment of nuclear extracts. c A heatmap illustrating the occupancy of BORIS, SRCAP, and H2A.Z at 54,978 BORIS binding sites in NIH3T3+BORIS (clone#2, blue) and NIH3T3+EV (red) cells. d Schematic representation of siRNA transfection in NIH3T3+pBIGi BORIS cells 24 hours before doxycycline treatment. e Western blot confirmation of H2A.Z downregulation post siH2A.Z treatment and BORIS upregulation upon doxycycline induction, compared to control cells. f Confirmation of H2A.Z downregulation at the RNA level through RNA-seq analysis. Statistical analysis conducted using a two-tailed Student’s t-test (*, p<0.0001). g Heatmap representing the expression profiles of 382 genes (RNA-seq data, 2 replicates per condition) identified as direct BORIS targets. These genes are activated upon doxycycline-induced BORIS expression and exhibit a significant (fold >3 times) gain in H2A.Z occupancy. Downregulation of H2A.Z gene expression affects the activation of BORIS targets upon doxycycline treatment, compared to siControl RNA. h Heatmaps and average plots demonstrate the sequential increase in H2A.Z and H3K4me3 occupancy at 5,871 reprogrammed CTCF sites during a time-course of doxycycline induction of NIH3T3+pBIGi BORIS cells. The onset of enriched H2A.Z occupancy is observed at 8 hours of doxycycline treatment, while the enrichment of H3K4me3 occurs at 24 hours. i K-means clustering demonstrating the patterns of H2A.Z occupancy at 4,654 BORIS sites that gained H2A.Z occupancy. Schematic representation of H2A.Z histone gain around BORIS sites is depicted on the left. k Average plot of H2A.Z occupancy (ChIP-seq data) in wtCH12, mutCH12, MDA-MB-435, and MCF7 cells expressing either EV (black) or BORIS (blue). Analysis performed on BORIS binding sites mapped by ChIP-seq in the indicated cell types. P-values calculated using the Kolmogorov–Smirnov test.

## Slide 20
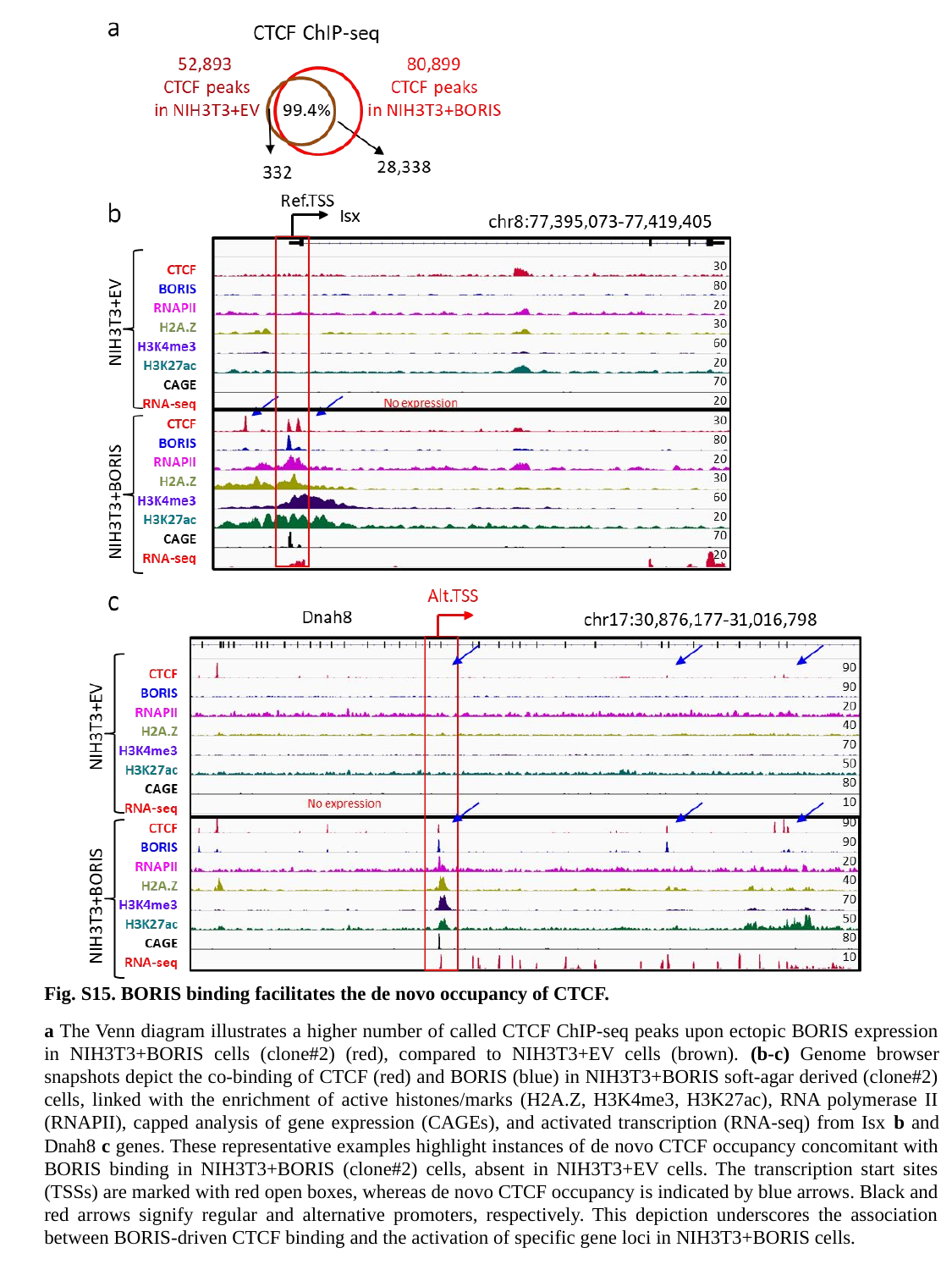

Fig. S15. BORIS binding facilitates the de novo occupancy of CTCF.
a The Venn diagram illustrates a higher number of called CTCF ChIP-seq peaks upon ectopic BORIS expression in NIH3T3+BORIS cells (clone#2) (red), compared to NIH3T3+EV cells (brown). (b-c) Genome browser snapshots depict the co-binding of CTCF (red) and BORIS (blue) in NIH3T3+BORIS soft-agar derived (clone#2) cells, linked with the enrichment of active histones/marks (H2A.Z, H3K4me3, H3K27ac), RNA polymerase II (RNAPII), capped analysis of gene expression (CAGEs), and activated transcription (RNA-seq) from Isx b and Dnah8 c genes. These representative examples highlight instances of de novo CTCF occupancy concomitant with BORIS binding in NIH3T3+BORIS (clone#2) cells, absent in NIH3T3+EV cells. The transcription start sites (TSSs) are marked with red open boxes, whereas de novo CTCF occupancy is indicated by blue arrows. Black and red arrows signify regular and alternative promoters, respectively. This depiction underscores the association between BORIS-driven CTCF binding and the activation of specific gene loci in NIH3T3+BORIS cells.

## Slide 21
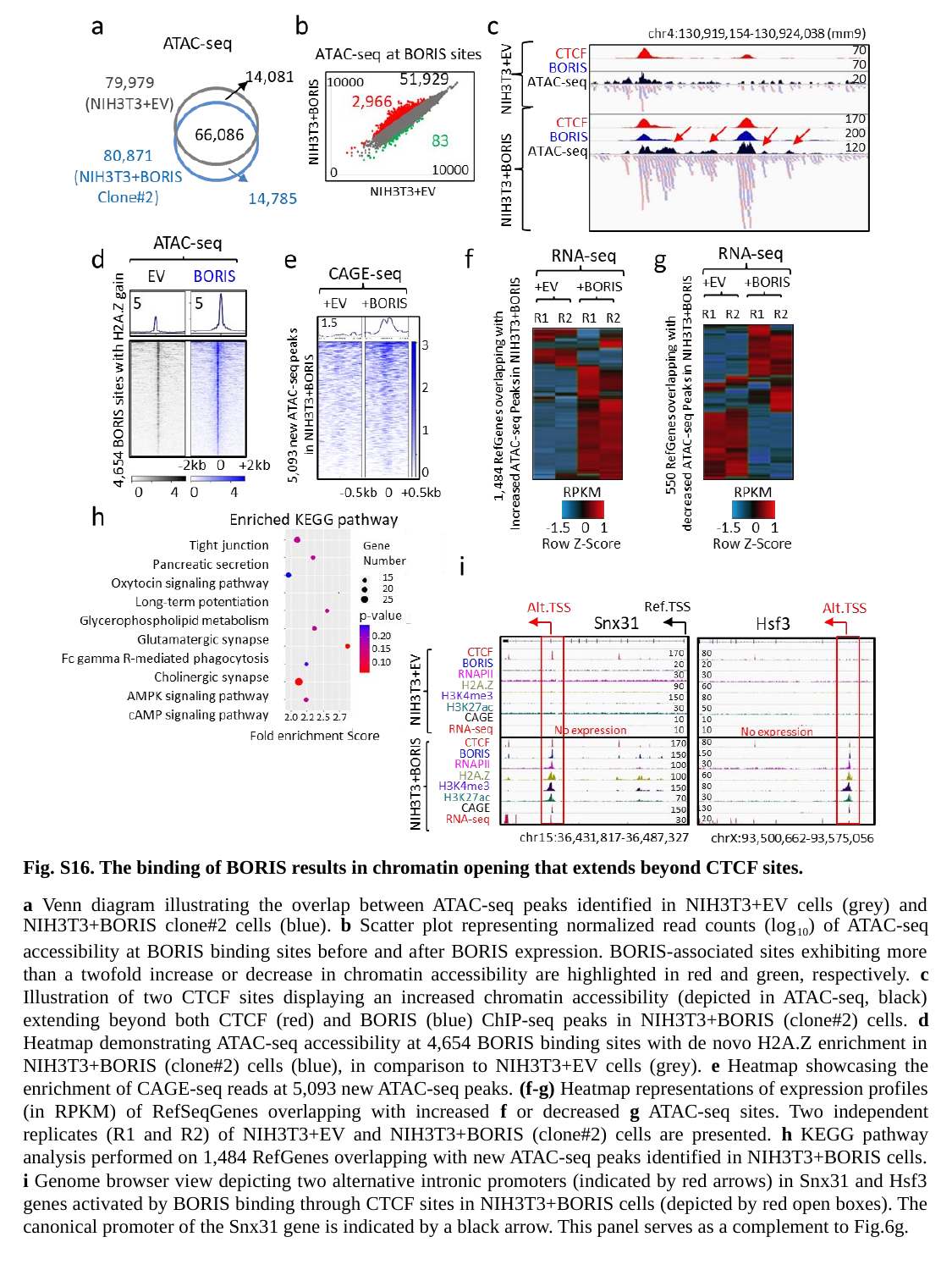

Fig. S16. The binding of BORIS results in chromatin opening that extends beyond CTCF sites.
a Venn diagram illustrating the overlap between ATAC-seq peaks identified in NIH3T3+EV cells (grey) and NIH3T3+BORIS clone#2 cells (blue). b Scatter plot representing normalized read counts (log10) of ATAC-seq accessibility at BORIS binding sites before and after BORIS expression. BORIS-associated sites exhibiting more than a twofold increase or decrease in chromatin accessibility are highlighted in red and green, respectively. c Illustration of two CTCF sites displaying an increased chromatin accessibility (depicted in ATAC-seq, black) extending beyond both CTCF (red) and BORIS (blue) ChIP-seq peaks in NIH3T3+BORIS (clone#2) cells. d Heatmap demonstrating ATAC-seq accessibility at 4,654 BORIS binding sites with de novo H2A.Z enrichment in NIH3T3+BORIS (clone#2) cells (blue), in comparison to NIH3T3+EV cells (grey). e Heatmap showcasing the enrichment of CAGE-seq reads at 5,093 new ATAC-seq peaks. (f-g) Heatmap representations of expression profiles (in RPKM) of RefSeqGenes overlapping with increased f or decreased g ATAC-seq sites. Two independent replicates (R1 and R2) of NIH3T3+EV and NIH3T3+BORIS (clone#2) cells are presented. h KEGG pathway analysis performed on 1,484 RefGenes overlapping with new ATAC-seq peaks identified in NIH3T3+BORIS cells. i Genome browser view depicting two alternative intronic promoters (indicated by red arrows) in Snx31 and Hsf3 genes activated by BORIS binding through CTCF sites in NIH3T3+BORIS cells (depicted by red open boxes). The canonical promoter of the Snx31 gene is indicated by a black arrow. This panel serves as a complement to Fig.6g.

## Slide 22
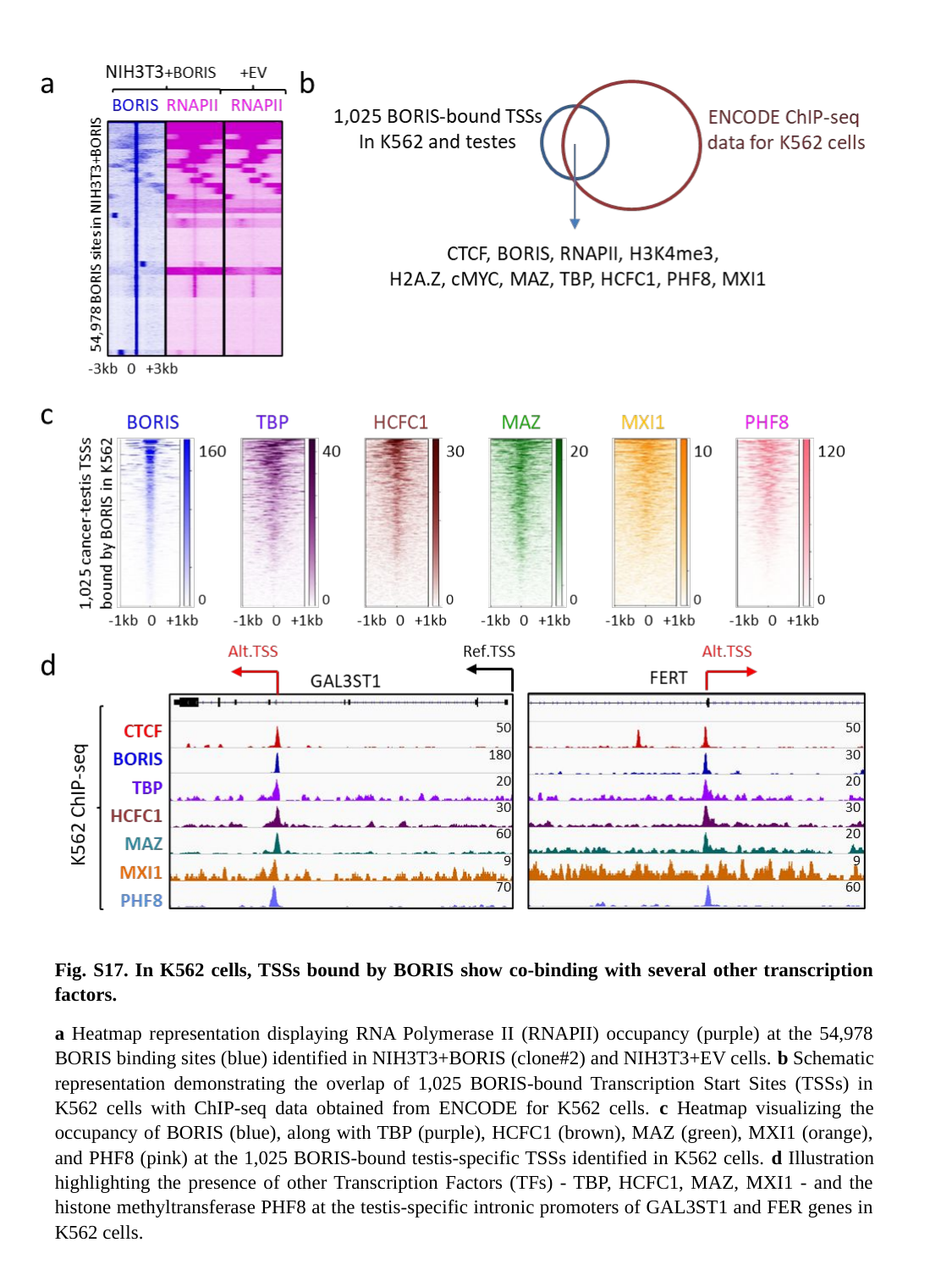

Fig. S17. In K562 cells, TSSs bound by BORIS show co-binding with several other transcription factors.
a Heatmap representation displaying RNA Polymerase II (RNAPII) occupancy (purple) at the 54,978 BORIS binding sites (blue) identified in NIH3T3+BORIS (clone#2) and NIH3T3+EV cells. b Schematic representation demonstrating the overlap of 1,025 BORIS-bound Transcription Start Sites (TSSs) in K562 cells with ChIP-seq data obtained from ENCODE for K562 cells. c Heatmap visualizing the occupancy of BORIS (blue), along with TBP (purple), HCFC1 (brown), MAZ (green), MXI1 (orange), and PHF8 (pink) at the 1,025 BORIS-bound testis-specific TSSs identified in K562 cells. d Illustration highlighting the presence of other Transcription Factors (TFs) - TBP, HCFC1, MAZ, MXI1 - and the histone methyltransferase PHF8 at the testis-specific intronic promoters of GAL3ST1 and FER genes in K562 cells.

## Slide 23
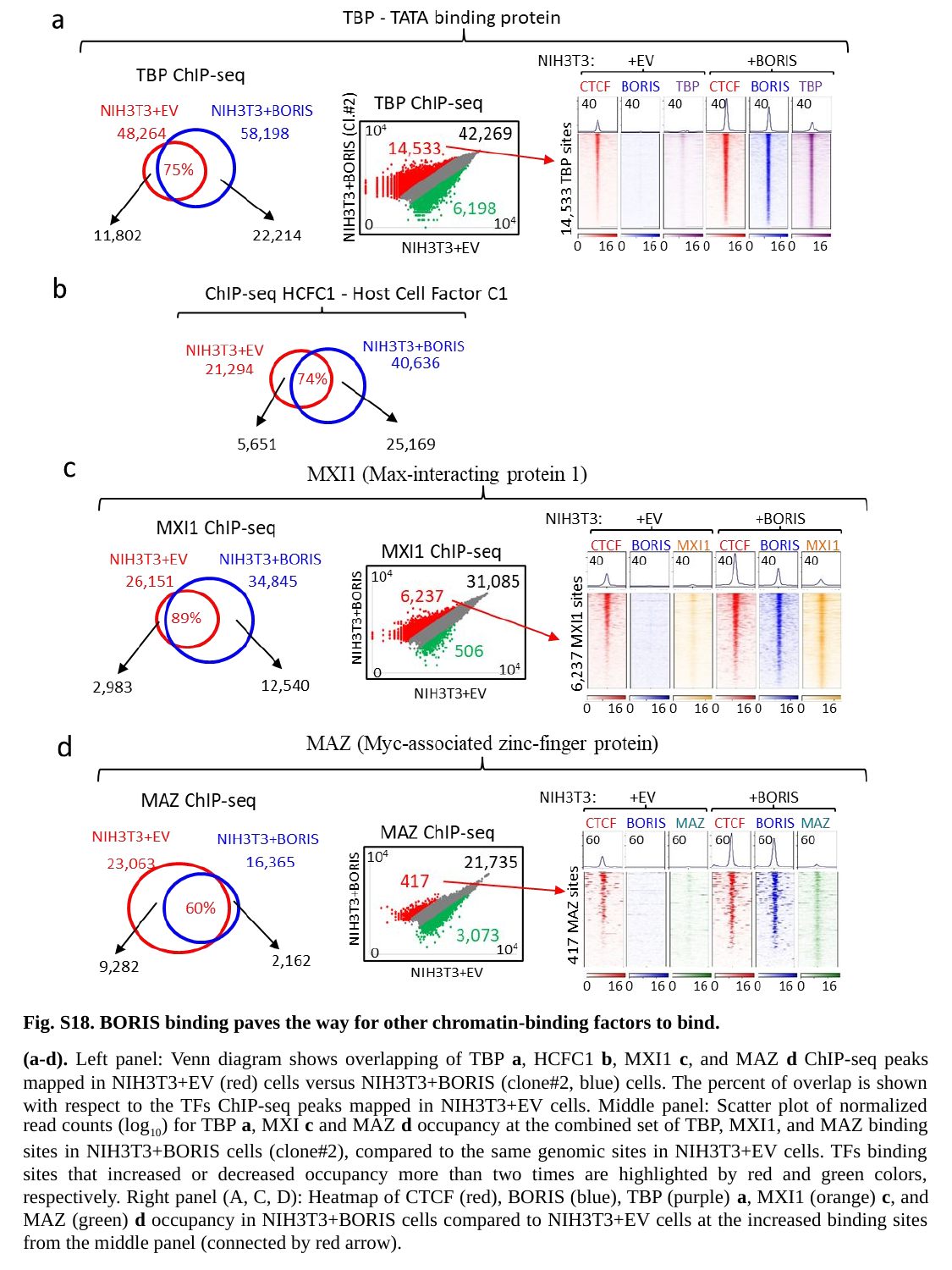

Fig. S18. BORIS binding paves the way for other chromatin-binding factors to bind.
(a-d). Left panel: Venn diagram shows overlapping of TBP a, HCFC1 b, MXI1 c, and MAZ d ChIP-seq peaks mapped in NIH3T3+EV (red) cells versus NIH3T3+BORIS (clone#2, blue) cells. The percent of overlap is shown with respect to the TFs ChIP-seq peaks mapped in NIH3T3+EV cells. Middle panel: Scatter plot of normalized read counts (log10) for TBP a, MXI c and MAZ d occupancy at the combined set of TBP, MXI1, and MAZ binding sites in NIH3T3+BORIS cells (clone#2), compared to the same genomic sites in NIH3T3+EV cells. TFs binding sites that increased or decreased occupancy more than two times are highlighted by red and green colors, respectively. Right panel (A, C, D): Heatmap of CTCF (red), BORIS (blue), TBP (purple) a, MXI1 (orange) c, and MAZ (green) d occupancy in NIH3T3+BORIS cells compared to NIH3T3+EV cells at the increased binding sites from the middle panel (connected by red arrow).

## Slide 24
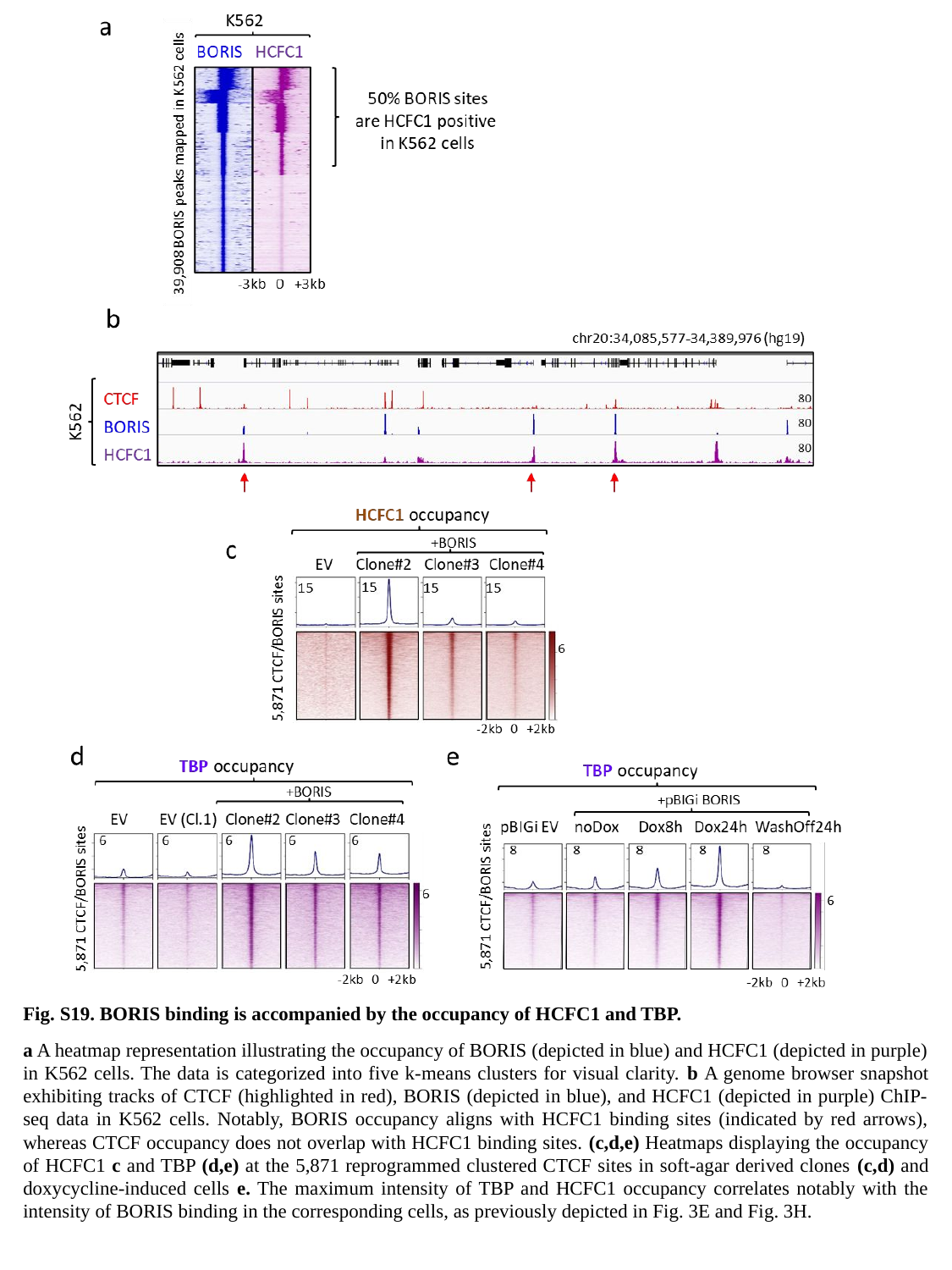

Fig. S19. BORIS binding is accompanied by the occupancy of HCFC1 and TBP.
a A heatmap representation illustrating the occupancy of BORIS (depicted in blue) and HCFC1 (depicted in purple) in K562 cells. The data is categorized into five k-means clusters for visual clarity. b A genome browser snapshot exhibiting tracks of CTCF (highlighted in red), BORIS (depicted in blue), and HCFC1 (depicted in purple) ChIP-seq data in K562 cells. Notably, BORIS occupancy aligns with HCFC1 binding sites (indicated by red arrows), whereas CTCF occupancy does not overlap with HCFC1 binding sites. (c,d,e) Heatmaps displaying the occupancy of HCFC1 c and TBP (d,e) at the 5,871 reprogrammed clustered CTCF sites in soft-agar derived clones (c,d) and doxycycline-induced cells e. The maximum intensity of TBP and HCFC1 occupancy correlates notably with the intensity of BORIS binding in the corresponding cells, as previously depicted in Fig. 3E and Fig. 3H.
