# Supplementary material for: BORIS/CTCFL epigenetically reprograms clustered CTCF binding sites into alternative transcriptional start sites
Source: Genome Biol. 2024 Jan 31;25:40. doi: 10.1186/s13059-024-03175-0 (PMC10832218; doi:10.1186/s13059-024-03175-0)
Supplement: Supplementary file 8 — Additional file 8: Supplementary Table S7. A list of primers sequences used for RT-qPCR. [file 13059_2024_3175_MOESM8_ESM.docx]

| Gene Name | Origin | The sequence of primers | |
| --- | --- | --- | --- |
|  |  | Forward (5’-3’) | Reverse (5’-3”) |
| GAL3ST1 | Human | CTGATTCCAGAAGGTACCT | GAGGCGGGAGGATTGCTT |
| FERT | Human | CGGCTCCCAATCCTGTAAA | CCAGCAATTGAATGACGAATAGA |
| GAPDH | Human | AGCCACATCGCTCAGAGAC | GCCCAATACGACCAAATCC |
| CTAG2 | Human | CAATGCTGGCGGCCCAGGAGAG | CCGCAGGGGCACCTTCCATCCTG |
| MAGEB1 | Human | GAGGGTGAATTCTCAGGACTG | GTTTCTCACGAGCACGGAG |
| PAGE4 | Human | AGAGGAAGAGGAGATGGTCAG | TCTCTTGTCCAGGTTCAATATCC |
| SAGE1 | Human | AGGCTTCTCCACTTCAAACG | TTTGCTTTGATGCCCTGTTG |
| MAGEA8 | Human | GGCTTGAGATCGGCTGAG | AATTGAGACCCACAGGCAG |
| BORIS | Human | GCTGCGGAACCATGTTAACAC | GCCATGTTGCAGTCGTTACACT |
| Gal3st1 | Mouse | GCTGGTCTGCCCCCTACTG | TCCGAGTAGCTTCAATGATGGA |
| Oct4A | Mouse | GTGAAGTTGGAGAAGGTGGAA | GGTGATCCTCTTCTGCTTCAG |
| Oct4B | Mouse | ACTTCTTCAGAATAGGGTGACATT | GCAAACTGTTCTAGCTCCTTCT |
| Gapdh | Mouse | AATGTGTCCGTCGTGGATCTGA | GATGCCTGCTTCACCACCTTCT |
