# Supplementary material for: BORIS/CTCFL epigenetically reprograms clustered CTCF binding sites into alternative transcriptional start sites
Source: Genome Biol. 2024 Jan 31;25:40. doi: 10.1186/s13059-024-03175-0 (PMC10832218; doi:10.1186/s13059-024-03175-0)

Full Images for Westerns in Figure 1

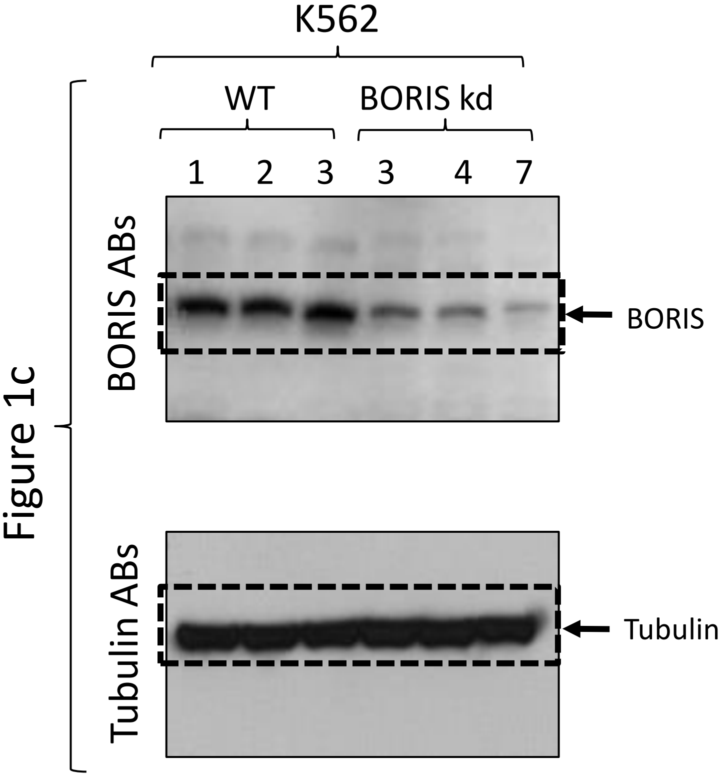

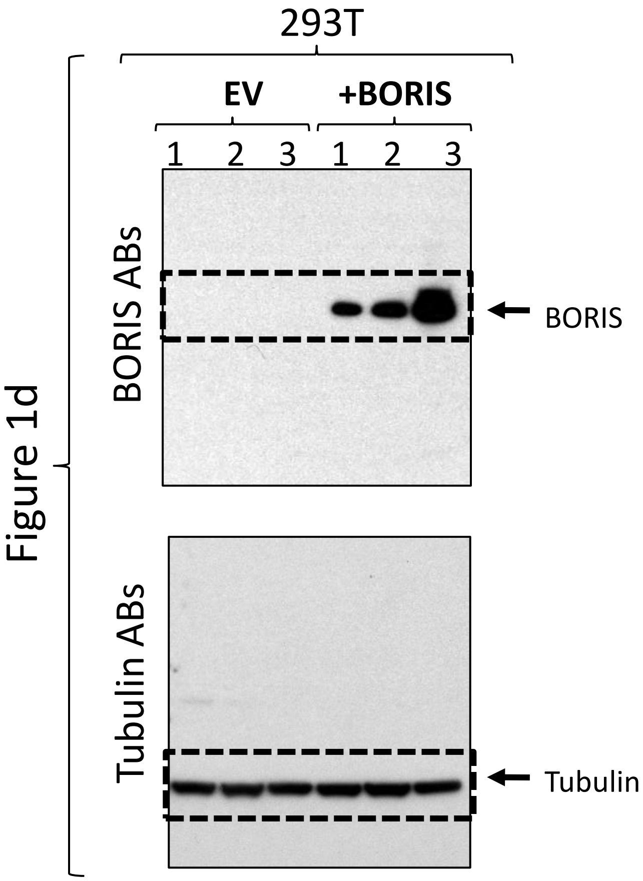

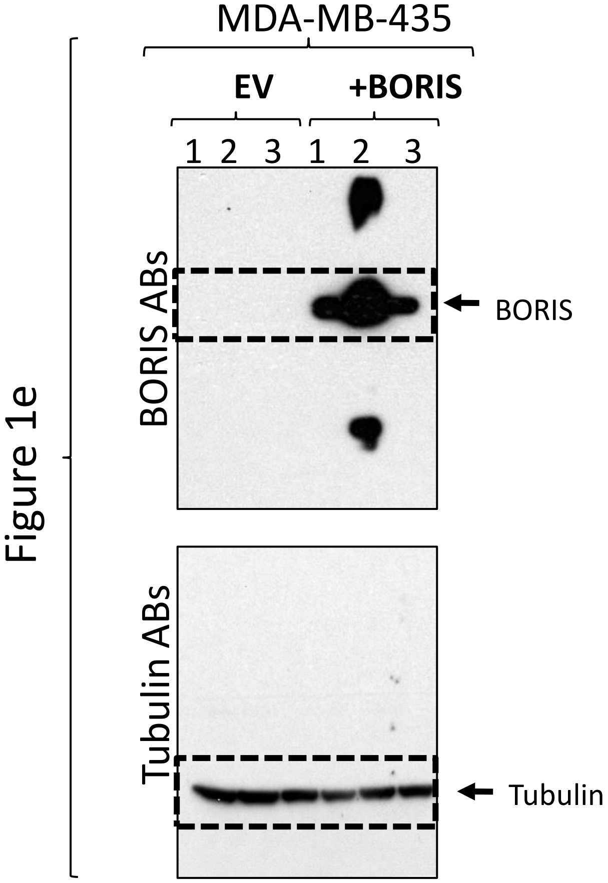

Full Images for Westerns in Figure 3

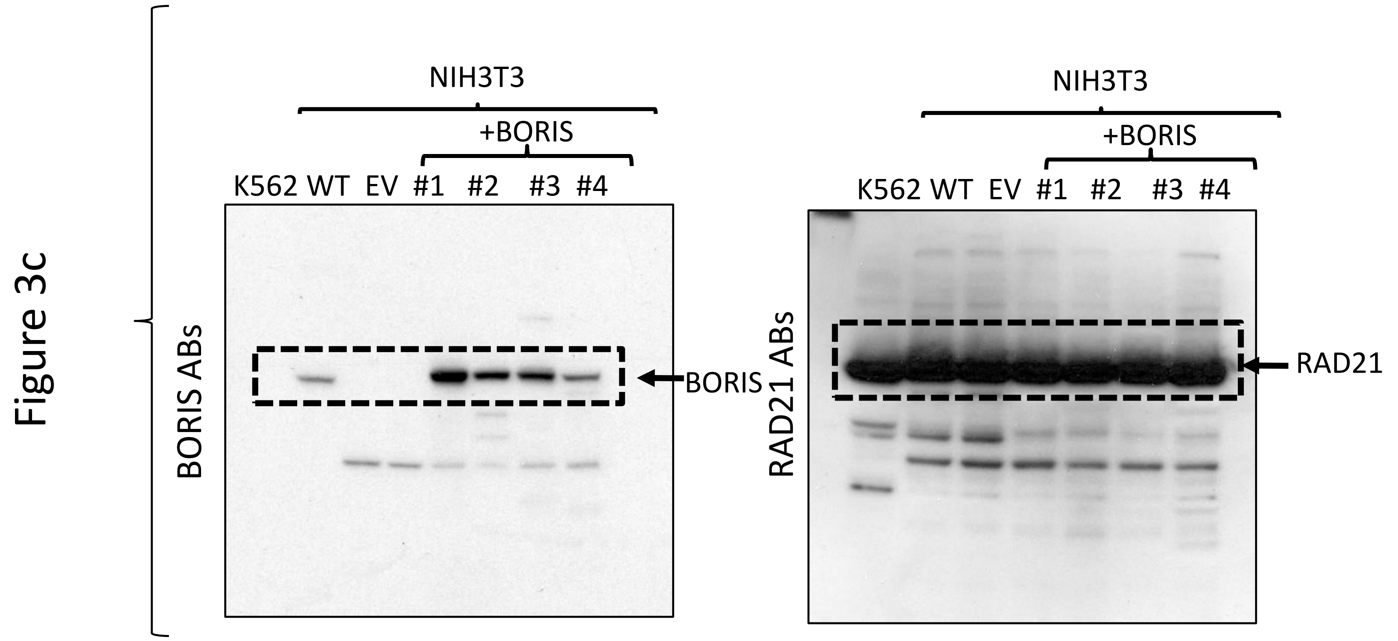

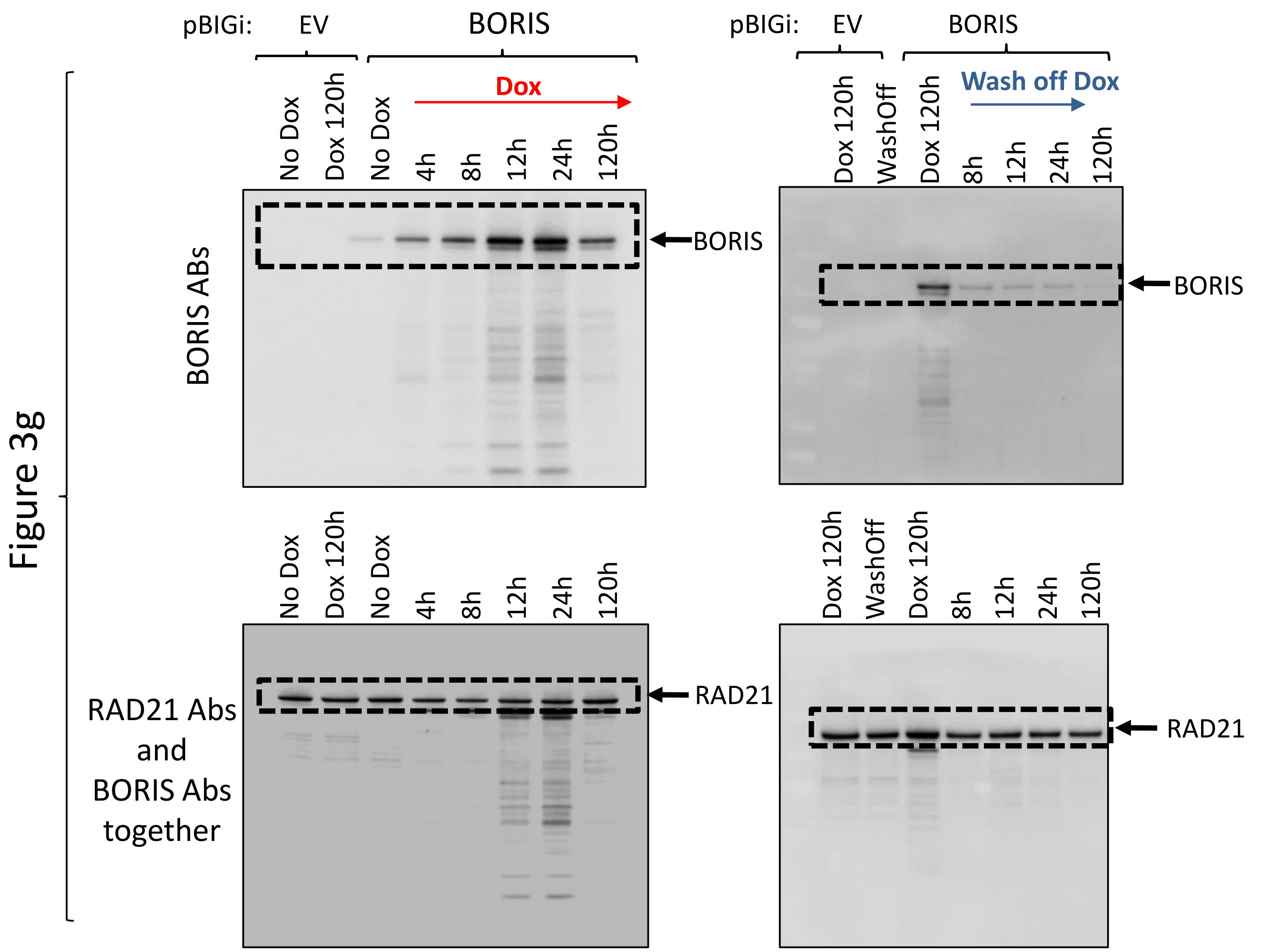

Full Images for Westerns in Figure 5, panel a

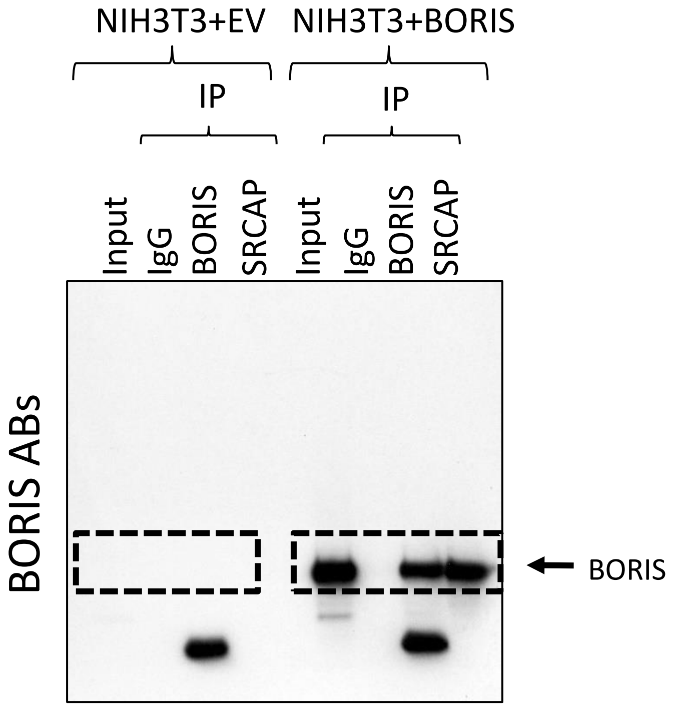

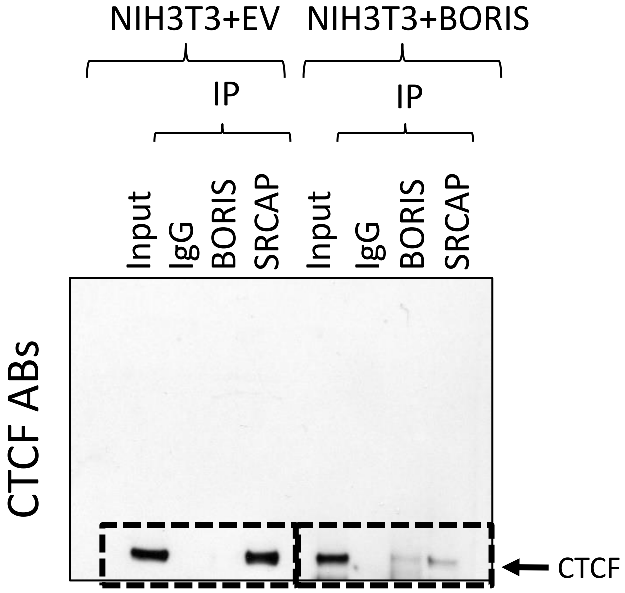

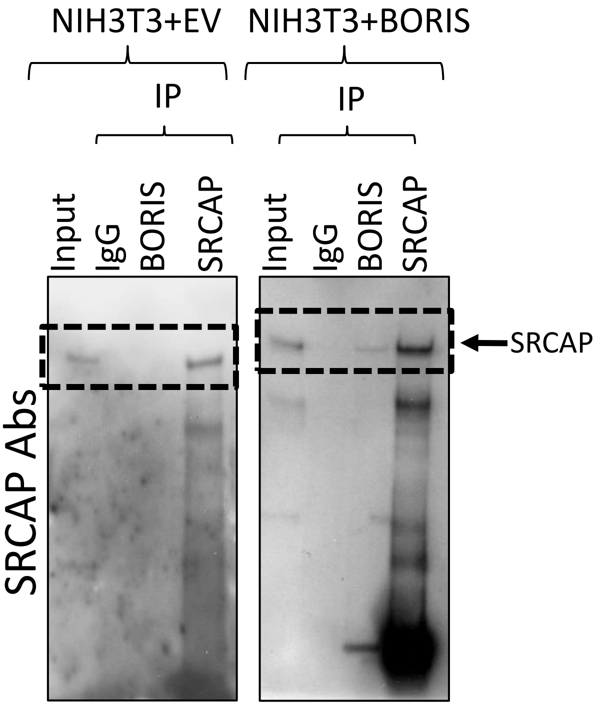

Full Images for Westerns in Figure S8

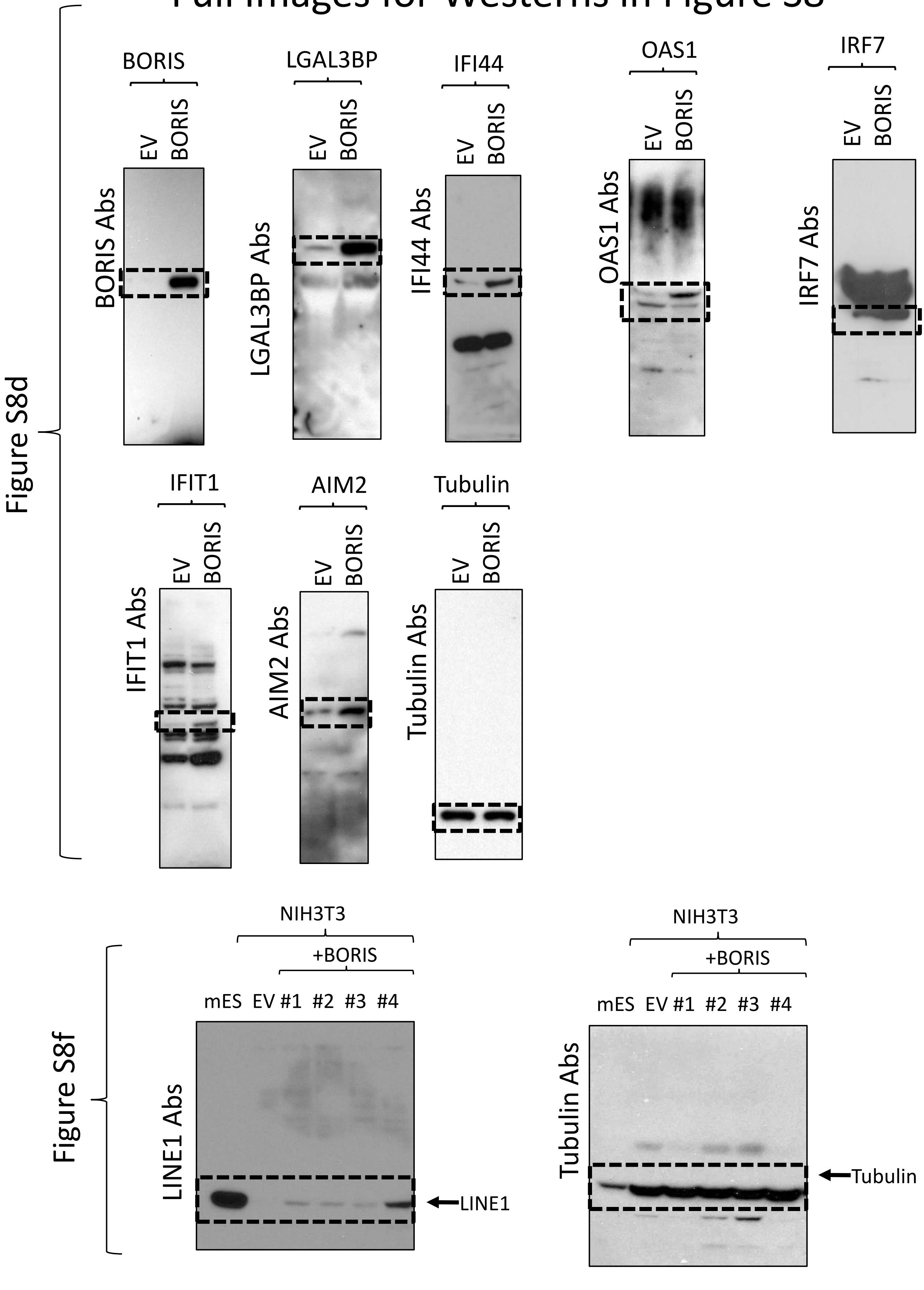

Full Images for Westerns in Figure S14

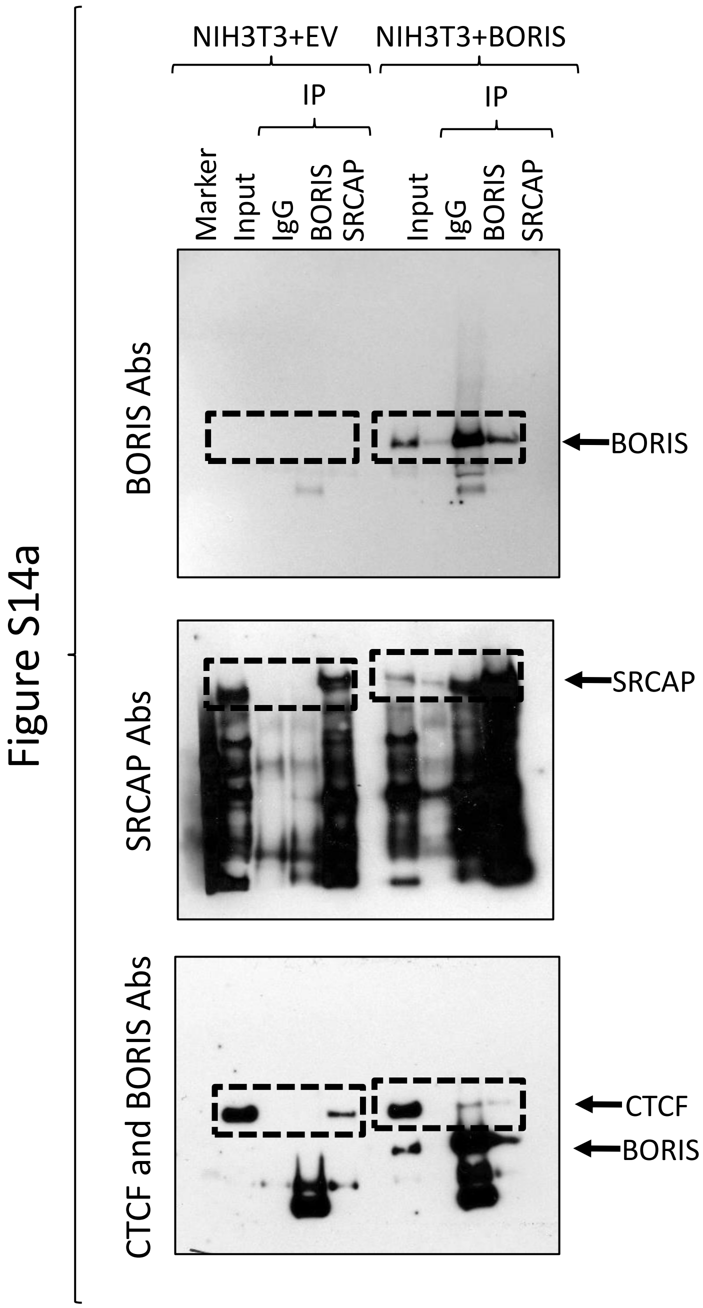

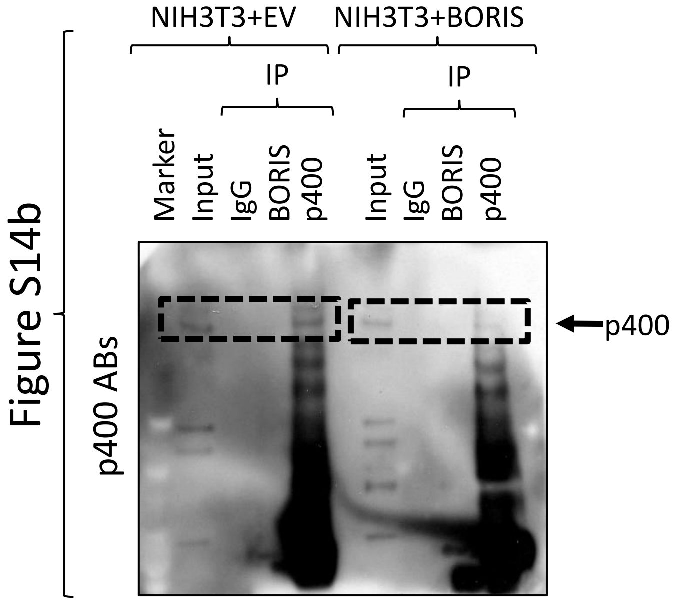

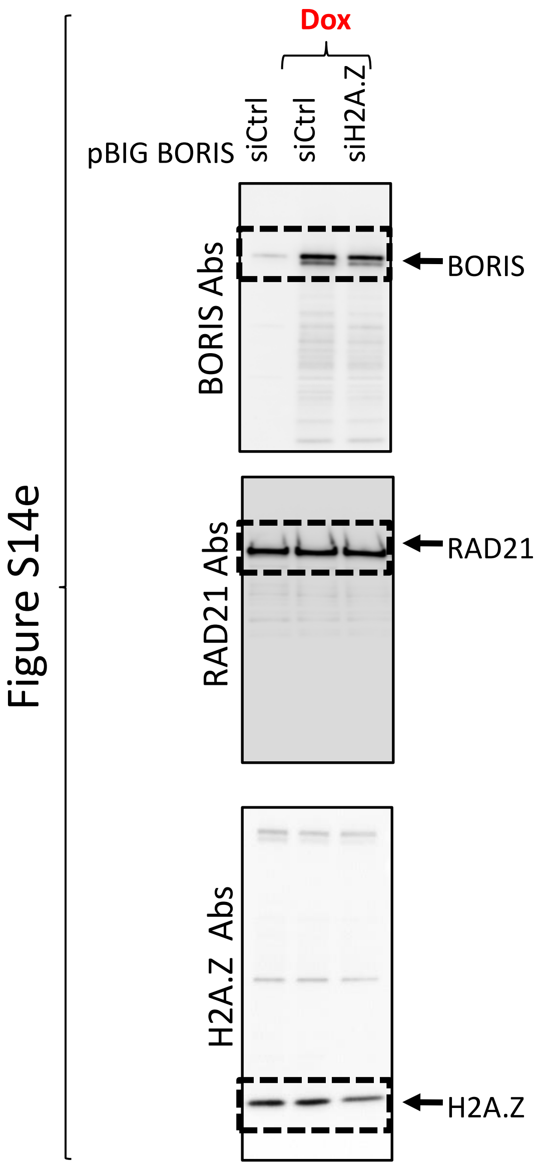

Supplement: Supplementary file 9 — Additional file 9. Uncropped images for the blots in figures 1, 3 and 5 and supplementary figure S8, S14. [file 13059_2024_3175_MOESM9_ESM.pdf]
