# Supplementary material for: BORIS/CTCFL epigenetically reprograms clustered CTCF binding sites into alternative transcriptional start sites
Source: Genome Biol. 2024 Jan 31;25:40. doi: 10.1186/s13059-024-03175-0 (PMC10832218; doi:10.1186/s13059-024-03175-0)
Supplement: Supplementary file 10 — Additional file 10. Review history. [file 13059_2024_3175_MOESM10_ESM.docx]

Review History

**First round of review**

**Reviewer 1**

**Were you able to assess all statistics in the manuscript, including the appropriateness of statistical tests used?**

Yes, and I have assessed the statistics in my report.

**Comments to author:**

This manuscript claims BORIS binding to intronic CTCF binding sites can activate cancer-testis-specific transcription from alternative promoters by recruiting chromatin remodelers and opening chromatin around CTCF sites to allow other complex factors to activate downstream gene transcription. In general, the manuscript was well-written with good-quality data. It is an interesting finding and provides insights into understanding the role of BORIS in the epigenetic regulation of CTCF binding sites and alternative transcription start site activation. Several lines of evidence were provided to address the main conclusion, including genetic manipulation of BORIS by gain and loss of functions, TF, and epigenetic profiling at a genome-wide scale by ChIP-seq and RNA-seq. However, Moreover, I have some concerns regarding scientific rigor with insufficient controls, data interpretation, and potential secondary effects that have hampered my enthusiasm to recommend publishing this manuscript at its current status. I hope my comments below will help the authors strengthen the finding further.

1. The primary concern from me is the secondary effect upon BORIS knockout and ectopic expression. Although the CRISPR knockout clones were used (generated from their Genome Biology Paper in 2015), only one heterozygous clone was used (Clone#7). As stated in the original paper, they could not establish homozygous knockout clones, given the survival crisis. Therefore, the HET clone has been adapted to the knock-down effect. It is very important to derive more HET clones to show reproducibility. Also, I am unsure if "WT" is a clone or bulk. It is more stringent to compare clones with different genotypes. Alternatively, an acute depletion system against BORIS (dTAG or auxin-inducible degron) will provide more information to study BORIS and verify the main conclusion. Within a short period of time to deplete BORIS (hours), they should be able to examine the immediate response of alternative transcription and epigenetic changes.

2. The authors have established the dox-inducible overexpression systems but did not make full use in the study. For instance, samples collected from the time-resolved BORIS induction and washout settings should be included in the RNA-seq and ChIP-seq to determine the direct role of BORIS in alternative promoter activation in selective transcripts. I noticed that authors claimed Both induction with Dox and washing off Dox were continued for five days in total, which is quite a long period for transcription activation. Are there any other time points that they tested?

3. In addition to CAGE and PolII ChIP-seq analysis of the new transcription start usage, I suggest conducting Iso-seq to profile the long-transcript production. In this case, the RNA-seq is based on long-read sequencing without any problems for mapping, which is a powerful tool to confirm the new transcripts from alternative starts which also may share many exons with the reference isoforms.

4. I barely see how many biological replicates were used in ChIP-seq in figures 2, 4, 5, 6, and 7, nor the figure legends. The authors need to provide this information clearly to ensure the producibility and QC analysis, ideally shown in supplementary figures.

**Reviewer 2**

**Were you able to assess all statistics in the manuscript, including the appropriateness of statistical tests used?**

Yes, and I have assessed the statistics in my report.

**Comments to author:**

In their paper, Pugacheva et al. investigate the mechanisms underlying the prevalent use of alternative promoters in germ and cancer cells. This phenomenon is of interest for understanding the fundamental principles of transcription control and germ cell biology, but also has relevance for cancer given that many tumor show a partial activation of the germ cell transcriptional program. One of these genes is BORIS, the focus of the current study. The observation of epigenetic reprogramming of CTCF sites by BORIS is new and well supported by the data, making this an interesting study. Methods chosen are appropriate for answering the research question and properly described.

Comments:

1. BORIS is only able to reprogram a subset of CTCF binding sites in the genome. What makes these sites targetable by BORIS, is that only the dual motif or is there a specific chromatin signature at these sites that could provide additional insight?

2. Fig.S2C: why don't the authors show the expression values of all 1025 BORIS-bound TSS active in K562 & testis here? That would give an impression of how relevant BORIS is for the activation of the larger group.

3. The authors do quite some analyses in which they compare WT vs cl.7 BORIS-KD K562 cells. Working with clones can be tricky, as inter-clonal variation - even in cell lines - is a serious problem. Have the authors validated the effects seen in cl.7 with an independent clone?

**Reviewer 3**

**Were you able to assess all statistics in the manuscript, including the appropriateness of statistical tests used?**

Yes, and I have assessed the statistics in my report.

**Comments to author:**

In this study, the authors explore the role of BORIS in promoting transcriptional changes in cancer cells where it is not normally expressed. They provide a large number of genome-wide datasets and largely through correlative analysis come to the conclusion that BORIS acts at CTCF bound regions to promote epigenetic changes that can activate alternative promoters. There is reasonable evidence to support this central conclusion but the exact mechanism of action remains elusive. The proposed mechanisms involves BORIS-mediated chromatin opening but the only direct evidence is that it recruits SCRAP (known already) and thereby changes H2AZ occupancy. What triggers the other events such as histone mark changes, chromatin opening and TF binding is not clear. Time course analysis will be needed to temporally resolve the order of events and to clearly pinpoint the role of BORIS in this. Technically, the work is generally of high quality but all of the genomic analysis appears to be n=1 from the GEO deposition. Quantitative information cannot be got from n=1, so changes between conditions are hard to interpret. Granted, it appears unlikely that directional changes in all factors tested would occur by change but nevertheless, the agreed standard is a minimum of two biological replicates.

Overall therefore, the observation that BORIS can activate alternative TSSs is interesting and some of the correlative data supports a role for driving epigenetic changes to facilitate this. There are however a number of additional issues that need to be addressed.

Major issues:

(1) This is a long manuscript and the central part gets bogged down with discussing indirect effects on TE expression. This could easily be omitted as detracts from the central story.

(2) What is the evidence that BORIS is acting through clustered CTCF sites rather than simply CTCF sites? It was not apparent that the authors were excluding single CTCF sites in their analysis and/or comparing clustered versus non-clustered. Related to this it would be useful to see some statistics on the co-binding of CTCF and BORIS as determined by ChIP-seq in various systems analysed to support the conclusion that they are working together.

(3) In Fig. 1A there is very little CTCF binding in the absence of BORIS, therefore it is not strictly correct to say that CTCF binding is insufficient for TSS activation. The level here may be critical (as alluded to later on). Some rewording appears warranted.

(4) The authors seem to pick and choose which data fits their model from Fig. 1C. For example, FERT expression is unaffected by BORIS loss. Also, when ectopically expressed in 293T cells, why is there no BORIS binding to this locus? CTCF is there so you would expect sites to be generally available.

(5) In Fig. 2B, there is much lower association with promoter marks in the right two panels compared to the proximal promoters. This could likely be due to enhancer activation. Some clarification in the results text needs adding.

(6) In Fig. 3D, can the authors comment on why clone#1 has the most BORIS expression but the lowest effect on gene expression?

(7) In Fig. 3F clone #4 has the most LIN1 expression but the fewest Tes deregulated. This does not seem to agree with the author's conclusion that LINE1 expression may propagate effects on the expression of other TEs and help explain the pleiotropic indirect effects.

(8) Are the genes analysed in 3G the same genes as in part E? If not what is the overlap in the different experimental conditions/cell systems?

(9) It was not clear from Fig. 3 how this relates to the alternative TSS rather than all BORIS sites. Have the authors performed this analysis as would be useful to add.

(10) From Fig. 4E-I what is the overlap between the BORIS binding sites here? How do these relate to the data in Fig. 2?

(11) For Fig. 4I, the authors state that all are bound by CTCF but you cannot deduce this from a heatmap. A Venn diagram is needed as some may be masked by the signal from other regions.

(12) The authors cannot make the statement in the title of Fig. 6 as the enhanced recruitment of CTCF might be the trigger for chromatin opening. The temporal order of events is impossible to dissect from this data.

(13) In Fig. 7E, the ETS motifs found are enriched at promoters anyway so this is not particularly surprising. If they use all promoters as background, do they still see this enrichment? Also, can the authors say anything here about numbers of CTCF motifs found in the peaks and whether there is any evidence for clustering?

(14) Do other genes for Fig. S2C follow the same pattern as D and E or are these exceptions?

(15) In Fig. S2G, do these genes overlap with the direct targets found in K562 cells at alternative TSSs?

(16) Many of the examples in Fig. S3 show no evidence of CTCF co-binding suggesting that BORIS is acting independently. These observations need incorporating into the overall model.

(17) Fig. S4D does not show a "majority overlap" as stated in the text (<50% in all cases) so this needs rewording. Perhaps more importantly, should there not be more of a focus on the 226 overlapping genes for downstream analysis as higher confidence. If just using clone#2, ~50% genes are not affected in either of the other two lines.

(18) If TEs are within other genes, is it not expected that transcription would be observed as the polymerase will not hop on and off the gene. The same applies to H3K36me3 which is shown in several figures and occurs co-transcriptionally. The significance of Fig. S6 and 7 is not clear and could be omitted.

(19) Fig. S8C-E are all about the indirect consequences of BORIS effects and could be omitted.

(20) Which cell line is used in Fig. S12? Is this inducible or stable? Is it an amalgamation of all three stable lines? Is only one EV control used. This illustrates the problems from having a single ChIP-seq experiment as two different quality EV controls would give a similar result.

Minor issues:

(1) A lot of the supplementary data are barely legible in printed form, and the font sized could be increased in many places.

(2) In Fig. 3E, is the BORIS ChIP-seq done in the same clones? This should be clarified.

(3) In Fig. S1B, the peak for BORIS is not convincing and looks like background noise.

(4) Fig. S5C overlaps are minimal for ncRNAs and should be commented on in the text.

(5) Fig. S13H- text states that these factors are related to carcinogenesis but they are not really and are just general cellular processes.

(6) Fig. S14B. There is no MXI1 peak at the FERT Alt TSS, and likely doubtful at the other locus.

**Authors Response**

**Point-by-point responses to the reviewers’ comments:**

Reviewer #1: This manuscript claims BORIS binding to intronic CTCF binding sites can activate cancer-testis-specific transcription from alternative promoters by recruiting chromatin remodelers and opening chromatin around CTCF sites to allow other complex factors to activate downstream gene transcription. In general, the manuscript was well-written with good-quality data. It is an interesting finding and provides insights into understanding the role of BORIS in the epigenetic regulation of CTCF binding sites and alternative transcription start site activation. Several lines of evidence were provided to address the main conclusion, including genetic manipulation of BORIS by gain and loss of functions, TF, and epigenetic profiling at a genome-wide scale by ChIP-seq and RNA-seq. However, Moreover, I have some concerns regarding scientific rigor with insufficient controls, data interpretation, and potential secondary effects that have hampered my enthusiasm to recommend publishing this manuscript at its current status. I hope my comments below will help the authors strengthen the finding further.

*Reply: We are grateful for the comprehensive review provided by the reviewer on our manuscript. In response to the reviewer's critique, we have included additional datasets, incorporating both biological and technical replicates of Next-Generation Sequencing (NGS) data. We believe that these additional datasets further strengthen our conclusions and enhance the overall quality of the manuscript.*

1. The primary concern from me is the secondary effect upon BORIS knockout and ectopic expression. Although the CRISPR knockout clones were used (generated from their Genome Biology Paper in 2015), only one heterozygous clone was used (Clone#7). As stated in the original paper, they could not establish homozygous knockout clones, given the survival crisis. Therefore, the HET clone has been adapted to the knock-down effect. It is very important to derive more HET clones to show reproducibility. Also, I am unsure if "WT" is a clone or bulk. It is more stringent to compare clones with different genotypes. Alternatively, an acute depletion system against BORIS (dTAG or auxin-inducible degron) will provide more information to study BORIS and verify the main conclusion. Within a short period of time to deplete BORIS (hours), they should be able to examine the immediate response of alternative transcription and epigenetic changes.

*Reply: We appreciate the reviewer for bringing up this question. In 2015, we published a study in Genome Biology (PMID: 26268681), and in 2017 in Oncotarget (PMID: 29088719), where we outlined various models for BORIS downregulation in K562 cells (the cells with a high endogenous BORIS expression). Regardless of the method used to knockout the BORIS gene, we observed a similar result in both models: the K562 cells showed a tendency to differentiate into various lineages with BORIS downregulation. This makes it challenging to use K562 cells to investigate BORIS's role in alternative transcriptional start site usage, as the pathways associated with differentiation overwhelmingly overshadow any transcriptional changes resulting from BORIS downregulation. Nevertheless, employing heterozygous clones proves useful in evaluating BORIS's impact on regulating certain cancer-testis-specific promoters like Gal3ST1 (see Fig. 1C) or CTAG2, MAGEB1, PAGE4, SAGE1, and MAGEA8 (see new Fig. S2F). These genes are well-known cancer-testis antigens and direct BORIS targets, all falling within the list of 1,025 TSSs described in Fig. 2.*

*To address the reviewer's query, we've introduced two additional clones with a partial knockout of BORIS in K562 cells, along with two more single clones for control wild-type (WT) K562 cells (transfected with a single Zinc Finger Nuclease RNA), in addition to a total culture of WT cells. In all three clones with a significant reduction of BORIS protein, we observed a significant decrease in GAL3ST1 expression, but not in FERT gene expression (see new Fig. 1, panel C). Furthermore, we verified that the five cancer-testis genes significantly downregulated in clone #7 also exhibit downregulation in clones #3 and #4, but not in WT clones and the total culture (new Figure S2F).*

*Upon examining the RNA-seq data, we did not observe a widespread downregulation of the 1,025 TSSs in BORIS-depleted K562 cells (clone#7), compared to WT. There could be a few explanations for this: 1). BORIS depletion is partial; 2) The initiation of megakaryocytic differentiation with BORIS depletion could potentially block other pathways; 3). A recent publication suggests that once epigenetic marks are firmly established, they remain stable through cell division, even after the removal of an initiating factor (PMID: 36754048). Thus, even if BORIS binding is lost, the promoter may remain active under CTCF occupancy alone. The FERT promoter serves as an example of this, where the CTCF site in the alternative promoter may sustain transcription despite the loss of BORIS binding (Fig.1C). Although BORIS is necessary to activate FERT transcription in MDA-MB-435 cells (Fig.1E). Based on these observations, we surmise that K562 cells might have limitations when studying the role of BORIS in alternative transcription.*

*We agree with the reviewer that developing an auxin-inducible degron system for BORIS in K562 cells would be an interesting approach. We've made efforts towards generating such a system. Regrettably, targeting BORIS homozygously in K562 cells is a challenge due to the presence of three alleles of BORIS, as we confirmed by karyotyping. Additionally, our experimental observations indicate that these cells tend to differentiate with any genome-editing manipulations. Moreover, we hold reservations about using the auxin-degron system as a means to analyze changes in gene transcription. Firstly, using this system, CTCF knockout resulted in minimal changes in transcription, despite the complete dissolution of the 3D genome structure (PMID: 33626344, PMID: 31127282, PMID: 34429148). Given that most studies establish a significant link between 3D genome organization and gene transcription (PMID: 24905166), there is still no satisfactory explanation for this phenomenon. It may be attributed to yet-to-be-determined artifacts associated with the system.*

2. The authors have established the dox-inducible overexpression systems but did not make full use in the study. For instance, samples collected from the time-resolved BORIS induction and washout settings should be included in the RNA-seq and ChIP-seq to determine the direct role of BORIS in alternative promoter activation in selective transcripts. I noticed that authors claimed Both induction with Dox and washing off Dox were continued for five days in total, which is quite a long period for transcription activation. Are there any other time points that they tested?

*Reply: We appreciate the reviewer's suggestion to include additional time points for dox-induction. This has proven extremely helpful in establishing a direct correlation between BORIS binding, the acquisition of active epigenetic marks, and the initiation of transcription. The updated data can be found in the new main Figures (Fig. 3G-K, 5D) and new Supplementary Figures (Fig. S7, S9B-C, S14D, S19E). Correspondingly, the text has been adjusted to incorporate this description of the new findings.* *Please see the result section: “Ectopic expression of BORIS leads to cellular transformation and the widespread upregulation of transcription.”.*

3. In addition to CAGE and PolII ChIP-seq analysis of the new transcription start usage, I suggest conducting Iso-seq to profile the long-transcript production. In this case, the RNA-seq is based on long-read sequencing without any problems for mapping, which is a powerful tool to confirm the new transcripts from alternative starts which also may share many exons with the reference isoforms.

*Reply: We appreciate the reviewer's valuable suggestion regarding the utility of Iso-seq for mapping isoforms. However, due to budget constraints in our lab, we have had to carefully allocate resources. The primary emphasis of our study is on the conversion of transcriptionally inert CTCF sites into active transcriptional start sites mediated by BORIS recruitment. As such, the gaining of CAGE-seq and PolII ChIP-seq reads, coupled with the mapping of active histone marks (H2A.Z, H3K4me3, H3K27ac) and transcription factors (HCFC1, TBP, MAX, MXI1), suffices to characterize this transition from inactive CTCF binding sites to active promoters. Our RNA-seq data are produced with long reads, typically using 100bp single-end or paired-end reads, or 50 bp paired-end reads, which are sufficient for mapping the majority of isoforms. This approach allowed us to confirm the activation of transcription from these sites.* *Also, in our future experiments we’ll use iso-seq to map isoforms.*

4. I barely see how many biological replicates were used in ChIP-seq in figures 2, 4, 5, 6, and 7, nor the figure legends. The authors need to provide this information clearly to ensure the producibility and QC analysis, ideally shown in supplementary figures.

*Reply: Both ChIP-seq and RNA-seq data, which are presented in the main figures, were generally conducted in at least duplicates or triplicates. We have now included an Excel table as Table S7, containing a comprehensive list of all Next-Generation Sequencing (NGS) data generated in this study. We also edited a text in manuscript that reflect that most of our NGS data are performed in at least duplicates.*

Reviewer #2: In their paper, Pugacheva et al. investigate the mechanisms underlying the prevalent use of alternative promoters in germ and cancer cells. This phenomenon is of interest for understanding the fundamental principles of transcription control and germ cell biology, but also has relevance for cancer given that many tumor show a partial activation of the germ cell transcriptional program. One of these genes is BORIS, the focus of the current study. The observation of epigenetic reprogramming of CTCF sites by BORIS is new and well supported by the data, making this an interesting study. Methods chosen are appropriate for answering the research question and properly described.

*Reply: We thank you the reviewer for positive feedback and for recognizing the significance of our study.*

Comments:

1. BORIS is only able to reprogram a subset of CTCF binding sites in the genome. What makes these sites targetable by BORIS, is that only the dual motif or is there a specific chromatin signature at these sites that could provide additional insight?

*Reply: CTCF and BORIS both possess nearly identical 11 zinc finger domains, allowing them to recognize the same DNA binding targets. Despite this, their genome-wide binding patterns in vivo exhibit a high degree of selectivity: BORIS doesn't compete with CTCF at single motif sites but does form a heterodimer at double motif sites. As we demonstrated in PMID: 26268681, the main prerequisites for a region to be bound by BORIS are: 1) CTCF occupancy and 2) the presence of a double motif (2xCTSes or clustered CTCF sites) beneath the CTCF Chip-seq peak. These conditions create an optimal environment for the formation of a functional heterodimer between CTCF and BORIS.*

*Regarding the CTCF binding sites, which were reprogrammed by BORIS in NIH3T3 cells: First, we conducted ChIP-Re-ChIP and performed motif analysis of CTCF and BORIS co-occupancy in NIH3T3+BORIS cells to confirm that the reprogrammed set of CTCF binding sites belongs to clustered CTCF sites (Please see the new Fig. S10C, S13D-F). Second, we demonstrated that the DNA sequences surrounding the clustered CTCF sites, which were reprogrammed by BORIS, exhibited a substantial and statistically significant enrichment of over 190 DNA binding motifs for other transcription factors to bind (as shown in Figure 7A), in comparison to the clustered CTCF sites that were not reprogrammed. This indicates that the opening of chromatin through H2A.Z histone gain makes the sequences accessible for other transcription factors to bind, thereby facilitating transcription from such reprogrammed sites. Therefore, the sequence surrounding the clustered CTCF sites appears to act as a prerequisite for their epigenetic reprogramming.*

Fig.S2C: why don't the authors show the expression values of all 1,025 BORIS-bound TSS active in K562 & testis here? That would give an impression of how relevant BORIS is for the activation of the larger group.

*Reply: The Fig.S2C (now S2D) illustrates the well-known cancer-testis genes downregulated with BORIS depletion in K562 cells. Given that BORIS remains active in the K562 clone7, and these cells tend to spontaneously differentiate into megakaryocytes (as previously reported in PMID: 26268681), we did not observe a significant impact of BORIS downregulation on the transcription of all 1,025 BORIS-bound TSS in K562. This is exemplified by Fig. 1C where BORIS downregulation results in the silencing of GAL3ST1 expression but does not affect the expression of genes like FERT. One plausible explanation, as discovered in the publication (PMID: 36754048), is that once epigenetic marks are established, they remain stable even after the removal of an initiating factor. Moreover, the RNA-seq analysis of alternative transcripts expressed from intronic promoters is complicated due to their overlap with full-length transcripts. Additionally, after BORIS depletion, cells adapt to a new state of growth. Due to these complexities, we did not encompass the changes in all 1,025 BORIS-bound TSS with BORIS downregulation. Instead, we focused on presenting some CTAs (as shown in Figures 1C and S2D-F), the expression of which was notably affected by BORIS depletion.*

2. The authors do quite some analyses in which they compare WT vs cl.7 BORIS-KD K562 cells. Working with clones can be tricky, as inter-clonal variation - even in cell lines - is a serious problem. Have the authors validated the effects seen in cl.7 with an independent clone?

*Reply: The BORIS-KD clone #7 in K562 cells was initially described in PMID: 26268681. In that study, we utilized zinc finger nuclease (ZFN) technology to target the first coding exon of the BORIS gene in K562 cells. From this effort, we obtained 50 single-cell clones, with 10 of them exhibiting significant BORIS downregulation. Each of the single-cell clones displaying BORIS downregulation demonstrated a consequential differentiation of K562 cells towards the megakaryocytic lineage. Due to its pronounced reduction in BORIS expression, clone #7 was selected for future RNA-seq analysis.*

*However, we have now also included two additional single-cell clones (#3 and #4) with BORIS downregulation in K562 cells, along with two single-cell clones of wild-type (WT) K562 cells derived after the transfection of a single ZFN mRNA (referred to as Control cells). Please see the new panel C in Figure 1 and new panel F in Figure S2 for detailed information.*

*Similar to the clone #7, clones #3 and #4 exhibited a marked downregulation of GAL3ST1 gene expression (new Fig. 1C), but not FERT. Furthermore, the four cancer-testis antigens (CTAs) that were severely downregulated in the clone #7 were similarly downregulated in clones #3 and #4 compared to WT clones (new Fig. S2F). This confirms the dependency of certain CTAs expressions on BORIS binding in cancer cells. What's particularly noteworthy is that upon introducing ectopic BORIS expression in 293T and NIH3T3 cells, the testis-specific alternative promoter for GAL3ST1 is activated from a previously silent state (as demonstrated in Fig. 1D and Fig. 3D). The same holds true for the activation of the FERT promoter in MDA-MB-435 cells upon ectopic BORIS expression (Fig.1E).*

Reviewer #3: Pugacheva et al. Genome Biology. BORIS reprogramming CTCF sites

In this study, the authors explore the role of BORIS in promoting transcriptional changes in cancer cells where it is not normally expressed. They provide a large number of genome-wide datasets and largely through correlative analysis come to the conclusion that BORIS acts at CTCF bound regions to promote epigenetic changes that can activate alternative promoters. There is reasonable evidence to support this central conclusion but the exact mechanism of action remains elusive. The proposed mechanisms involves BORIS-mediated chromatin opening but the only direct evidence is that it recruits SCRAP (known already) and thereby changes H2A.Z occupancy. What triggers the other events such as histone mark changes, chromatin opening and TF binding is not clear. Time course analysis will be needed to temporally resolve the order of events and to clearly pinpoint the role of BORIS in this. Technically, the work is generally of high quality but all of the genomic analysis appears to be

n=1 from the GEO deposition. Quantitative information cannot be got from n=1, so changes between conditions are hard to interpret. Granted, it appears unlikely that directional changes in all factors tested would occur by change but nevertheless, the agreed standard is a minimum of two biological replicates.

Overall therefore, the observation that BORIS can activate alternative TSSs is interesting and some of the correlative data supports a role for driving epigenetic changes to facilitate this. There are however a number of additional issues that need to be addressed.

*Reply: We sincerely appreciate the reviewer's thoughtful and constructive feedback on our study. In response to the critique, we have made significant additions to our data. We now present evidence demonstrating that H2A.Z histone gain at CTCF/BORIS sites is indeed crucial for alternative promoter activation (Please see the new Figure S14E-H). Furthermore, we have extended our dox-induction experiment to include more time points, as illustrated in the new Figure 3G-K, 5D and S7. This extended time course analysis not only allowed us to establish a direct time- and dose-dependent gene deregulation by a direct BORIS binding, but also revealed that the gain of H2A.Z histone precedes the acquisition of H3K4me3 at the alternative promoters (Please see new Figure S14D). This reinforces our initial conclusion regarding the pioneering role of H2A.Z in this process. To validate the epigenetic reprogramming of clustered CTCF sites with a broader range of biological and technical replicates, we expanded the genome-wide analysis of epigenetics to include two additional NIH3T3+BORIS clones (#3 and #4) (Please see the new Figure S13A-C, S19C,D) and one additional clone for NIH3T3+EV (Clone#1) (new Figure 3D, S5A, S13B and S19D).*

*In terms of genomic analysis, we want to emphasize that most of our Next-Generation Sequencing (NGS) data have been conducted in at least duplicates, and in some cases, in triplicates. The confusion regarding the single replicate (n=1) might have emerged due to our submission of raw (FASTQ files) data for all replicates, alongside a single file that combined two replicates as analyzed data. To address this, we have now included an Excel table encompassing a comprehensive list of all NGS data generated in this study as supplementary table S7. Furthermore, we have revised the manuscript text to accurately reflect that most of our ChIP-seq experiments are performed in at least duplicates or have biological replicates.*

*We are pleased to hear that you find our work technically sound and of high quality. Your comments and suggestions are invaluable in guiding our future research efforts.*

**Major issues:**

(1) **This is a long manuscript and the central part gets bogged down with discussing indirect effects on TE expression. This could easily be omitted as detracts from the central story**.

*Reply: Following the recommendation, we have completely reworked this section, significantly reducing the TE content in this part of the manuscript. We maintain our belief in the significance of this portion, as it may elucidate the activation of inflammation pathways in BORIS-expressing cells via the AMI2-mediated pathway. Additionally, the activation of LINE1 repeats by BORIS from a previously silent state is of considerable interest in addressing BORIS' role in transposon activity, as a continuation of our previous project (PMID: 27588042).*

(2) What is the evidence that BORIS is acting through clustered CTCF sites rather than simply CTCF sites? It was not apparent that the authors were excluding single CTCF sites in their analysis and/or comparing clustered versus non-clustered. Related to this it would be useful to see some statistics on the co-binding of CTCF and BORIS as determined by ChIP-seq in various systems analysed to support the conclusion that they are working together.

*Reply: The clustered CTCF sites were initially detailed in our previous publication (PMID: 26268681). In that study, we conducted genome-wide mapping of CTCF and BORIS binding sites in several cancer cell lines with endogenous BORIS expression (K562, Delta47, and OVCAR8), as well as in mouse testes. This led us to the conclusion that CTCF and BORIS form heterodimers at sequences containing two or more CTCF binding motifs, which we termed 2xCTSes (CTCF Target Sites) or clustered CTCF sites.*

*To address the reviewer's query about BORIS acting through clustered CTCF sites, we performed a ChIP-Re-ChIP assay, involving sequential chromatin immunoprecipitations using first BORIS antibodies and followed by CTCF antibodies. Additionally, we conducted motif analysis of CTCF and BORIS occupancy in NIH3T3+BORIS (clone#2) cells (Please see the new Fig. S13D-F, S10C). Out of the 5,871 sites epigenetically reprogrammed by BORIS, 88% exhibited overlap with the sites immunoprecipitated through sequential chromatin immunoprecipitations with both CTCF and BORIS antibodies (Fig. S13D). Furthermore, 45% of these sites displayed the presence of at least two equal CTCF motifs under ChIP-seq peaks, indicating their belonging to the clustered CTCF sites (new Fig. S13F). Of note, as we demonstrated in our prior study (PMID: 26268681), determining the presence of two CTCF motifs under the summit of CTCF ChIP-seq peak can be complicated due to slight variations in different CTCF motifs. In this analysis, we focused on one type of CTCF motif, which still showed a significant enrichment of double motifs in BORIS bound sites, compared to CTCF-only sites (Please see the new Fig.S13F).*

(3) In Fig. 1A there is very little CTCF binding in the absence of BORIS, therefore it is not strictly correct to say that CTCF binding is insufficient for TSS activation. The level here may be critical (as alluded to later on). Some rewording appears warranted.

*Reply: Accordingly, we added this point: “Based on FERT and GAL3ST1 gene expression analyses in BORIS-positive (K562) and BORIS-negative (NHEK) cells, CTCF occupancy alone, or low CTCF occupancy, is not sufficient to drive the transcription from the two corresponding intronic promoters (Fig.1A-B).”*

(4) The authors seem to pick and choose which data fits their model from Fig. 1C. For example, FERT expression is unaffected by BORIS loss. Also, when ectopically expressed in 293T cells, why is there no BORIS binding to this locus? CTCF is there so you would expect sites to be generally available.

*Reply: We have conducted a thorough analysis of two well-published genes, GAL3ST1 and FER, regarding their alternative transcription mediated by BORIS. In Figure 1C-E, we present detailed evidence across three cell lines (K562, 293T, MDA-MB-435) with varying levels of BORIS expression. Our findings demonstrate that both testis-specific transcripts from these genes are under the regulation of BORIS binding, although there are cell context-dependent variations. Figure 1C illustrates that the loss of BORIS binding in K562 cells results in the loss of GAL3ST1 expression, but not FERT. The FERT promoter continues to express under CTCF occupancy alone in K562 BORIS-depleted cells. One plausible explanation for this phenomenon is that once epigenetic marks are established, they tend to remain stable even when the initial cause of the epigenetic changes is removed, as demonstrated in a recent study (PMID: 36754048).*

*In the case of 293T cells, the extent of BORIS binding to chromatin is a crucial factor. In 293T cells, BORIS binding is considerably weaker compared to K562. Please see the heatmap of BORIS occupancy by ChIP-seq in these cell lines (Figure below). As a result, not all potential sites are occupied by BORIS, as exemplified by the FERT promoter in Fig.1D.*

(5) In Fig. 2B, there is much lower association with promoter marks in the right two panels compared to the proximal promoters. This could likely be due to enhancer activation. Some clarification in the results text needs adding.

*Reply: Yes, as illustrated in Fig. 2B, both intragenic and intergenic BORIS binding sites exhibit lower enrichment of active transcription marks (RNAPII, H3K4me3, CAGEs) compared to BORIS sites located in the reference gene promoters. To address the reviewer’s inquiry, we overlapped these three classes of BORIS sites with chromatin interactions associated with active enhancers in the same K562 cells, utilizing ChIA-PET data for H3K27ac in K562 cells (GSM1436262). This analysis has been integrated into the text, along with the supplementary new Figure S2B (and is depicted below). Specifically, we found that the BORIS sites in close proximity to promoters are significantly more engaged (82%) in interactions with enhancers marked by H3K27ac, in comparison to intragenic (42%) and intergenic (32%) BORIS binding sites.*

*Figure S2B: Percentage representation of BORIS sites overlapping with H3K27ac-marked anchors in K562 cells, mapped by ChIA-PET with H3K27ac antibodies.*

(6) In Fig. 3D, can the authors comment on why clone#1 has the most BORIS expression but the lowest effect on gene expression?

*Reply: Although Western blotting is a powerful method for demonstrating BORIS activation, it is less quantitative compared to qPCR analysis. We have now included qPCR analysis as a new supplementary Figure S5A. According to the qPCR analysis, BORIS expression is highest in clone #2 compared to the other clones, which correlates with the highest Gal3st1 activation. Figure 3D illustrates that Gal3st1 is activated in all four clones, in contrast to the empty vector (EV) control. However, the level of Gal3st1 activation varies among the clones. Upon analyzing the RNA-seq data for the NIH3T3+BORIS clones #2, #3, and #4, we observed some variations in gene deregulation among the individual single-cell clones, as exemplified by the variation in LINE1 activation in BORIS-expressing clones (Fig.S 8F).*

(7) In Fig. 3F clone #4 has the most LIN1 expression but the fewest Tes deregulated. This does not seem to agree with the author's conclusion that LINE1 expression may propagate effects on the expression of other TEs and help explain the pleiotropic indirect effects.

*Reply: LINE1 does not have a direct influence on the expression of TEs (Transposable Elements). TEs are mobilized through a 'copy-and-paste' mechanism facilitated by LINE1 (PMID: 30454069).*

*The significant increase in LINE1 expression observed in the clone #4 (now in the new Fig.S8F) aligns well with the heightened activation of Aim2 (inflammasome-based pathway) in the same clone #4 (as depicted in Fig.S8E). Aim2 is a protein stimulated by the enrichment of cytosolic double-stranded DNA (PMID: 19158675). The activation of LINE1 leads to an accumulation of DNA double-strand breaks (as reported in PMID: 16490214), establishing a connection between LINE1, Aim2, and the inflammatory pathway activation. In this context, the data are consistent and supportive of this relationship. We have now emphasized this in the text related to this: “It's worth highlighting that the activation of the autonomous LINE1 is particularly significant, as LINE1 has the capability to mobilize other transposable elements (TEs) [12] and induce DNA double-strand breaks [61], connecting the activation of LINE1 with the upregulation of Aim2 observed in the clone #4.”*

(8) Are the genes analysed in 3G the same genes as in part E? If not what is the overlap in the different experimental conditions/cell systems?

*Reply: A total of 354 genes (34%) were found to be upregulated, while 56 genes (11.5%) were downregulated in both conditions (soft-agar derived clones and dox-induced BORIS expression). This shared gene deregulation in two different conditions is highly significant, with a p-value of less than 0.00001 as determined by the Fisher test. For further details, please see the new Figure S 7C.*

(9) It was not clear from Fig. 3 how this relates to the alternative TSS rather than all BORIS sites. Have the authors performed this analysis as would be useful to add.

*Reply: Figure S13G displays the genomic distribution of 5,871 epigenetically reprogrammed CTCF sites by BORIS. Specifically, 12% of these sites are located in RefSeq gene promoters, 41% in intragenic regions, and 47% in intergenic regions. This indicates that the majority of BORIS-reprogrammed sites are associated with alternative transcription rather than canonical transcription, at least in NIH3T3+BORIS cells, derived from soft-agar.*

*Figure 3 (and its corresponding supplementary Figures S5) provides an overview of all kinds of transcripts and pathways that are deregulated by ectopic BORIS expression. BORIS activates transcription from transcriptionally silent CTCF sites residing in both canonical and alternative promoters. Notably, Figure 2D indicates that 48% of BORIS sites are associated with canonical promoters in respect to cancer-testis transcription. Therefore, this study encompasses not only alternative transcription, but also demonstrates that numerous Cancer Testis Antigens (CTAs) are regulated by BORIS from canonical promoters (see Fig.S2D-I), utilizing the same mechanism through the reprogramming of CTCF sites.*

*Figure 4 (and its associated supplementary Figures S9, S10, S15B,C, S16C,I) is dedicated to a more detailed analysis of deregulated transcripts, with a focus on alternative transcription.*

(10) From Fig. 4E-I what is the overlap between the BORIS binding sites here? How do these relate to the data in Fig. 2?

*Reply: Panels E-G (as well as Fig.S12) illustrate the gain (at least two-fold) of active histone marks, CAGEs, and RNAPII at BORIS sites. All these changes are combined into a single display in panels H-I of Fig.4, illustrating the convergence of various marks associated with active transcription at BORIS binding sites. In terms of its relation to Figure 2, the genomic distribution of the 5,871 sites is depicted in new Figure S13G.*

(11) For Fig. 4I, the authors state that all are bound by CTCF but you cannot deduce this from a heatmap. A Venn diagram is needed as some may be masked by the signal from other regions.

*Reply: We corrected the text: “Fig.4H shows that ~~all of~~ these sites are bound by only CTCF in NIH3T3+EV cells and depleted of any marks of active transcription”. Additionally, we have included a Venn diagram (please see the new Fig. S13D) which demonstrates the overlap between the 5,871 sites and the occupancy of both CTCF and BORIS in NIH3T3+BORIS (clone#2) cells*.

(12) The authors cannot make the statement in the title of Fig. 6 as the enhanced recruitment of CTCF might be the trigger for chromatin opening. The temporal order of events is impossible to dissect from this data.

*Reply: We agree with the reviewer. While it may be challenging to precisely distinguish the temporal sequence of events from this data, it's evident that BORIS binding induces both changes: enhanced enrichment of CTCF occupancy and the opening of chromatin around its binding sites. These changes are specifically detected at BORIS bound sites, and not elsewhere (Fig.6 B-E). In light of this, we have adjusted the title to: “BORIS expression is associated with a significant increase in CTCF occupancy and a more open chromatin state around CTCF site.” We believe that this revised title accurately reflects the findings.*

(13) In Fig. 7E, the ETS motifs found are enriched at promoters anyway so this is not particularly surprising. If they use all promoters as background, do they still see this enrichment? Also, can the authors say anything here about numbers of CTCF motifs found in the peaks and whether there is any evidence for clustering?

*Reply: Figure 7E displays the most enriched motifs for the transcription factors under HCFC1 peaks, as mapped in both NIH3T3+EV and NIH+BORIS cells. In the case of BORIS binding in NIH3T3+BORIS cells, we observed the emergence of 19,519 new HCFC1 peaks associated with BORIS binding. The purpose of Figure 7E is to demonstrate that these new HCFC1 peaks are linked to CTCF/BORIS binding sites, in contrast to EV.*

*The number of CTCF motifs has been addressed in the new Figure S13D-F and is described in the corresponding results section.*

(14) Do other genes for Fig. S2C follow the same pattern as D and E or are these exceptions?

*Reply: Figure S2C (now S2D) showcases the most notable examples of Cancer-Testis Antigens (CTAs), which are substantially downregulated with BORIS downregulation in K562 cells. It's worth noting that not all genes followed this pattern, as illustrated in Figure 1C, where GAL3ST1 was downregulated in K562 BORIS kd cells, but FERT was not. One plausible explanation, as elucidated in the publication (PMID: 36754048), is that once epigenetic marks are established, they may remain stable even when the initial cause of the epigenetic changes is removed.*

In Fig. S2G, do these genes overlap with the direct targets found in K562 cells at alternative TSSs?

*Reply: Figure S2G (now S2H) illustrates the upregulation of cancer-testis (CT) genes in MDA-MB-435 (a melanoma cancer cell line) with ectopic BORIS expression, as compared to EV. In Figure S2I below, we demonstrate that the activation of CT genes is accompanied by direct BORIS binding to CTCF sites. These sites were silent under CTCF occupancy alone, reproducing a similar pattern as seen in NIH3T3 cells. Out of the 60 genes shown in Figure S2G, 7 overlap with the 1,025 TSS bound in K562 cells. In general, we observed that the impact of BORIS is dependent on cell-specific context, as reflected in Figures 1C-E.*

(15) Many of the examples in Fig. S3 show no evidence of CTCF co-binding suggesting that BORIS is acting independently. These observations need incorporating into the overall model.

*Reply: That is correct in the sense that BORIS-only sites are also associated with active transcription, as exemplified in Figure S3. The question about BORIS-only sites was addressed in more detail in our previous publication (PMID: 26268681). BORIS-only sites belong to the clustered CTCF sites that could be occupied by either both CTCF and BORIS, or solely by BORIS, or solely by CTCF, depending on BORIS expression level and cell context. This is also reflected in our main Figures 1C-E, where the clustered CTCF site in the GAL3ST1 alternative promoter is bound by CTCF only in K562 BORIS-depleted cells (Figure 1C), by both CTCF and BORIS in 293T+BORIS cells (Figure 1D), and by BORIS-only in MDA-MB-435+BORIS cells (Figure 1E). To explain this, we’ve added some text about BORIS-only sites in Discussion: “ In this study, we demonstrated, for the first time, that BORIS functions akin to pioneer transcription factors by binding to chromatin. BORIS exhibits two distinct mechanisms for epigenetic remodeling, depending on the initial state of corresponding sites. The primary and most prevalent method involves its incorporation into transcriptionally silent CTCF-bound sites. Subsequently, these sites undergo epigenetic remodeling into active promoters through the recruitment of SRCAP, H2A.Z, and various transcription factors. The second mechanism involves BORIS binding to CTCF motifs not already bound by CTCF, which are within closed chromatin regions. Here, BORIS either recruits CTCF to these sites or binds independently (referred to as BORIS-only sites), resulting in chromatin remodeling in the vicinity. In both scenarios, the interaction between CTCF and BORIS emerges as pivotal for the epigenetic conversion of CTCF sites into functional promoters. It is noteworthy that in certain instances, BORIS-only sites also demonstrate an association with active transcription.”*

(17)	Fig. S4D does not show a "majority overlap" as stated in the text (<50% in all cases) so this needs rewording. Perhaps more importantly, should there not be more of a focus on the 226 overlapping genes for downstream analysis as higher confidence. If just using clone#2, ~50% genes are not affected in either of the other two lines.

*Reply: Clone #2 of NIH3T3+BORIS exhibited the most profound transcriptional changes, which correlate strongly with the highest BORIS expression and occupancy in the clone#2, compared to other clones (please see the new Fig. 3E-F, S5). The overlapping patterns among the three clones are statistically significant (Fisher test, P < 0.00001), as we now stated in the text, instead of using the term 'majority overlap'.*

(18)	If TEs are within other genes, is it not expected that transcription would be observed as the polymerase will not hop on and off the gene. The same applies to H3K36me3 which is shown in several figures and occurs co-transcriptionally. The significance of Fig. S6 and 7 is not clear and could be omitted.

*Reply: We have followed the reviewers' recommendation and omitted Fig. S6 and S7. Additionally, the section on TEs has been significantly downsized.*

(19)	Fig. S8C-E are all about the indirect consequences of BORIS effects and could be omitted.

*Reply: Figure S8 C-E (now Fig. S8D-G) confirms the activation of inflammation pathways with ectopic BORIS expression in different cell lines of both human and mouse origin. We consider this figure essential for our understanding of the aberrant BORIS expression in cancers, where inflammation pathways and cancer progression are closely interconnected*.

(20)	Which cell line is used in Fig. S12? Is this inducible or stable? Is it an amalgamation of all three stable lines? Is only one EV control used. This illustrates the problems from having a single ChIP-seq experiment as two different quality EV controls would give a similar result.

*Reply: We are sorry for this confusion. Fig. S12 (now Fig. S15) displays the data for NIH3T3+BORIS (clone #2, soft-agar derived clone), compared to NIH3T3+EV (total culture, selected under antibiotic pressure). We have now included this clarification in the corresponding text and Figure legend. Regarding EV, we used a total culture selected under antibiotic pressure as a control. However, we have also selected two single-cell clones with EV under antibiotic pressure. These two clones are now presented in Fig. 3D and Fig. S5A. Furthermore, we conducted ChIP-seq experiments in the single-cell EV clone (clone#1),* *focusing on the two most modified marks associated with active transcription in BORIS-expressing cells, H2A.Z (new Fig. S13B) and TBP (new Fig. S19D). These data for single-cell EV clone were compared to the EV total culture. Our analysis did not reveal any significant changes in the epigenetic marks between the EV total culture and the EV single clone. These ChIP-seq data have been added to the GEO accession number: GSE207058 (token: klofsyocbfmpfep).*

*As previously mentioned, the confusion regarding single ChIP-seq experiments arose from submitting the raw data (FASTQ files) for all replicates, while providing only one file, combining two replicates, for each condition as the analyzed data, following recommendation from GEO. It's important to note that the majority of our Next-Generation Sequencing (NGS) data is generated in duplicates or triplicates.*

**Minor issues:**

(1) A lot of the supplementary data are barely legible in printed form, and the font sized could be increased in many places.

*Reply: All our figures have been created in PowerPoint with a focus on maintaining a high resolution. Unfortunately, we've noticed that the resolution significantly decreased when the files were converted to Word and then subsequently to PDF format. To avoid this issue, we have decided to submit the supplementary data directly in PDF format to ensure that the resolution remains optimal throughout the review process. We also increased a font size throughout.*

(2) In Fig. 3E, is the BORIS ChIP-seq done in the same clones? This should be clarified.

*Reply: Yes, BORIS ChIP-seq was conducted with replicates for all three single-cell clones (#2, #3, #4). We have now integrated these datasets into new panel E of Figure 3.*

(3) In Fig. S1B, the peak for BORIS is not convincing and looks like background noise.

*Reply: Indeed, while the binding of BORIS in 293T cells may appear relatively modest, it remains significantly enriched in comparison to input levels. Importantly, this level of binding is sufficient to activate GAL3ST1 expression.*

(4) Fig. S5C overlaps are minimal for ncRNAs and should be commented on in the text.

*Reply: The Figure 5S related to the overlap of genes deregulated in two human melanoma cell lines (MDA-MB-435+BORIS and MM057+BORIS). In accordance with the reviewer's suggestion, we have significantly reduced the section related to noncoding transcripts, ultimately opting to remove this portion entirely.*

(5) Fig. S13H- text states that these factors are related to carcinogenesis but they are not really and are just general cellular processes.

*Reply: Yes, these are general cellular processes, which are highly upregulated in cancer. For example, just to name a few with regard to their connection to cancer: Tight Junction Proteins and Signaling Pathways in Cancer and Inflammation (PMID: 30728783), Oxytocin signaling pathway (PMID: 30254962), AMPK signaling pathway (PMID: 25812084), Glycerophospholipid metabolism (PMID: 21222647). We have rephrased this sentence as follow: “Analysis of the newly accessible sites in NIH3T3+BORIS cells revealed a general association with BORIS binding, an increase in active histone marks (Fig. 6E, upper panel), and the activation of gene transcription (Fig. S16E,F), related to the cellular processes upregulated in carcinogenesis (Fig. S16H).”*

(6) Fig. S14B. There is no MXI1 peak at the FERT Alt TSS, and likely doubtful at the other locus.

*Reply: Yes, we agree with the reviewer's observation that MXI binding is relatively weak at the GAL3ST1 alternative promoter and absent at the FERT. However, based on now Figure S17C, it is evident that MXI is significantly enriched at the 1,025 transcription start sites (TSSs) in K562 cells. Additionally, its motif is enriched at the 5,871 epigenetically reprogrammed sites (Figure 7A-B). We have updated the corresponding text to accurately reflect this: “Among these factors, both MAZ and MXI1 were among the 190 TFs enriched at the 5,871 CTCF/BORIS sites (Fig. 7B), with MAZ protein binding to both the intronic GAL3ST1 and FERT promoters in K562 cells, along with BORIS (Fig. S17D)”.*

**Second round of review**

**Reviewer 1**

In the revised manuscript, authors have addressed my previous concerns about the replicate number and inducible system by providing more single-cell derived clones and time-dependent doxycycline inducible samples. These new samples were well characterized in the following cellular and ChIP-seq assays. They also provided reasonable argument about AID system and long-read sequencing. Overall, I feel the major conclusion of the manuscript was significantly strengthened with these additional efforts. I only have three minor comments to polish the manuscript.

1. The ChIP-seq scale in the lower panel of Fig1C (bottom) should be provided appropriately same as other panels.
2. Font and text size in all of the figures should be consistent. I saw different types of size and font. In particular, text size of scale bar of ChIP-seq heatmaps are too small to visualize. e.g., Fig6E, Fig7D.
3. Schematic diagram in Fig7G can be further optimized to better summarize the main conclusion.

**Reviewer 2**

The authors have adequately addressed my comments.

**Reviewer 3**

This is an improved manuscript with additional data added, superfluous information removed and further clarifications added. There are however a few issues that need tidying up.

First the number of replicates. I checked the Table and there are still some that are single replicates. It is important to clarify the conclusions appropriately from these experiments. Also, The M&M needs updating to say how replicates were handled. What was the criteria for combining datasets? Were any not deemed suitable? As a minimum the correlation values between replicates should be added for ChIP- and ATAC-seq, and these should conform to ENCODE recommendations otherwise combining is not possible.

Other points (original numbering used):

(4) The authors have answered my point but not altered the manuscript text to reflect their response. It is still not clear why FERT only responds to BORIS in certain cells but this should be highlighted in the text.

(6) the explanation given by the authors is not rational. Protein levels reflect the amounts of BPRIS available to bind chromatin. RNA levels do not. Yes it might be more quantitative but it is pretty clear that clone1 has way more than clone 4 for example. I can accept the fact that strange clonal differences elsewhere in the cell may do this and it would be good to add this explanation to the text as a rationale for then focussing on clones 2-4.

(10) My question was about the number of BORIS sites that show increases in all three marks. This could be done by a Venn diagram. Heatmaps just show a trend.

(15) The explanation is okay but the authors should comment on the cell-type specific effects in the text.

(17) Just a comment really. I can see why they focussed on all the data in clone 2 but the overlapping genes between different clones would have provided a more high confidence dataset.

**Authors Response**

**Point-by-point responses to the reviewers’ comments:**

Reviewer #1: In the revised manuscript, authors have addressed my previous concerns about the replicate number and inducible system by providing more single-cell derived clones and time-dependent doxycycline inducible samples. These new samples were well characterized in the following cellular and ChIP-seq assays. They also provided reasonable argument about AID system and long-read sequencing. Overall, I feel the major conclusion of the manuscript was significantly strengthened with these additional efforts. I only have three minor comments to polish the manuscript.

*Reply: Thank you very much for the comprehensive review and the reviewer’s constructive feedback.*

1. The ChIP-seq scale in the lower panel of Fig1C (bottom) should be provided appropriately same as other panels.

*Reply: We have made this correction, according to the reviewer’s recommendation.*

2. Font and text size in all of the figures should be consistent. I saw different types of size and font. In particular, text size of scale bar of ChIP-seq heatmaps are too small to visualize. e.g., Fig6E, Fig7D.

*Reply: We have adjusted the text size in the Figures 2B, 4E, 6E, and 7D, according to the reviewer’s recommendation.*

3. Schematic diagram in Fig7G can be further optimized to better summarize the main conclusion.

*Reply: We have optimized the schematic diagram in Fig.7G.*
*Reply: Thank you once again for your time and valuable input.*

Reviewer #2:
The authors have adequately addressed my comments.
 *Reply: We extend our sincere thanks to the reviewer for the comprehensive review and continuous support throughout this review process.*

Reviewer #3: This is an improved manuscript with additional data added, superfluous information removed and further clarifications added. There are however a few issues that need tidying up.

*Reply: We greatly appreciate the time and effort the reviewer invested in evaluating our work.*

*First the number of replicates. I checked the Table and there are still some that are single replicates. It is important to clarify the conclusions appropriately from these experiments.*

*The main conclusions drawn in our research are primarily supported by data obtained from at least two replicates across RNA-seq, ATAC-seq, and CAGE-seq analyses. While most ChIP-seq assays were conducted with at least two replicates, some were performed with a single replicate to complement and further substantiate our primary findings, which were confirmed by data sets with replicates or biologically replicated experiments. For example, we showed that BORIS binding (ChIP-seq, 3 replicates) opens chromatin (ATAC-seq, 3 replicates) around CTCF binding sites (ChIP-seq, 3 replicates) by the recruitment of SRCAP (ChIP-seq, 2 replicates), followed by the replacement of H2A histone with H2A.Z (ChIP-seq, 3 replicates), resulting in gain of active histone marks (H3K4me3, H3K27ac, ChIP-seq, 3 replicates for each ChIP) and RNAPII recruitment (ChIP-seq with 3 replicates). In addition, our study illustrates that the relaxation of chromatin around CTCF binding sites promotes the recruitment of additional transcription factors, notably TPB and HCFC1, each with two replicates. For further validation of observed trends, we included PHF8, MAZ, and MXI1, employing a single replicate for both empty vector (EV) and BORIS-expressing cells to save cost and time. These singular replicates were specifically designed to complement and fortify our primary findings. However, the NGS data generated from these singular replicates could be omitted from the study without impacting the final conclusions. Additionally, we conducted several BORIS ChIP-seq experiments using a single replicate for BORIS-negative cells. These data sets served as supplementary evidence, bolstering our conclusions drawn from other assays like Western blotting and qRT-PCR, thereby affirming the specificity of BORIS antibodies.*

Also, The M&M needs updating to say how replicates were handled. What was the criteria for combining datasets? Were any not deemed suitable? As a minimum the correlation values between replicates should be added for ChIP- and ATAC-seq, and these should conform to ENCODE recommendations otherwise combining is not possible.

*Reply: We thank you the reviewer for the specific recommendations regarding the Methods and Materials section. We updated the Methods section to include a detailed description of how replicates were handled. Of note, the combination of technical replicates was done only for ChIP-seq data to call the final ChIP-seq peaks. We have now added in Methods part: for ChIP-seq: “The reproducibility of ChIP-seq data between technical replicates was assessed by computing the Pearson correlation coefficient (PCC) based on mapped read counts within MACS-called genomic regions. Initially, the MACS2 peak calling algorithm was applied independently for each replicate. Subsequently, the called ChIP-seq peaks from each replicate were merged into one combined set to calculate the PCC between replicates. All ChIP-seq data replicates exhibited an optimal range of correlation, typically higher than 0.85 to 0.95, ensuring robust reproducibility. To call the final ChIP-seq peaks, the two BAM file replicates were merged,* *duplicate reads were removed, and the MACS2 algorithm was applied” and for ATAC-seq: “The evaluation of ATAC-seq data reproducibility through Pearson correlation coefficients among three technical replicates demonstrated an optimal value of greater than 0.9”.*

Other points (original numbering used):

(4) The authors have answered my point but not altered the manuscript text to reflect their response. It is still not clear why FERT only responds to BORIS in certain cells but this should be highlighted in the text.

*Reply: We have updated Fig. S1 to incorporate an additional panel illustrating the relatively low BORIS binding in 293T cells. This addition aims to provide insight into the absence of BORIS binding at the FERT alternative promoter, which subsequently leads to its lack of activation. Our text has been revised accordingly: “ At the same time, the FERT promoter was significantly activated in MDA-MB-435, but not in HEK293T cells. The integration of ChIP-seq with RNA-seq data showed that the differential activation of the two testis-specific promoters depends on their co-occupancy by CTCF and BORIS proteins. Specifically, the absence of BORIS binding at the FERT promoter in HEK293T cells, likely due to comparatively lower BORIS occupancy (Additional file 1: Fig. S1d), suggests its lack of activation.”*

(6) the explanation given by the authors is not rational. Protein levels reflect the amounts of BPRIS available to bind chromatin. RNA levels do not. Yes it might be more quantitative but it is pretty clear that clone1 has way more than clone 4 for example. I can accept the fact that strange clonal differences elsewhere in the cell may do this and it would be good to add this explanation to the text as a rationale for then focussing on clones 2-4.

*Reply: We agree with the reviewer's assessment. Regrettably, our focus on the clone#1 was limited as it did not exhibit a dramatic Gal3st1 activation. We believe that the current description in the text aligns well with our rationale: “To investigate whether BORIS activates cancer-testis specific transcription in somatic cells, we analyzed Gal3st1 expression in NIH3T3 cells expressing either EV or BORIS. We observed a robust induction of Gal3st1 expression in all four BORIS-expressing clones, with the highest activation in clones #2, #3, and #4 (Fig. 3d). These three clones were chosen for further genome-wide analyses using ChIP-seq and RNA-seq to evaluate the transcriptomic and epigenetic changes following ectopic BORIS expression.”*

(10) My question was about the number of BORIS sites that show increases in all three marks. This could be done by a Venn diagram. Heatmaps just show a trend.

*Reply: While Venn diagrams can be useful in certain contexts, they might not be the most accurate representation for Figure 4. Our attempts to create a Venn diagram were less illustrative and convincing. Therefore, we propose using a heatmap to better illustrate trends in this case.*

(15) The explanation is okay but the authors should comment on the cell-type specific effects in the text.

*Reply: The cell-specific effects are detailed in Figures 1C-E. The discussion in the text begins with the statement,
“The activation of intronic promoters was also dependent on cell-specific context:….”*

(17) Just a comment really. I can see why they focussed on all the data in clone 2 but the overlapping genes between different clones would have provided a more high confidence dataset.

*Reply: Yes, we agree with the reviewer. The overlapping of genes in the three clones is shown in Figure S5E.*
